# Effect of Timing and Coordination Training on Mobility and Physical Activity Among Community-Dwelling Older Adults: A Randomized Clinical Trial

**DOI:** 10.1001/jamanetworkopen.2022.12921

**Published:** 2022-05-23

**Authors:** Jennifer S. Brach, Subashan Perera, Valerie Shuman, Alexandra B. Gil, Andrea Kriska, Neelesh K. Nadkarni, Bonny Rockette-Wagner, Rakie Cham, Jessie M. VanSwearingen

**Affiliations:** 1 Department of Physical Therapy, University of Pittsburgh, Pittsburgh, Pennsylvania; 2 Department of Epidemiology, University of Pittsburgh, Pittsburgh, Pennsylvania; 3 Division of Geriatric Medicine, Department of Medicine, University of Pittsburgh, Pittsburgh, Pennsylvania; 4 Department of Biostatistics, University of Pittsburgh, Pittsburgh, Pennsylvania; 5 Department of Neurology, University of Pittsburgh, Pittsburgh, Pennsylvania; 6 Department of Bioengineering, University of Pittsburgh, Pittsburgh, Pennsylvania

## Abstract

**Importance:**

Standard exercise interventions targeting underlying physiologic system impairments have limited success in improving walking. Augmenting standard interventions with timing and coordination training, which incorporates the principles of motor learning and integrates multiple systems, may be more successful.

**Objective:**

To determine whether a standard strength and endurance program incorporating timing and coordination training (standard-plus) improves gait speed more than strength and endurance training alone.

**Design, Setting, and Participants:**

The Program to Improve Mobility in Aging (PRIMA) study was an assessor-blinded, randomized, 2-group intervention trial that included a 12-week intervention and 24-week follow-up period. The trial was conducted at a university research clinic from 2016 to 2020. Participants included 249 community-dwelling older adults (aged ≥65 years) with gait speed between 0.60 and 1.20 m/s. Statistical analysis was performed from December 2020 to March 2021.

**Interventions:**

Participants were randomized to standard strength and endurance (n = 125) or standard-plus, including timing and coordination training (n = 124), 50 to 60 minutes, twice a week for 12 weeks.

**Main Outcomes and Measures:**

Primary outcome of gait speed and secondary outcomes representing components of the intervention (leg strength and power, 6-minute walk test, chair sit-and-reach test, and figure of 8 walk test) and activity and participation (Late Life Function and Disability Instrument and daily physical activity measured by accelerometry) were measured at 12, 24, and 36 weeks.

**Results:**

Among 249 randomized participants, 163 (65.5%) were female, 22 (8.8%) were Black, 219 (88.0%) were White; mean (SD) age was 77.4 (6.6) years; mean (SD) gait speed was 1.07 (0.16) m/s; and 244 (98.0%) completed the intervention. The 2 groups did not have significantly different improvements in gait speed or secondary outcomes representing the components of the intervention at any time point. For gait speed, individuals in the standard-plus group had a mean (SD) improvement of 0.079 (0.135) m/s over 12 weeks, 0.065 m/s (0.141) over 24 weeks, and 0.059 (0.150) m/s over 36 weeks; individuals in the standard group improved gait speed by 0.081 (0.124) m/s over 12 weeks, 0.051 (0.129) m/s over 24 weeks, and 0.065 (0.148) m/s over 36 weeks.

**Conclusions and Relevance:**

This randomized clinical trial found no difference in gait speed change between the standard and standard-plus intervention groups, and both groups showed sustained improvements in mobility 24 weeks after the intervention.

**Trial Registration:**

ClinicalTrials.gov Identifier: NCT02663778

## Key Points

**Question:**

What is the effect of adding timing and coordination training to a standard strength and endurance program on mobility in community-dwelling older adults?

**Findings:**

In this randomized clinical trial that included 249 older adults, treatment with either the standard program or the program enhanced with timing and coordination training resulted in improved gait speed. There was no difference between groups.

**Meaning:**

These findings suggest that timing and coordination training may not yield additional benefit compared with a standard program to improve walking among older adults.

## Teaser

This randomized clinical trial examines whether the addition of timing and coordination training to a standard exercise program for strength and endurance improved walking among community-dwelling older adults more than strength and endurance training alone.

## Introduction

Difficulty walking often leads to disability and is associated with negative health outcomes such as hospitalization, nursing home placement, and death.^1,2^ Approximately half of community-dwelling older adults have difficulty walking and in those without difficulty, 22% will develop new walking difficulty over the next year.^3^

Exercise is beneficial to walking; however, studies examining interventions to improve walking in older adults in general demonstrate limited success. Gains in walking speed rarely reach thresholds for clinically important gains,^4^ even when compared with passive control groups.^5,6^ When compared with active controls, meaningful between-group differences disappear as both groups improve.^7,8,9^ Moreover, postintervention gains are not sustained long-term.^5,8,10^ The benefits of exercise to walking in frail older adults or older adults with substantially impaired mobility are more robust.^11,12^ In a meta-analysis of the effect of exercise in frail older adults, the exercise group increased their gait speed by 0.07 m/s more than the control group, which was both statistically significant and clinically meaningful.^11^ Likewise, the LIFE study found that a moderate intensity physical activity program reduced mobility disability over a 2.6-year period.^12^

Walking is a complex task that requires the integration of multiple physiologic systems.^13^ Interventions to improve walking primarily target musculoskeletal and cardiopulmonary systems through strength and endurance training, but rarely address the nervous system through timing and coordination training.^14^ In pilot studies, interventions with timing and coordination components resulted in greater improvements in walking than standard programs.^15,16,17^ These trials were not definitive, however, and both intervention impact on other outcomes and the persistence of benefits are unknown. Our objectives were to (1) determine whether a standard strength and endurance program incorporating timing and coordination training (standard-plus) improved walking more than a standard program, (2) determine whether benefits would be observed in both those with slow and near normal gait speed and persist over time, and (3) examine association with intervention component-related, activity-related, and participation-related outcomes. We anticipated individuals in the standard-plus program would have greater improvements in walking, activity, and participation and be more likely to sustain gains over time.

## Methods

This randomized clinical trial followed the Consolidated Standards of Reporting Trials (CONSORT) reporting guideline. The study protocol is included in Supplement 1. The University of Pittsburgh institutional review board approved the protocol and participants provided written informed consent.

### Study Design

The Program to Improve Mobility in Aging (PRIMA) study was an assessor-blinded, randomized, 2-group intervention trial of a 12-week intervention with a 24-week follow-up that took place from 2016 to 2020. The trial methods are published in detail^18^ and registered.

### Participants

Enrollment of community-dwelling older adults occurred between 2016 and 2019. Inclusion criteria were: (1) aged at least 65 years, (2) ambulatory without assistance of a device or person, (3) usual gait speed of 0.60 to 1.2 m/s, and (4) physician clearance. Individuals with medical conditions that made testing or participation in exercise unsafe or plans to leave the area during the study period were excluded (eTable 1 in Supplement 2).

We recruited participants through the Pittsburgh Pepper Center Research Registry, screening for eligibility over the phone and in-person. We randomized participants 1:1 to intervention groups using random block sizes, stratified by gait speed (ie, slower [<1.0 m/s] or faster [≥1.0 m/s]). Participants self-reported race and gender. Race was collected to describe the sample. Options given for race included American Indian or Alaskan Native, Asian, Black or African American, Native Hawaiian or other Pacific Islander, White, other, or refused.

### Interventions

For both groups, physical therapist–supervised exercise sessions were 50 to 60 minutes twice weekly for 12 weeks. All participants received a physical activity behavioral change intervention based on the Group Lifestyle Balance program.^19^

The standard intervention included a brief warm-up, lower extremity strengthening exercise, endurance exercise, and a cooldown period. Strengthening exercises were conducted on stacked weight training equipment and included: knee extension, knee flexion, leg press, hip abduction, and hip extension. Once participants completed 2 sets of 15 repetitions with light effort (ie, Rating of Perceived Exertion [RPE] < 10),^20^ resistance was increased with the goal of exercising at RPE 11 to 13, or “somewhat hard.” The endurance exercise consisted of treadmill walking at RPE 10 to 13 or “somewhat difficult workload.” When participants could tolerate 15 minutes, the workload was increased. The overall goal was to achieve 40 minutes of continuous treadmill walking exercise at the “somewhat difficult” level.

Standard-plus participants completed task-specific timing and coordination exercises in addition to standard intervention. The timing and coordination exercise included goal-oriented, progressively more difficult stepping and walking patterns important for gait. Progression was based on separately increasing the speed, amplitude, or accuracy of performance and by completing more complex tasks.^21^ To keep the total walking and standing time equal between the 2 groups, participants in the standard-plus intervention spent less time in endurance training.

Modified Group Lifestyle Balance (mGLB) is a behavioral lifestyle intervention to improve health through eating and physical activity modification.^19^ All participated in 16 mGLB (physical activity component only) education sessions. The first 12 sessions were weekly, followed by 2 sessions every other week, and 2 monthly sessions (1 each month).

### Measurements and Procedures

Data collection was repeated at baseline, and 12, 24, and 36 weeks. All data were collected by assessors who were blinded to intervention group.

#### Primary Outcome

Gait speed is a reliable, valid, and sensitive mobility outcome representing functional ability^4,22,23^ assessed with an instrumented walkway (Zeno Walkway, Protokinetics LLC, Havertown, Pennsylvania). Small and substantial meaningful change criteria were 0.05 m/s and 0.10 m/s, respectively.^4^

#### Secondary Outcomes

To assess the integrity of the intervention, we included measures representing intervention components (strength, endurance, flexibility, timing, and coordination) and additional mobility measures. Leg strength and power were measured using the Keiser A420 (Keiser Corporation, Fresno, California) pneumatic leg press.^24^ The highest recorded strength/power of all repetitions from a side was recorded at baseline and on the same side at follow-up. We assessed walking endurance with the 6-minute walk test (6MWD).^25^ The chair sit-and-reach test was the measure of (hamstring) flexibility.^26^ Values can be positive or negative, positive numbers indicating greater flexibility. Timing and coordination were assessed using the figure of 8 walk (F8W) test.^27^ Performance is scored on time to complete and number of steps taken. Composite lower extremity function was assessed using the short physical performance battery (SPPB),^28^ and confidence in walking using the modified gait efficacy scale (mGES).^29^

#### Tertiary Outcomes

We measured self-reported activity and participation, as defined by International Classification of Functioning, Disability and Health (ICF) framework,^30^ with the Late Life Function and Disability Instrument (LLFDI).^31,32^ We measured performance-based daily physical activity with Actigraph GT3X accelerometers (Actigraph, Pensacola, Florida). Participants wore accelerometers on their waist during waking hours for 7 consecutive days, recording wear time. The triaxial accelerometer is an electronic sensor that measures both the quantity and intensity of movement, resulting in the collection of daily patterns of activity. We focused on (1) vector magnitude (counts/d), (2) moderate and vigorous activity (min/d), (3) sedentary behavior (min/d), and (4) steps taken (steps/d).

### Sample Size and Power

See published protocol for details.^18^ Briefly, 248 participants were deemed adequate to detect a 0.10 m/s between-group difference in gait speed with greater than 99% statistical power, with 93% power within each stratum (slower/faster, assuming an even split), greater than 80% power within each stratum (if the split were no worse than 33% to 67%). We planned to detect small to moderate differences in F8W and LLFDI with 80% power.

### Statistical Analysis

See published protocol for details.^18^ We compared baseline characteristics between groups using independent samples *t* tests, χ^2^ tests, and Fisher exact tests. For main results, we performed an intention-to-treat analysis with multiple imputation for missing data.^33^ We fit linear mixed models with change from baseline in each continuous outcome measure as the dependent variable; intervention group, follow-up time point, and their interaction as fixed effects; baseline value of outcome as a fixed effect covariate; and a participant random effect. For outcomes based on accelerometers, wear time was an additional covariate. We constructed means contrasts to compare the intervention gains at each of the follow-up time points. We repeated the analysis with gait speed stratum and stratum × intervention as additional fixed effects to obtain stratum-specific findings. We used *P *< .05 2-sided tests for statistical significance. Statistical analysis was performed using SAS software version 9.4 (SAS Institute) from December 2020 to March 2021.

## Results

Among 249 randomized participants, 163 (65.5%) were female, 22 (8.8%) were Black, 219 (88.0%) were White; mean (SD) age was 77.4 (6.6) years; mean (SD) gait speed was 1.07 (0.16) m/s; and 244 (98.0%) completed the intervention. The groups were similar at baseline and more than one-third reported a fall in the prior year (Table 1). Secondary to equipment failure and lost devices, 240 individuals (96.3%) had physical activity data at 1 or more time points. Those with physical activity data tended to have better performance and function and more cancer history (gait speed: 1.08 m/s vs 0.93 m/s; LLFDI overall function: 60.6 points vs 49.7 points; cancer: 35.4% vs 11.1%). However, they were similar between the intervention groups except Trails B time (eTable 2 in Supplement 2).

**Table 1.** Participant Characteristics and Measures at Baseline

Characteristic	Participants, No. (%)	
	Standard-plus (n = 124)	Standard (n = 125)
Age, mean (SD), y	77.4 (6.7)	77.4 (6.4)
Gender		
Female	85 (68.6)	78 (62.4)
Male	39 (31.5)	47 (37.6)
Race		
Asian	1 (0.8)	0
Black or African American	9 (7.3)	13 (10.4)
Native Hawaiian or other Pacific Islander	1 (0.8)	1 (0.8)
White	109 (87.9)	110 (88.0)
Other^a^	1 (0.8)	0
Refused to say	3 (2.4)	1 (0.8)
Live alone	57 (46.0)	57 (45.6)
Marital status		
Married	60 (48.4)	56 (44.8)
Separated	0	2 (1.6)
Divorced	19 (15.3)	16 (12.8)
Widowed	28 (22.6)	35 (28.0)
Never married	13 (10.5)	15 (12.0)
Other	4 (3.2)	1 (0.8)
Education		
Grade 9-12	22 (17.7)	19 (15.2)
College	47 (37.9)	42 (33.6)
Postgraduate	52 (41.9)	59 (47.2)
Other	3 (2.4)	5 (4.0)
Duke comorbidity index, range 0-8, mean (SD)^b^	2.9 (1.3)	2.9 (1.3)
Cardiac	12 (9.7)	12 (9.6)
Neurologic	10 (8.1)	7 (5.6)
Musculoskeletal	107 (86.3)	108 (86.4)
General	33 (26.6)	38 (30.4)
Visual/hearing	94 (75.8)	98 (78.4)
Diabetes	28 (22.6)	25 (20.0)
Cancer	41 (33.1)	45 (36.0)
Lung	34 (27.4)	31 (24.8)
Geriatric Depressive Scale, range 0-15, mean (SD)^c^	1.0 (1.3)	1.1 (1.4)
Modified Mini-Mental State, mean (SD)	95.8 (4.3)	96.0 (4.0)
Trails A, mean (SD), s	33.1 (12.7)	34.3 (13.1)
Trails B, mean (SD), s	82.0 (45.1)	89.2 (46.1)
Height, mean (SD), in	65.2 (3.8)	65.6 (4.0)
Weight, mean (SD), lb	174.7 (38.9)	174.8 (35.9)
Body mass index, mean (SD)	28.8 (5.7)	28.6 (5.9)
Fear of falling	50 (40.3)	51 (40.8)
Fall prior year	36 (29.0)	38 (30.4)
More than 1 fall prior year	13 (10.5)	14 (11.2)
Global mobility rating		
Excellent	25 (20.2)	17 (13.6)
Very good	52 (41.9)	49 (39.2)
Good	36 (29.0)	41 (32.8)
Fair	11 (8.9)	16 (12.8)
Poor	0	2 (1.6)
Global health rating		
Excellent	16 (12.9)	22 (17.6)
Very good	66 (53.2)	53 (42.4)
Good	37 (29.8)	38 (30.4)
Fair	5 (4.0)	10 (8.0)
Poor	0	2 (1.6)
Global balance rating		
Excellent	5 (4.0)	7 (5.6)
Very good	27 (21.8)	28 (22.4)
Good	55 (44.4)	50 (40.0)
Fair	32 (25.8)	33 (26.4)
Poor	5 (4.0)	7 (5.6)
Instrumented walkway gait speed, mean (SD), m/s	1.08 (0.16)	1.06 (0.17)
Gait speed stratum		
<1.0 m/s	52 (41.9)	52 (41.6)
≥1.0 m/s	72 (58.1)	73 (58.4)
Modified gait efficacy scale, range 10-100, mean (SD)	85.5 (13.3)	84.8 (14.0)
Short Physical Performance Battery, mean (SD)	9.9 (1.4)	9.8 (1.7)
Lower extremity strength 1 RM [greater of the 2 sides], mean (SD), N	183.8 (55.0)	188.3 (60.8)
Lower extremity power [greater of the 2 sides], mean (SD), W	364.8 (128.7)	371.0 (174.4)
Six-minute walk distance, mean (SD), meters	397.6 (91.7)	400.9 (88.3)
Figure of 8 walk, mean (SD)		
Time to complete, s	9.9 (1.9)	10.2 (2.3)
No. of steps	17.1 (2.8)	17.2 (2.9)
Chair reach test, centimeters	−5.0 (10.7)	−7.2 (10.7)
Late Life Function and Disability Index, range 0-100, mean (SD)^d^		
Overall function	61.7 (8.8)	61.2 (8.3)
Upper extremity function	78.7 (10.5)	78.4 (11.7)
Basic lower extremity function	75.0 (13.4)	73.9 (12.4)
Advanced lower extremity function	52.0 (14.0)	51.6 (13.3)
Disability frequency	55.5 (5.9)	55.0 (5.4)
Social role	51.4 (7.4)	50.7 (7.3)
Personal role	68.8 (15.6)	67.3 (13.9)
Instrumental role	79.4 (13.4)	78.8 (13.5)
Management role	92.7 (10.6)	91.5 (10.3)
Disability limitations	79.5 (12.7)	78.8 (12.9)

Abbreviation: RM, repetition maximum.

^a^ For the category of race, one of the options that participants could select was other. American Indian and Alaska Native are included in other.

^b^ Higher numbers indicate more comorbidities.

^c^ Higher numbers indicate more depressive symptoms.

^d^ Higher numbers indicate better function and less disability.

See Figure for participant flow and sources of missing data. By phone, 523 were screened; 353 were eligible and assessed in person. Of these 353, 97 failed and 7 withdrew. Of the continuing 249 participants, 124 were randomized to the standard-plus group and 125 to the standard group. Of the 124 participants in the standard-plus intervention group, 122 (98.4%) completed the postintervention testing; of the 125 participants in the standard intervention group, 122 (97.6%) completed the postintervention testing.

**Figure.**
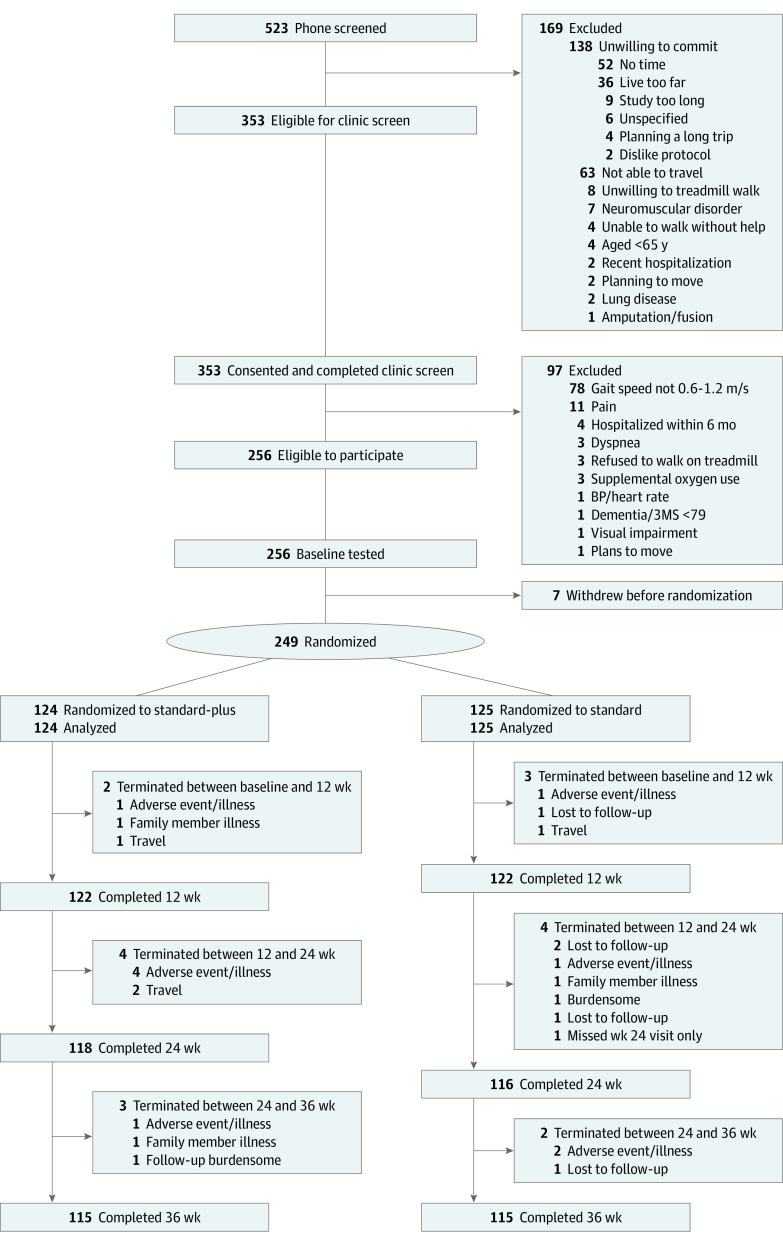
Screening, Randomization, and Follow-up of Participants in the Program to Improve Mobility in Aging (PRIMA) Trial 3MS indicates Modified Mini-Mental State examination; BP indicates blood pressure.

Individuals in the standard-plus group completed a median (IQR) of 21 (19-23) exercise sessions compared with 23 (19-24) in the standard group (*P* = .07). Excluding the 5 terminated participants, group attendance rates were 80.5% for standard-plus and 85.6% for standard. Among slower walkers, the median (IQR) for standard-plus was 21 (18.5-22.5) and 23 (20-24) for standard (*P* = .02); among faster walkers, the median (IQR) was 22 (19-23) for standard-plus and 22 (19-24) for standard (*P* = .70).

### Primary Outcome

In the standard-plus group, the mean (SD) gait speed improvement was 0.079 (0.135) m/s at 12 weeks, 0.065 (0.141) m/s at 24 weeks, and 0.059 (0.150) m/s at 36 weeks; in the standard group, gait speeds improved 0.081 (0.124) m/s at 12 weeks, 0.051 (0.129) m/s at 24 weeks, and 0.065 (0.148) m/s at 36 weeks. However, the 2 groups did not have significantly different improvements at each time point (Table 2). Among faster walkers, individuals in the standard-plus group demonstrated significant within-group improvements in mean (SD) gait speed at all follow-up time points (12 weeks: 0.074 [0.133] m/s, 24 weeks: 0.052 (0.130) m/s; and 36 weeks: 0.047 (0.143) m/s; all *P* < .05 by paired samples *t* test), whereas those in the standard intervention demonstrated improvements only immediately postintervention (12 weeks: 0.060 [0.116] m/s; *P* < .05).

**Table 2.** Intervention Effects on Primary Outcome of Gait Speed Change

Participant group and time frame	Standard-plus		Standard		Adjusted difference (SE), m/s^b^	*P* value^b^
	Gate speed change, mean (SD), m/s	*P* value^a^	Gate speed change, mean (SD), m/s	*P* value^a^		
All						
12 wk	0.079 (0.135)	<.001	0.081 (0.124)	<.001	0.001 (0.018)	.94
24 wk	0.065 (0.141)	<.001	0.051 (0.129)	<.001	0.001 (0.020)	.96
36 wk	0.059 (0.150)	<.001	0.065 (0.148)	<.001	−0.003 (0.019)	.88
Slow walkers (<1.0 m/s)						
12 wk	0.088 (0.140)	<.001	0.111 (0.131)	<.001	−0.016 (0.028)	.56
24 wk	0.086 (0.155)	<.001	0.087 (0.115)	<.001	−0.015 (0.030)	.61
36 wk	0.076 (0.159)	<.01	0.112 (0.138)	<.01	−0.020 (0.031)	.52
Fast walkers (≥1.0 m/s)						
12 wk	0.074 (0.133)	<.001	0.060 (0.116)	<.001	0.013 (0.024)	.58
24 wk	0.052 (0.130)	.002	0.027 (0.133)	.12	0.012 (0.025)	.64
36 wk	0.047 (0.143)	.02	0.028 (0.147)	.16	0.008 (0.0260)	.76

^a^ Obtained using paired samples *t* test.

^b^ Obtained using a linear mixed model with multiple imputation for missing values.

### Secondary and Tertiary Outcomes

The 2 intervention groups did not have significantly different improvements in any secondary outcomes at any time point (Table 3; eTable 3 and eTable 5 in Supplement 2). There were improvements in 6MWD, F8W time, and steps at all time points within each group (Table 3). In standard-plus, 12-week change in muscle power was significantly associated with change in SPPB (correlation coefficient, 0.25; *P* = .02). Among slower walkers, there were within-group improvements in 6MWD and F8W at all time points. Among faster walkers, the standard-plus group had improvements in 6MWD and F8W at all time points, whereas the standard group had improvements in 6MWD at 12 and 24 weeks and F8W at 12 weeks only (eTable 3 in Supplement 2).

**Table 3.** Intervention Effects on Secondary Outcomes (Representing Components of Intervention and Additional Mobility Measures)

Outcome and time frame	Standard-plus		Standard		Adjusted difference (SE)^a^	*P* value^a^
	Mean (SD)	*P* value	Mean (SD	*P* value		
Lower extremity strength change, newtons						
12 wk	7.5 (29.7)	.02	3.0 (23.6)	.21	−1.8 (4.5)	.68
24 wk	−2.2 (35.5)	.60	2.0 (27.9)	.54	−4.9 (4.6)	.30
36 wk	5.7 (26.0)	.10	−0.5 (24.8)	.89	−4.0 (4.8)	.41
LE power change, watts						
12 wk	−14.3 (55.7)	.54	−3.9 (61.4)	.02	−11.0 (10.2)	.28
24 wk	−11.3 (49.5)	.06	−6.0 (58.9)	.39	−11.8 (10.1)	.24
36 wk	0.6 (53.5)	.93	−12.3 (68.7)	.17	−13.0 (13.2)	.34
Six-minute walk distance change, m						
12 wk	39.9 (63.4)	<.001	35.0 (53.5)	<.001	4.1 (9.0)	.65
24 wk	31.8 (58.0)	<.001	20.6 (76.2)	.008	7.3 (10.2)	.47
36 wk	34.5 (56.8)	<.001	26.1 (72.8)	<.001	9.9 (9.7)	.31
Figure of 8 time change, s						
12 wk	−1.0 (1.6)	<.001	−1.0 (2.0)	<.001	−0.1 (0.2)	.58
24 wk	−0.8 (1.5)	<.001	−0.7 (2.2)	.002	−0.1 (0.2)	.51
36 wk	−0.7 (1.6)	<.001	−0.8 (2.2)	<.001	−0.0 (0.2)	.98
Figure of 8 steps change, steps						
12 wk	−1.000 (2.3)	<.001	−0.9 (2.3)	<.001	−0.2 (0.3)	.56
24 wk	−1.0 (2.1)	<.001	−0.4 (2.4)	.10	−0.4 (0.3)	.19
36 wk	−0.7 (1.9)	<.001	−0.7 (2.1)	.001	0.0 (0.3)	.95
Chair sit and reach change, cm						
12 wk	0.6 (7.5)	.42	2.4 (7.2)	<.001	−1.6 (1.0)	.12
24 wk	1.8 (7.7)	.02	1.6 (8.4)	.06	0.8 (1.2)	.48
36 wk	−0.8 (7.9)	.34	−0.1 (8.6)	.93	−0.2 (1.2)	.87
Modified Gait Efficacy Scale change						
12 wk	−1.7 (10.8)	.09	−0.2 (10.9)	.84	−1.5 (1.3)	.28
24 wk	−1.4 (9.9)	.13	−1.3 (9.5)	.13	−0.3 (1.3)	.81
36 wk	−2.3 (12.0)	.046	−1.6 (9.5)	.07	−0.9 (1.3)	.49
Short Physical Performance Battery change						
12 wk	0.2 (1.5)	.24	0.4 (1.3)	.002	−0.2 (0.2)	.28
24 wk	0.4 (1.4)	<.001	0.5 (1.3)	<.001	−0.1 (0.2)	.68
36 wk	0.2 (1.4)	.02	0.4 (1.3)	.002	−0.2 (0.2)	.36

^a^ Obtained using a linear mixed model with multiple imputation for missing values.

The 2 intervention groups had significantly different improvements in sedentary behavior over 12 weeks (adjusted difference [SE], −16.1 [8.1] min/d; *P* = .047; Table 4), primarily driven by faster walkers. Faster walkers in standard-plus had a greater decrease in sedentary behavior over 12 weeks (adjusted difference [SE], −26.9 [11.4] min/d; *P* = .02) (Table 4). There were no improvements in self-reported activity and participation in either group at any time point (eTable 4 in Supplement 2).

**Table 4.** Intervention Effects on Tertiary Outcomes of Physical Activity (Additionally Adjusted for Wear Time)

Group, outcome, and time frame	Standard-plus		Standard		Adjusted difference (SE)^a^	*P* value^a^
	Mean (SD)	*P* value	Mean (SD)	*P* value		
All participants						
Sedentary behavior change, min/d						
12 wk	−13.9 (59.2)	.049	4.2 (73.0)	.59	−16.1 (8.1)	.05
24 wk	−13.0 (62.2)	.09	6.1 (69.0)	.45	−11.9 (11.5)	.32
36 wk	1.7 (71.7)	.85	1.4 (58.9)	.85	−3.4 (10.3)	.75
Moderate to vigorous activity change, min/d						
12 wk	3.4 (15.9)	.07	0.1 (14.2)	.93	3.1 (2.8)	.28
24 wk	−3.5 (21.8)	.19	−2.9 (16.6)	.15	−0.1 (2.2)	.95
36 wk	−2.9 (17.4)	.19	−3.3 (13.6)	.048	0.9 (3.0)	.78
Steps change, steps/d						
12 wk	132 (1166)	.34	−80 (1216)	.54	208 (237)	.39
24 wk	−362 (1322)	.03	−423 (1532)	.02	52 (188)	.78
36 wk	−479 (1362)	.007	−489 (1098)	<.001	31 (182)	.86
Vector magnitude change, counts/d						
12 wk	9032 (66 505)	.25	−8313 (65 215)	.24	15 479 (11 663)	.19
24 wk	−24 578 (119 701)	.09	−16 740 (78 750)	.07	−3429 (11 223)	.76
36 wk	−27 259 (75 706)	.005	−20 978 (58 291)	.004	2401 (11 329)	.83
Slow walkers (<1.0 m/s)						
Sedentary behavior change, minutes/d						
12 wk	−7.6 (71.0)	.55	−7.5 (94.0)	.64	−5.4 (13.9)	.70
24 wk	−11.6 (59.7)	.30	0.5 (61.7)	.97	−7.1 (14.0)	.61
36 wk	−6.5 (62.7)	.57	−9.6 (62.5)	.43	−2.9 (16.8)	.87
Moderate to vigorous activity change, min/d						
12 wk	−1.3 (9.3)	.43	0.5 (14.5)	.83	−2.9 (3.8)	.45
24 wk	−7.0 (14.8)	.85	0.5 (13.4)	.02	−2.6 (3.8)	.49
36 wk	−2.6 (16.0)	.38	−2.8 (11.1)	.20	−0.8 (4.5)	.86
Steps change, steps/d						
12 wk	−5 (770)	.97	13 (1140)	.95	−157 (318)	.63
24 wk	−648 (1059)	.003	192 (1258)	.44	−394 (330)	.24
36 wk	−401 (1089)	.05	−258 (700)	.07	−179 (328)	.59
Vector magnitude change, counts/d						
12 wk	−1822 (36 668)	.78	−2020 (67 677)	.86	−12 219 (17 065)	.48
24 wk	−30 918 (67 780)	.02	8796 (68 864)	.51	−16 931 (18 509)	.37
36 wk	−21 406 (57 445)	.05	−11 911 (52 139)	.25	−9276 (18 981)	.63
Fast walkers (≥1.0 m/s)						
Sedentary behavior change, minutes/d						
12 wk	−18.7 (48.8)	.02	12.1 (54.2)	.11	−26.9 (11.4)	.02
24 wk	−14.1 (64.7)	.18	9.3 (73.3)	.39	−15.6 (15.2)	.32
36 wk	8.9 (79.0)	.52	8.8 (56.0)	.33	−3.7 (11.9)	.76
Moderate to vigorous activity change, minutes/d						
12 wk	7.0 (18.7)	.02	−0.1 (14.1)	.95	7.0 (3.4)	.05
24 wk	−0.9 (25.6)	.82	−4.7 (18.1)	.08	1.6 (3.0)	.60
36 wk	−3.1 (18.8)	.34	−3.7 (15.2)	.13	2.0 (3.6)	.60
Steps change, steps/d						
12 wk	235 (1394)	.29	−142 (1271)	.42	433 (305)	.18
24 wk	−155 (1461)	.51	−776 (1576)	.002	356 (293)	.24
36 wk	−548 (1578)	.051	−645 (1285)	.003	175 (254)	.50
Vector magnitude change, counts/d						
12 wk	17 239 (81 743)	.18	−12 549 (63 818)	.16	30 344 (16 361)	.08
24 wk	−19 981 (147 081)	.40	−31 409 (80 985)	.01	5043 (15 915)	.75
36 wk	−32 425 (89 349)	.04	−27 099 (61 988)	.009	9759 (15 407)	.53

^a^ Obtained using a linear mixed model with multiple imputation for missing values additionally adjusted for wear time.

The 2 intervention groups did not differ in any serious adverse events. Forty-three individuals in standard-plus experienced 62 events (18 serious events) and 41 individuals in standard experienced 57 events (15 serious events). All serious events were unrelated to the interventions.

## Discussion

This randomized clinical trial provides important evidence with respect to changes in mobility limitations and exercise. Both intervention groups experienced significant and clinically meaningful improvements in mobility immediately after the intervention (12 weeks). These within-group improvements in gait speed were sustained over time (24 and 36 weeks). Contrary to our hypothesis, however, the standard-plus timing and coordination exercise program did not result in greater improvements in mobility than the standard exercise program. Although it is imperative to document these findings to provide potential explanations and limit publication bias, it is also crucial to demonstrate the overall importance of exercise to improve mobility.

We used an active control based on our pilot trial with between-intervention differences, but both groups improved mobility with nonsignificant between-group differences. Another trial to improve mobility in older adults using active control demonstrated similar results.^7^ Likely, any is better than no exercise for this population; comparative effectiveness trials historically produce smaller effect sizes than inactive controls.^34^ Achieving meaningful between-group differences may require exercise at doses intolerable for older adults with mobility limitations.

The findings are inconsistent with our prior pilot studies. We previously demonstrated between-group differences in mobility outcomes despite use of active controls, emphasizing pilot study findings do not always persist in larger trials.^16,17,35^ A potential explanation is the mode of endurance training. The standard group’s endurance training consisted of walking on a treadmill to compare against the standard-plus intervention accurately, whereas in pilot studies we used stationary cycles for endurance training. Treadmill walking has been shown to reduce gait variability,^36^ most likely due to the external step generating action and facilitating consistent timing of locomotor pattern of steps.^37,38^ The treadmill walking characteristics we defined through setting initial speed and progressions bear similarities to the timing and coordination training of the standard-plus program, including specificity in the dosing of speed for accuracy in training repetitions, a defined goal for the walking and a knowledge of success for the participant. We may have blunted the differences between the intervention groups by using treadmills instead of stationary cycles for endurance training in the standard group.

Standard-plus elicited greater improvements in total physical activity (ie, vector magnitude) and decreases in sedentary behavior than the standard exercise program. Both intervention groups received the same behavioral physical activity intervention, but only the standard-plus group demonstrated improvements in physical activity. These differences were primarily driven by those with a faster baseline gait speed. The improvements in physical activity were not maintained over time, likely owing to a suboptimal plan to promote maintenance of activity.

Why did groups differ in physical activity but not mobility? The ultimate goal of the standard-plus intervention was to enhance the motor skill of walking for the older adult to make the older adult a motor expert in walking, defined as having efficient gait and tiring less quickly. Consequently, they may be likely to walk more, participate in more activities, experience less fatigue and report less disability. Yet we did not see significantly different between-group improvements in walking distance or participation in clinical measurements (ie, 6MWD and LLFDI). Alternatively, it is possible the standard-plus intervention affected adaptability of walking (ie, speed changes, path changes in straight to curves and direction of curves), which may be evidenced in an improved ability to maneuver comfortably in the home, the gym, or outdoors but may not be seen under controlled study conditions. The greater improvements in physical activity in standard-plus were more pronounced in faster walkers. Slower walkers did not have significant improvements in physical activity, possibly indicating they did not reach the level of motor expert in walking.

The findings differed between slower and faster walkers in other respects. Slower walkers in both groups improved mobility after the intervention and maintained them. We did not see improvements in physical activity among slower walkers. Among faster walkers, the standard-plus maintained within-group improvements in mobility over time whereas the standard improvements in mobility did not sustain at 24-weeks and 36-weeks postintervention. Faster walkers in standard-plus also had greater improvements in physical activity, possibly contributing to sustained mobility improvements.

It may be that older adults need a certain level of mobility to engage and gain benefits from the physical activity behavioral modification intervention. Slower walkers may experience benefits in mobility from building capacity (ie, conditioning) with either intervention, but once walking is at a certain threshold of ability (ie, ≥1.0 m/s) the added challenge of the timing and coordination of walking may improve the efficiency and motor skill of walking. Faster walkers may have an adequate level of capacity to tolerate the cost of their compensated walking^39^ and need the challenge of the timing and coordination component to become motor experts in walking. Differences in mobility and physical activity improvements with intervention by baseline walking speed supports consideration of a staged intervention approach to improve mobility. As typical for sports training and rehabilitation, building a foundation of conditioning precedes the sport-specific motor skill training and participation.^40^

### Limitations

Although this was a rigorously conducted trial with high adherence and retention (98% at 12 weeks), important limitations should be considered. Study participants are representative of the race and ethnicity of Pittsburgh but do not represent all older adults in the United States. Although our sample had impaired mobility, they had to be medically stable and able to participate in a walking exercise intervention to participate in the study, thus they likely do not represent all community-dwelling older adults. However, the sample did have multiple chronic conditions and more than one-third reported a fall in the previous year which is comparable to other community-dwelling older adults. Without a nonexercise control group, we cannot assure the improvements in mobility are related only to the exercise programs and not to the socialization and attention aspects. We were not comfortable withholding an active intervention from a group who could potentially benefit. Also, it is difficult to recruit participants to a study with a chance of not receiving any intervention. Other medical treatments during the study period were not collected. A nontrivial proportion of participants lacked accelerometry-monitored physical activity and were different from those that did in some respects. However, those with physical activity data had similar baseline characteristics between the 2 intervention groups. Finally, in keeping with the a priori analysis plan, multiplicity corrections were not applied for the many secondary and tertiary outcomes.

## Conclusions

This randomized clinical trial found that treatment with either a standard or a standard-plus intervention resulted in improved gait speed that was maintained for 24 weeks after the intervention; however, there was no difference between groups. Improving mobility, which is associated with lower incidence of future falls, is important to the health of older adults.^41^ Future research should more thoroughly examine the timing and sequencing of exercise interventions to improve walking in older adults and finding and targeting those most likely to benefit.

## Notes

Supplement 1.
Trial Protocol

Supplement 2. **eTable 1.** Inclusion and Exclusion Criteria **eTable 2.** Physical Activity Subsample: Participant Characteristics and Measures at Baseline: Mean ± Standard Deviation or N (%) **eTable 3.** Intervention Effects on Secondary Outcomes (Representing Components of Intervention and Additional Mobility Measures) Stratified by Screening Gait Speed **eTable 4.** Intervention Effects on Tertiary Outcomes (Self-Report Activity and Participation)

Supplement 3.
Data Sharing Statement

## References

[1] Perera S, Patel KV, Rosano C, . Gait speed predicts incident disability: a pooled analysis. J Gerontol A Biol Sci Med Sci. 2016;71(1):63-71. doi:10.1093/gerona/glv126 26297942 PMC4715231

[2] Studenski S, Perera S, Patel K, . Gait speed and survival in older adults. JAMA. 2011;305(1):50-58. doi:10.1001/jama.2010.1923 21205966 PMC3080184

[3] Hoffman JM, Ciol MA, Huynh M, Chan L. Estimating transition probabilities in mobility and total costs for Medicare beneficiaries. Arch Phys Med Rehabil. 2010;91(12):1849-1855. doi:10.1016/j.apmr.2010.08.010 21112425 PMC3404130

[4] Perera S, Mody SH, Woodman RC, Studenski SA. Meaningful change and responsiveness in common physical performance measures in older adults. J Am Geriatr Soc. 2006;54(5):743-749. doi:10.1111/j.1532-5415.2006.00701.x 16696738

[5] Van Abbema R, De Greef M, Crajé C, Krijnen W, Hobbelen H, Van Der Schans C. What type, or combination of exercise can improve preferred gait speed in older adults? a meta-analysis. BMC Geriatr. 2015;15:72. doi:10.1186/s12877-015-0061-9 26126532 PMC4488060

[6] Hortobágyi T, Lesinski M, Gäbler M, VanSwearingen JM, Malatesta D, Granacher U. Effects of three types of exercise interventions on healthy old adults’ gait speed: a systematic review and meta-analysis. Sports Med. 2015;45(12):1627-1643. doi:10.1007/s40279-015-0371-2 26286449 PMC4656792

[7] Bean JF, Kiely DK, LaRose S, O’Neill E, Goldstein R, Frontera WR. Increased velocity exercise specific to task training versus the National Institute on Aging’s strength training program: changes in limb power and mobility. J Gerontol A Biol Sci Med Sci. 2009;64(9):983-991. doi:10.1093/gerona/glp056 19414509 PMC2720885

[8] Zech A, Drey M, Freiberger E, . Residual effects of muscle strength and muscle power training and detraining on physical function in community-dwelling prefrail older adults: a randomized controlled trial. BMC Geriatr. 2012;12:68. doi:10.1186/1471-2318-12-68 23134737 PMC3538686

[9] Pahor M, Blair SN, Espeland M, ; LIFE Study Investigators. Effects of a physical activity intervention on measures of physical performance: Results of the lifestyle interventions and independence for Elders Pilot (LIFE-P) study. J Gerontol A Biol Sci Med Sci. 2006;61(11):1157-1165. doi:10.1093/gerona/61.11.1157 17167156

[10] Freiberger E, Häberle L, Spirduso WW, Zijlstra GAR. Long-term effects of three multicomponent exercise interventions on physical performance and fall-related psychological outcomes in community-dwelling older adults: a randomized controlled trial. J Am Geriatr Soc. 2012;60(3):437-446. doi:10.1111/j.1532-5415.2011.03859.x 22324753

[11] Chou CH, Hwang CL, Wu YT. Effect of exercise on physical function, daily living activities, and quality of life in the frail older adults: a meta-analysis. Arch Phys Med Rehabil. 2012;93(2):237-244. doi:10.1016/j.apmr.2011.08.042 22289232

[12] Pahor M, Guralnik JM, Ambrosius WT, ; LIFE study investigators. Effect of structured physical activity on prevention of major mobility disability in older adults: the LIFE study randomized clinical trial. JAMA. 2014;311(23):2387-2396. doi:10.1001/jama.2014.5616 24866862 PMC4266388

[13] Ferrucci L, Bandinelli S, Benvenuti E, . Subsystems contributing to the decline in ability to walk: bridging the gap between epidemiology and geriatric practice in the InCHIANTI study. J Am Geriatr Soc. 2000;48(12):1618-1625. doi:10.1111/j.1532-5415.2000.tb03873.x 11129752

[14] Keysor JJ. Does late-life physical activity or exercise prevent or minimize disablement? a critical review of the scientific evidence. Am J Prev Med. 2003;25(3)(suppl 2):129-136. doi:10.1016/S0749-3797(03)00176-4 14552936

[15] Brach JS, Lowry K, Perera S, . Improving motor control in walking: a randomized clinical trial in older adults with subclinical walking difficulty. Arch Phys Med Rehabil. 2015;96(3):388-394. doi:10.1016/j.apmr.2014.10.018 25448244 PMC4850731

[16] Brach JS, Van Swearingen JM, Perera S, Wert DM, Studenski S. Motor learning versus standard walking exercise in older adults with subclinical gait dysfunction: a randomized clinical trial. J Am Geriatr Soc. 2013;61(11):1879-1886. doi:10.1111/jgs.12506 24219189 PMC3827693

[17] VanSwearingen JM, Perera S, Brach JS, Cham R, Rosano C, Studenski SA. A randomized trial of two forms of therapeutic activity to improve walking: effect on the energy cost of walking. J Gerontol A Biol Sci Med Sci. 2009;64(11):1190-1198. doi:10.1093/gerona/glp098 19643842 PMC2981453

[18] Brach JS, VanSwearingen JM, Gil A, . Program to improve mobility in aging (PRIMA) study: methods and rationale of a task-oriented motor learning exercise program. Contemp Clin Trials. 2020;89:105912. doi:10.1016/j.cct.2019.105912 31838258 PMC6945812

[19] Kramer MK, McWilliams JR, Chen HY, Siminerio LM. A community-based diabetes prevention program: evaluation of the group lifestyle balance program delivered by diabetes educators. Diabetes Educ. 2011;37(5):659-668. doi:10.1177/0145721711411930 21918204

[20] Borg G. Perceived exertion as an indicator of somatic stress. Scand J Rehabil Med. 1970;2(2):92-98. 5523831

[21] Brach JS, VanSwearingen JM. Interventions to improve walking in older adults. Curr Transl Geriatr Exp Gerontol Rep. 2013;2(4):230-238. doi:10.1007/s13670-013-0059-0 24319641 PMC3851025

[22] Brach JS, Perera S, Studenski S, Newman AB. The reliability and validity of measures of gait variability in community-dwelling older adults. Arch Phys Med Rehabil. 2008;89(12):2293-2296. doi:10.1016/j.apmr.2008.06.010 19061741 PMC2705958

[23] Mangione KK, Craik RL, McCormick AA, . Detectable changes in physical performance measures in elderly African Americans. Phys Ther. 2010;90(6):921-927. doi:10.2522/ptj.20090363 20395305

[24] Callahan D, Phillips E, Carabello R, Frontera WR, Fielding RA. Assessment of lower extremity muscle power in functionally-limited elders. Aging Clin Exp Res. 2007;19(3):194-199. doi:10.1007/BF03324689 17607086

[25] Butland RJ, Pang J, Gross ER, Woodcock AA, Geddes DM. Two-, six-, and 12-minute walking tests in respiratory disease. Br Med J (Clin Res Ed). 1982;284(6329):1607-1608. doi:10.1136/bmj.284.6329.1607 6805625 PMC1498516

[26] Jones CJ, Rikli RE, Max J, Noffal G. The reliability and validity of a chair sit-and-reach test as a measure of hamstring flexibility in older adults. Res Q Exerc Sport. 1998;69(4):338-343. doi:10.1080/02701367.1998.10607708 9864752

[27] Hess RJ, Brach JS, Piva SR, VanSwearingen JM. Walking skill can be assessed in older adults: validity of the Figure-of-8 Walk Test. Phys Ther. 2010;90(1):89-99. doi:10.2522/ptj.20080121 19959654 PMC2802825

[28] Guralnik JM, Simonsick EM, Ferrucci L, . A short physical performance battery assessing lower extremity function: association with self-reported disability and prediction of mortality and nursing home admission. J Gerontol. 1994;49(2):M85-M94. doi:10.1093/geronj/49.2.M85 8126356

[29] Newell AM, VanSwearingen JM, Hile E, Brach JS. The modified Gait Efficacy Scale: establishing the psychometric properties in older adults. Phys Ther. 2012;92(2):318-328. doi:10.2522/ptj.20110053 22074940 PMC3269773

[30] World Health Organization. International Classification of Functioning, Disability and Health. 1905 5/22/1 AD. Accessed April 22, 2021. https://www.who.int/standards/classifications/international-classification-of-functioning-disability-and-health

[31] Haley SM, Jette AM, Coster WJ, . Late life function and disability instrument: II. development and evaluation of the function component. J Gerontol A Biol Sci Med Sci. 2002;57(4):M217-M222. doi:10.1093/gerona/57.4.M217 11909886

[32] Jette AM, Haley SM, Coster WJ, . Late life function and disability instrument: I. development and evaluation of the disability component. J Gerontol A Biol Sci Med Sci. 2002;57(4):M209-M216. doi:10.1093/gerona/57.4.M209 11909885

[33] Rubin D. Multiple Imputation for Nonresponse in Surveys. John Wiley and Sons; 1987.

[34] Williams CM, Skinner EH, James AM, Cook JL, McPhail SM, Haines TP. Comparative effectiveness research for the clinician researcher: a framework for making a methodological design choice. Trials. 2016;17(1):406. doi:10.1186/s13063-016-1535-6 27530915 PMC4988047

[35] VanSwearingen JM, Perera S, Brach JS, Wert D, Studenski SA. Impact of exercise to improve gait efficiency on activity and participation in older adults with mobility limitations: a randomized controlled trial. Phys Ther. 2011;91(12):1740-1751. doi:10.2522/ptj.20100391 22003158 PMC3229041

[36] Hollman JH, Watkins MK, Imhoff AC, Braun CE, Akervik KA, Ness DK. A comparison of variability in spatiotemporal gait parameters between treadmill and overground walking conditions. Gait Posture. 2016;43:204-209. doi:10.1016/j.gaitpost.2015.09.024 26481257

[37] Warabi T, Kato M, Kiriyama K, Yoshida T, Kobayashi N. Treadmill walking and overground walking of human subjects compared by recording sole-floor reaction force. Neurosci Res. 2005;53(3):343-348. doi:10.1016/j.neures.2005.08.005 16182398

[38] Capaday C. The special nature of human walking and its neural control. Trends Neurosci. 2002;25(7):370-376. doi:10.1016/S0166-2236(02)02173-2 12079766

[39] VanSwearingen JM, Studenski SA. Aging, motor skill, and the energy cost of walking: implications for the prevention and treatment of mobility decline in older persons. J Gerontol A Biol Sci Med Sci. 2014;69(11):1429-1436. doi:10.1093/gerona/glu153 25182600 PMC4271095

[40] Handford C, Davids K, Bennett S, Button C. Skill acquisition in sport: some applications of an evolving practice ecology. J Sports Sci. 1997;15(6):621-640. doi:10.1080/026404197367056 9486439

[41] Shuman V, Coyle PC, Perera S, Van Swearingen JM, Albert SM, Brach JS. Association between improved mobility and distal health outcomes. J Gerontol A Biol Sci Med Sci. 2020;75(12):2412-2417. doi:10.1093/gerona/glaa086 32270185 PMC7662180

