# Supplementary material for: Effect of Timing and Coordination Training on Mobility and Physical Activity Among Community-Dwelling Older Adults: A Randomized Clinical Trial
Source: JAMA Netw Open. 2022 May 23;5(5):e2212921. doi: 10.1001/jamanetworkopen.2022.12921 (PMC9127558; doi:10.1001/jamanetworkopen.2022.12921)
Supplement: Supplement 1. — Trial Protocol [file jamanetwopen-e2212921-s001.pdf]

This supplement contains the following items:

1) Original protocol, final protocol, and summary of changes
2) Original statistical analysis plan, final statistical analysis plan, and summary of changes

TASK SPECIFIC TIMING AND COORDINATION EXERCISES TO IMPROVE MOBILITY IN OLDER ADULTS

(PRIMA: Program to Improve Mobility in Aging)

Principal Investigator:

Jennifer S. Brach, PhD, PT

Supported by:

The National Institute on Aging

AG045252

TABLE OF CONTENTS

Page

Task Specific timing and coordination exercises to improve mobility in older adults i

TABLE OF CONTENTS ii

PRÉCIS v

STUDY TEAM ROSTER 1

Principal Investigator: 1

Co-Investigators: 1

1 Study objectives 2

1.1 Primary Objective 2

1.2 Secondary Objectives 2

2 BACKGROUND AND RATIONALE 2

2.1 Exercise for Health Promotion: Have we forgotten the nervous system? 3

2.2 Why would the standard-plus intervention impact activity and participation? 3

2.3 Why would the benefits of the standard-plus intervention be sustained after the intervention has ended? 4

2.4 Study Rationale 4

3 STUDY DESIGN 6

3.1 Overview and Study Design 6

4 SELECTION AND ENROLLMENT OF PARTICIPANTS 7

4.1 Inclusion Criteria 7

4.2 Exclusion Criteria 7

4.3 Study Enrollment Procedures 8

4.3.1 Identifying and Recruiting Participants 8

5 STUDY INTERVENTIONS 9

5.1 Overview 9

5.2 Blinding and Study Staff 9

5.3 Group Lifestyle Balance^{TM} (GLB) - Behavioral Intervention 9

5.4 Standard Intervention (include progression and modifications) ... 10
5.5 Standard-Plus Intervention (include progression and modifications) ... 10
5.6 Treatment Fidelity Plan ... 11
5.6.1 Initial Training for Procedural Reliability ... 11
5.6.2 Ensuring Ongoing Competency ... 11
5.7 Scheduling Intervention Visits ... 12
5.8 Adherence Assessment ... 13

6 STUDY PROCEDURES ... 13

6.1 Schedule of Evaluations ... 14
6.2 Description of Evaluations ... 16
6.2.1 Screening (Phone and Clinic) ... 16
6.2.2 Baseline ... 17
6.2.3 Follow-up Visits ... 21

7 SAFETY ASSESSMENTS ... 22

7.1 Participant Safety Parameters: Methods and Timing ... 22
7.1.1 Screening ... 22
7.1.2 Safety Considerations for Assessments and Interventions ... 22
8. Adverse Events and Serious Adverse Events ... 24
8.6 Follow-up for Adverse Events ... 28

9. INTERVENTION DISCONTINUATION ... 29

10 Data Analyses ... 30

10.1 Main Analysis. ... 31
10.2 Exploratory Analyses. ... 31
10.3 Compliance and Dropout Analyses. ... 31
10.4 Interim Analysis ... 32
10.5 Sample Size Adequacy ... 32
10.5.1 Primary Outcome ... 32
10.5.2 Secondary and Tertiary Outcomes ... 33
10.6 Treatment Assignment Procedures - Randomization ... 33

11 DATA COLLECTION AND QUALITY ASSURANCE ... 34

11.1 Data Collection Forms ... 34
11.2 Data Management ... 34

11.3 Quality Assurance 35

12 PARTICIPANT RIGHTS AND CONFIDENTIALITY 36

12.1 Institutional Review Board (IRB) Review 36
12.2 Informed Consent Forms 36
12.3 Participant Confidentiality 36
12.4 Study Discontinuation 36

13 PUBLICATION OF RESEARCH FINDINGS 37

14 Ancillary Study Policies 37

15 REFERENCES 38

PRÉCIS

Study Title
Task Specific Timing and Coordination Exercise to Improve Mobility in Older Adults

Objectives. The primary objective of the proposed project is to evaluate the impact of adding timing and coordination training to standard strength and endurance training on mobility. Secondary objectives include examining 1) additional outcomes representing the components of the intervention and measures of activity and participation, 2) the delayed and sustained effects of the intervention, and 3) the effects of the intervention within various other subgroups of interest.

Design and Outcomes. This is a 5 year randomized single-blind two arm intervention trial to compare the effects on mobility, activity and participation of a standard strength, endurance, and flexibility program to the standard plus timing and coordination program in 248 community-dwelling older adults walking slower than the desired gait speed of 1.2 m/s. Exercise sessions are twice weekly for 12 weeks. Participants are assessed at baseline, 12 weeks (post intervention), 24 weeks and 36 weeks. The primary, secondary, and tertiary outcomes are conducted by assessors masked to group assignment. The primary outcome is gait speed. Secondary outcomes of maximum voluntary isometric contraction of the quadriceps femoris, Six minute walk test, Chair Sit-and-Reach Test, Smoothness of walking, and Gait variability represent the components of the intervention (i.e. strength, endurance, flexibility and timing and coordination). Tertiary outcomes represent measures of activity (Late Life Function and Disability Instrument – function subscale and confidence in walking) and participation (Late Life Function and Disability Instrument – disability subscale and daily physical activity).

Interventions and Duration. Intervention sessions are twice weekly for 12 weeks supervised by a physical therapist. All sessions last about 50-65 minutes and include a warm-up, 40-55 minutes of exercise, and a cool down period. Both intervention groups will also receive physical activity behavioral change intervention. The Standard Intervention includes a brief warm-up period, lower extremity strength training, endurance training, and a brief cool down period. The Standard-Plus Task Specific Timing and Coordination Intervention or Standard-Plus intervention includes all components of the Standard Intervention plus task specific timing and coordination training. The total intervention time will be equal between the two intervention groups.

Sample Size and Population. The target sample is community-dwelling older adults who walk faster than 0.60 m/s and slower than the desired gait speed of 1.2 m/s. We plan to enroll 124 subjects who walk slowly (i.e. gait speed > 0.60 and < 1.0 m/s) and 124 faster walkers (i.e. gait speed ≥ 1.0 and < 1.20 m/s) for a total of 248 subjects. Within each baseline gait speed stratum we plan to randomize individuals to each of the treatment arms so that the gait speed strata are equally represented in each of the arms. Persons who are unable to participate in testing, have medical conditions which would make testing or participation in an exercise program unsafe, or who have plans to permanently leave the area during the study are excluded.

STUDY TEAM ROSTER

Principal Investigator:

Jennifer S. Brach, PhD, PT
Bridgeside Point 1
100 Technology Drive
Pittsburgh, PA 15219-3130
Phone: 412-383-6533
Fax: 412-648-5970
jbrach@pitt.edu
Main responsibilities/Key roles: Oversees and is responsible for all aspects of the study

Co-Investigators:

**Rakie Cham, PhD**<br>Department of Bioengineering<br>302 Benedum Hall<br>Pittsburgh, PA<br>(412) 624-7227<br>rcham@pitt.edu<br>Main responsibilities: Gait outcomes	**Andrea Kriska, PhD**<br>Department of Epidemiology<br>505B Public Health<br>Pittsburgh, PA<br>(412) 624-3996<br>aky@pitt.edu<br>Main responsibilities: GLB behavioral intervention
**Neelesh Nadkarni, MD, PhD**<br>Division of Geriatric Medicine<br>Kaufmann Building, Suite 500<br>Pittsburgh, PA<br>Phone: (412) 692-2383<br>nkn3@pitt.edu<br>Main responsibilities: Study physician	**Subashan Perera, PhD**<br>Division of Geriatric Medicine<br>Kaufmann Building, Suite 500<br>Pittsburgh, PA<br>Phone: (412) 692-2365<br>Ksp9@pitt.edu<br>Main responsibilities: Randomization, data management and study statistician
**Jessie VanSwearingen, PhD, PT**<br>Bridgeside Point 1<br>100 Technology Drive<br>Pittsburgh, PA 15219-3130<br>Phone: 412-383-6533<br>jessievs@pitt.edu<br>Main responsibilities/Key roles: Quality control of the intervention	

1 STUDY OBJECTIVES

1.1 Primary Objective

To determine if a standard-plus program is more successful in improving walking ability (i.e. gait speed) at 12 weeks after study entry compared to a standard exercise program. *Compared to participants receiving the standard program, participants receiving the standard-plus program will have greater gains in gait speed at 12 weeks after study entry (Hypothesis 1.1); and differences in gains between the programs will occur in both slower (<1.0 m/s)and faster (≥1.0 m/s) walkers (Hypothesis 1.2).*

1.2 Secondary Objectives

To assess the effect of the interventions on secondary and tertiary outcomes that represent the main components of the intervention and measures of activity and participation. *Compared to participants receiving the standard program, participants receiving the standard-plus program will have greater gains in timing and coordination (smoothness of walking, gait variability), activity (Late Life Function and Disability Instrument - function), and participation (Late Life Function and Disability Instrument-disability and physical activity as measured by Actigraph accelerometer) at 12 weeks after study entry; gains in strength (leg press strength and power), endurance (Six Minute Walk Test) and flexibility (Chair Sit-and-Reach) will be similar between the two active treatment groups.*

To determine if walking ability, secondary, and tertiary outcomes differ between standard-plus and standard intervention groups after a delay and are sustained over a period of time. *Compared to the participants receiving the standard program, participants receiving the standard-plus program will have greater gains at 24 (delayed effect) and 36 weeks (sustained effect) post randomization.*

To explore the effects of the interventions on outcomes within various other subgroups of interest (i.e. baseline confidence, physical activity and cognition subgroups).

2 BACKGROUND AND RATIONALE

Walking difficulty in older adults contributes to loss of independence, higher rates of morbidity and increased mortality.(1-5) Mobility loss is also a sentinel predictor of other disabilities that restrict independent living.(6) Compared to older adults without self-reported walking difficulty, those who developed mild walking difficulty over one year had higher healthcare costs (mean $1,128 per person). Extrapolated to the estimated 22% of older adults who develop walking difficulty annually, the cost to society is an additional 3.6 billion dollars per year.(7) Therefore, preventing or delaying the onset of walking difficulty might have a substantial impact on quality of life and healthcare costs of older adults.

2.1 Exercise for Health Promotion: Have we forgotten the nervous system?

Current exercise recommendations for older adults from the American College of Sports Medicine and the American Heart Association focus primarily on strength, endurance and flexibility training.(8, 9) Based on these exercise recommendations, the ongoing Lifestyle Interventions and Independence for Elders (LIFE) study examines a standard walking endurance, strength, static balance and flexibility intervention.(10) The LIFE pilot study, using the same intervention, demonstrated significant but *modest* effects.(11) For example, walking speed improved by only 0.01 m/s in the exercise group and decreased 0.02 m/s in the control group. The key question is: have we addressed all the most important contributors to walking ability? Might we be missing something?

Walking is a complex task that places demands on multiple systems including the musculoskeletal (muscles, bones, and joints), cardiopulmonary (heart and lungs), and nervous systems (brain, spinal cord and peripheral nerves).(12, 13) Current exercise recommendations target the musculoskeletal and cardiopulmonary systems and overlook the nervous system. A motor skill based exercise approach uses task specific timing and coordination exercises to challenge the brain to adapt and learn the sequence of movements and timing with the postures and phases of gait to improve walking. Improvements in walking occur by restoring the pattern of brain and neuromuscular activation that optimizes the use of capacities to meet the demands of the task of walking. Given the widespread subclinical and clinical neurological abnormalities with aging, it is time to incorporate explicit neurological training into exercise for older adults. Thus a potentially important but as yet unaddressed strategy to promote walking ability through exercise is to add training of timing and coordination in gait (i.e. task specific timing and coordination training) to usual strength, endurance, and flexibility training. Our preliminary data, presented below, suggests that task specific timing and coordination training has beneficial effects on mobility (gait speed) that are greater than a standard strength and endurance program.

2.2 Why would the standard-plus intervention impact activity and participation?

Walking underlies many activities of daily living and walking difficulty is associated with reduced activity and participation.(5, 14) The timing and coordination component of the standard-plus intervention, is a <u>task-oriented</u> approach which emphasizes the sequence of movements and timing with the postures and phases of gait to improve walking. The ultimate goal of the timing and coordination training is to make the older adult a skilled, “expert” walker. For motor tasks, expert movers, or those with greater motor skill for a specific activity, tire less easily than novices because of the greater efficiency of skilled motor performance.(15-17) Older adults who are “expert walkers” (i.e. skilled walkers) have efficient gait, tire less easily and as a result will likely walk more, participate in more activities and report less disability.(18)

2.3 Why would the benefits of the standard-plus intervention be sustained after the intervention has ended?

Intervention strategies challenge the brain to improve walking performance in different ways. An impairment-based intervention, such as the standard strength and endurance program, challenges the brain to use increased capacity in body systems to compensate for gait difficulties. Walking performance likely improves secondary to increased ability to produce muscle forces, to move joints through a greater range of motion, and to deliver more oxygenated blood to the active tissues. The use of greater capacity of body systems for walking makes the outcome of the impairment-based intervention approach potentially inefficient and difficult to sustain. A motor skill based approach challenges the brain to adapt and learn the sequence of movements and timing with the postures and phases of gait to improve walking. Improvements in walking occur by restoring the pattern of brain and neuromuscular activation that optimizes the use of capacities to meet the demands of the task of walking. The task-oriented focus of the motor skill based approach has the potential to lead to not only an efficient and automatic motor sequence pattern for walking, but also reward-based adaptive changes in the brain which may be sustainable. Recently, in a randomized controlled trial comparing strength exercises to task-oriented exercises in older women, improvements in functional task performance were sustained for six months after the end of training only in the task-oriented group.(19)

2.4 Study Rationale

Rationale for Interventions

Standard impairment-based strength and endurance interventions, which ignore the timing and coordination of movement, have a real but modest impact on walking ability in older adults.(10, 20-25) Aging and disease alter timing and coordination as reflected by slowed neuromotor performance, increased gait variability and reduced smoothness of movement.(26-29) A task specific timing and coordination intervention that includes practice of smooth coordinated aspects of gait timing over multiple walking conditions has the potential to improve walking ability greater than a standard program.(30)

We conducted two pilot studies, involving contrasting subject groups, to examine the short-term impact of a timing and coordination exercise program on walking. The first study (RESTORE) included older adults with slow (gait speed < 1.0 m/s) and variable gait and has been published.(30) The second study (PRIME) has recently been completed.(31) It included older adults with near normal gait speed (gait speed > 1.0 m/s) but with difficulty with aspects of the timing and coordination of walking (i.e. Figure of 8 test time > 8.0 see measures section below for details of this measure). Participant retention for the 12 week posttests was over 95%.

In the RESTORE study, 50 subjects (mean age 77.2±5.5 years, 65% women) were randomly assigned to either a standard exercise program (endurance, strength and typical static balance training) or a timing and coordination program, for one hour, 2

times per week for 12 weeks, with baseline and 12 week follow up assessments. Of the 50 who entered, 47 (94%) completed the study. Both groups increased gait speed (timing and coordination by 0.21 m/s and standard by 0.14 m/s); adjusted group difference 0.07 m/s, p=0.10. The timing and coordination group had a 3.5±1.7 (p=0.04) point greater gain than the standard group in basic lower extremity function (LLFDI) and a 2.6±1.7 (p=0.12) point greater gain than the standard group in advanced LE function (LLFDI).(32) Note that this pilot targets a population similar to that proposed here but that the treatment arms differ from the current proposal in that the timing and coordination group did not receive endurance training.

In the PRIME study, 38 subjects (mean age 78.5±5.6 years, 65% women) were randomly assigned to either a standard endurance and strength exercise program or a timing and coordination plus strengthening program, 2 times per week for 12 weeks, with assessments at baseline and immediately following the 12 week intervention. Preliminary analyses indicate that the timing and coordination group had greater improvements in gait speed than the standard group (adjusted group difference 0.11 m/s, p=0.008). Both groups had improvements in endurance (6MWT: p<0.05) and the timing and coordination group had greater improvements in the timing and coordination of walking (Smoothness of walking HR_{AP} adjusted group difference = 0.53, p=0.05 and Figure of 8 test time adjusted group difference = -1.39s, p<0.0001). The timing and coordination group reported increased participation (LLFDI-disability limitation increased 6.0 points, p=0.05; however, neither group improved on activity measures (LLFDI–function) most likely due to the high baseline values and potential ceiling effect. The percent of time spent in sedentary behavior did not differ significantly between the groups immediately following the intervention (adjusted group difference -1.7%, p=0.27). We hypothesize that changes in daily activity will likely be delayed, in that subjects need time to adjust to their improved walking prior to increasing their daily activity.(31, 33)

Figure 1 presents the changes in gait speed achieved in the LIFE-P study, and our two

Figure1. Changes in gait speed with standard and timing and coordination (T&C) training.

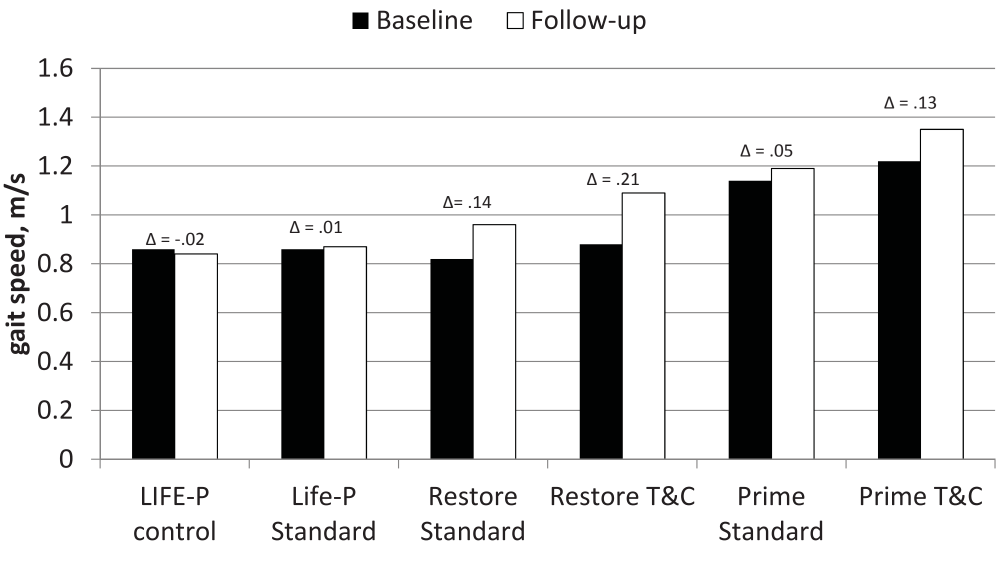

pilot studies (RESTORE and PRIME). Improvements in gait speed were modest in LIFE-P and the standard program groups of RESTORE and PRIME and much larger in the timing and coordination groups. Note improvements in gait speed with timing and coordination training were evident in both slower (RESTORE) and faster (PRIME) walkers. Though the overall improvements in gait speed with timing and coordination training were greater for the slower walkers in RESTORE, adjusted group differences in gait speed were larger for the faster walkers in PRIME (PRIME group difference = 0.11m/s, SE=0.04; p=0.008 and RESTORE group difference = 0.07m/s, SE=0.04; p=0.10). Individuals with gait speed > 1.0 m/s did not benefit as much from the standard intervention compared to the task specific timing and coordination training (i.e. gait speed increased 0.05 m/s for the standard training and 0.13 m/s for the task specific timing and coordination training), suggesting that task specific timing and coordination may play a critical role in improving mobility in older adults with near normal gait speed (i.e. > 1.0 m/s).

3 STUDY DESIGN

3.1 Overview and Study Design

A randomized single-blind two arm intervention trial of 248 community-dwelling older adults walking slower than the desired gait speed of 1.2 m/s will be carried out at the University of Pittsburgh. Half of the participants will receive the standard strength, endurance, and flexibility program (Standard) and the other half will receive the standard plus timing and coordination program (Standard plus). Both groups will receive 16 sessions of behavioral interventions to promote physical activity. Potential participants will be screened over the phone to determine initial eligibility and then will be scheduled for a clinical screening visit. Prior to the clinic screening visit, the participant's physician will be contacted and clearance to participate in a moderate intensity exercise program will be obtained. At the clinic screening visit, written informed consent will be obtained and the screening examination will occur to determine final eligibility. Eligible participants (i.e. those who meet all inclusion and exclusion

Figure 1. Timeline for recruitment, randomization, and follow-up.

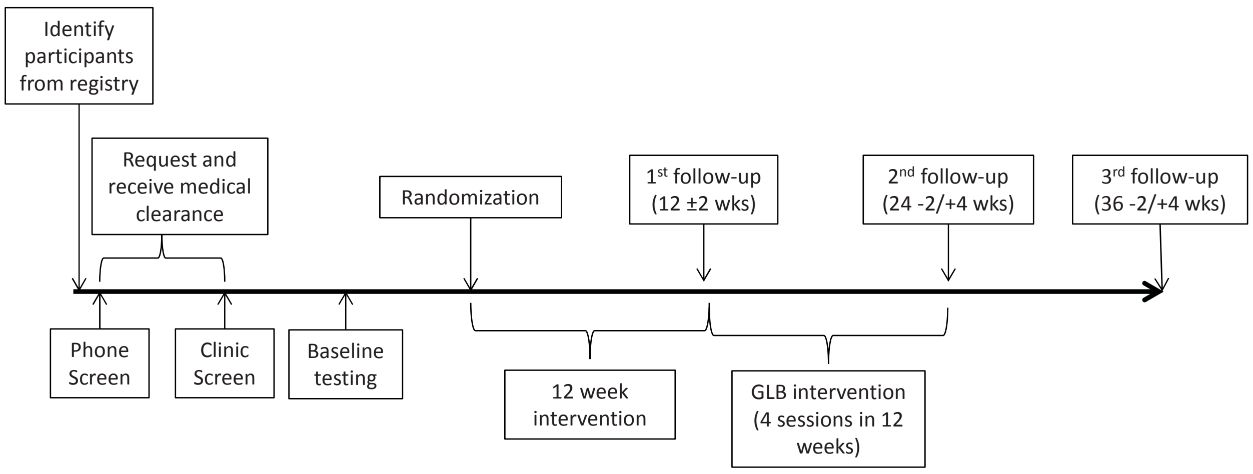

criteria) will undergo a comprehensive baseline assessment and will then be randomized to one of the two treatment groups. Assessments will be collected again at 12 weeks (post intervention), 24 weeks and 36 weeks post randomization. Figure 1 shows the sequence of participant contacts from the time of initial contact to the final assessment approximately 36 weeks after randomization.

4 SELECTION AND ENROLLMENT OF PARTICIPANTS

Candidates will be recruited through the Pittsburgh Pepper Center Registry. Screening will be conducted in 2 phases (phone and clinic). Initial phone contact uses a structured questionnaire to identify inclusion and exclusion criteria by self-report. During the phone screen, we will obtain the name and phone number of the participant's primary care physician in order to obtain medical clearance for participation in the exercise intervention. Medical clearance for participation in the exercise intervention must be obtained prior to the onsite clinical examination. Individuals who meet criteria on the phone screen will be scheduled for an onsite clinic screen to identify additional potential exclusions. Prior to initiating the onsite examination, informed consent will be obtained. To determine eligibility based on gait speed, gait speed will be measured using a timed 4 meter corridor walk. We plan to enroll 124 subjects who walk slowly (i.e. gait speed > 0.60 and < 1.0 m/s) and 124 faster walkers (i.e. gait speed ≥ 1.0 and < 1.20 m/s) for a total of 248 subjects. The phone and clinic screening are described in greater detail in section 6.2.1 below.

4.1 Inclusion Criteria

Participants must meet all of the following inclusion criteria to participate in this study. Inclusion criteria include the following:

1. 65 years of age and older
2. Ambulatory without an assistive device or the assistance of another person
3. Usual 4 meter gait speed > 0.60 m/s and < 1.2 m/s
4. Physician clearance to participate in a moderate intensity exercise program
5. Not meeting physical activity recommendations defined as reporting less than 150 minutes of moderate intensity activity per week in the past month.[7]

4.2 Exclusion Criteria

All candidates meeting any of the exclusion criteria at time of screening will be excluded from study participation. Exclusion criteria include the following:

1. Inability to participate in testing or exercise intervention:
    a. Persistent lower extremity pain that is present on most days of the week that would interfere with participation in the exercise program.

b. back pain that is present on most days of the weeks and interferes with walking and activities of daily living or back pain that increases with walking (lumbar stenosis)
c. calf pain or cramping which worsens with walking and is relieved by rest (PAD)
d. refuse to walk on a treadmill
e. plans to move out of the area in the next 5 years

2. Safety concerns:
    a. dyspnea at rest or during activities of daily living or use supplemental oxygen (CHF, COPD)
    b. any acute illness or medical condition that is not stable according to the approving physician
    c. resting systolic blood pressure ≥ 200 mm Hg or diastolic blood pressure ≥ 100 mm Hg or resting heart rate > 100 or < 40 beats per minute[71]
    d. diagnosed dementia or cognitive impairment defined as 3MS<79
    e. hospitalized in the past 6 months for acute illness or surgery, other than minor surgical procedures
    f. severe visual impairment as indicated by difficulty navigating in the clinic
    g. fixed or fused lower extremity joints such as hip, knee or ankle
    h. lower extremity strength <3/5 on manual muscle testing
    i. lower extremity amputation
    j. progressive movement disorder such as MS, ALS or Parkinson's disease

4.3 Study Enrollment Procedures

4.3.1 Identifying and Recruiting Participants

Candidates will be recruited through the Pittsburgh Pepper Center Registry of over 2200 older adults who have signed consents to be directly contacted by participating researchers about mobility research studies. This sample is diverse in gender, age and ethnicity and has a wide range of self-reported mobility. Potential participants from the registry will be sent a letter and asked to contact the study coordinator by phone. If the potential participant does not respond within 2 weeks we will contact them by phone and ask if they would like to hear more about the study.

During the initial phone call, potential participants will be informed of the nature of the screening questions. They will also be informed that their answers to the questions will be stored confidentially. Additionally, they will be reminded that their participation in the phone screening is strictly voluntary. The participant's verbal consent will be noted on the telephone screening form. If the potential participant declines the telephone interview, the form will be destroyed. If the after the phone interview the potential participant is ineligible, the reason for ineligibility will be documented on the screening

log. If after the phone interview the participant is eligible we will obtain the name and phone number for the primary care physician so that medical clearance can be obtained. They will then be scheduled for a clinic screen visit and notified of the date, time and location of the visit. Written informed consent will be obtained prior to conducting the clinic screen. Details of the clinic screen, baseline testing and randomization can be found below in section 6.2.

5 STUDY INTERVENTIONS

5.1 Overview

Participants will be assigned to one of two treatment groups: 1) Standard or 2) Standard-Plus. The Standard intervention group will receive strength, endurance and flexibility training and the Standard-Plus intervention group will receive strength, endurance, flexibility and timing and coordination training. Both groups will receive 24 sessions approximately 60 minutes in duration from a study PT. Participants will receive 2 sessions per week on non-consecutive days for 12 weeks. Training sessions will occur at the Physical Therapy Clinical and Translational Research Center (PT-CTRC).

5.2 Blinding and Study Staff

Given the nature of the interventions being tested in this study, it will not be possible to blind the participants or the PT providing the interventions. To minimize “contamination” of the interventions, participants receiving different interventions will not be scheduled during the same time at the PT-CTRC. Study staff involved in the randomization or intervention will not perform follow-up data collection. Study staff performing the follow-up assessments will be blinded to treatment assignments and will be asked not to seek this information when in contact with the participants. Participants will be asked not to discuss their treatment experience during study visits. Only a small number of staff members and members of the DSMB will see group-specific study results.

5.3 Group Lifestyle Balance^{TM} (GLB) - Behavioral Intervention

Both intervention groups will also receive a physical activity behavioral change intervention based on the Group Lifestyle Balance^{TM} (GLB) program. The GLB program is a behavioral lifestyle intervention which is modeled closely on the original and highly successful Diabetes Prevention Program (DPP). The DPP is a NIH funded study that demonstrated that small changes in lifestyle such as healthy eating and increased physical activity could lower the risk of developing type 2 diabetes in those at high risk for the disease. The GLB was designed to help individuals reach a balance between two parts of their lifestyle, eating and physical activity.(34)

We will primarily focus on the physical activity component of the program. The physical activity goal of the GLB program is for participants to achieve and maintain a physical activity level of at least 150 minutes each week of moderate intensity activity similar to a

brisk walk. Participants in the Standard and Standard-plus interventions will participate in 16 GLB sessions. The first 12 sessions will be weekly, individual sessions. The final 4 sessions will be delivered in a small group setting over a 12 week time period as follows: every other week for 4 weeks (2 sessions), once every 4 weeks for 8 weeks (2 sessions). Sessions cover topics such as “Jump Start Your Activity Plan”, “Problem Solving”, “Ways to Stay Motivated”, “Stress and Time Management”, etc. All sessions are thoroughly described in the behavioral intervention manual.

5.4 Standard Intervention (include progression and modifications)

The Standard Intervention includes a brief warm-up period (5 minutes), lower extremity strength training (10-15 minutes), endurance training (30-40 minutes), and a brief cool down period (5 minutes) for a total of 50 to 65 minutes of intervention. The warm-up and cool down periods contain gentle stretches for the lower extremity and trunk. The strength training is conducted on Magnum stacked weight training equipment (leg extension/ curl combo, leg press machine, and multi-hip combo) and can include the following exercises: knee extension, knee flexion, leg press, hip abduction, hip extension, etc. When subjects are able to complete 2 sets of 15 repetitions with minimal effort (i.e. RPE < 10), resistance is increased for progression of the exercises. Subjects complete exercises for at least 3-4 lower extremity muscle groups which are selected by the physical therapist based on the individual participant needs. The endurance training consists of treadmill walking at a submaximal workload with a self-reported rating of perceived exertion (RPE) of 10-13, somewhat hard. When subjects are able to tolerate a 10-13 RPE level for 15 minutes, the workload is increased. The goal is to achieve 40 minutes of continuous treadmill walking exercise at a somewhat hard level of exertion. For safety, participants are told they should stop walking immediately if they feel they cannot continue (symptom-limited), if they or the PT observes shortness of breath, if they demonstrate problems in the walking pattern (e.g. toe drags on the floor during the swing-through phase of gait), or they report or the physical therapist observes any of the general indications for stopping nondiagnostic exercise tests as recommended by ACSM (e.g. ACSM Exercise Guidelines, Table 4-6, p78).(35) A complete description of the standard program, including progression of exercises, can be found in the MOP.

5.5 Standard-Plus Intervention (include progression and modifications)

In addition to the Standard Intervention described above the subjects receiving the Standard-Plus Intervention will complete task specific timing and coordination training. In order to keep the total intervention time equal and the time spent in walking/standing activities equal, the subjects in the Standard Plus intervention complete a 5 minute warm-up, 10-15 minutes of lower extremity strength training, 15-20 minutes of endurance training, 15-20 minutes of timing and coordination training and a 5 minute cool-down for a total of 50-65 minutes of intervention. The timing and coordination training is based on principles of motor learning that enhance “skill” or smooth and automatic movement control.(16, 36-40) This program uses goal-oriented progressively more difficult stepping and walking patterns to promote the timing and coordination of

stepping, integrated with the phases of the gait cycle.(16, 37, 38, 40) Conceptually, the exercise is intended to achieve its effects by shifting the center of pressure posteriolateral then forward, encouraging hip extension prior to stepping, loading the trailing limb, coordinating activation of the abductors of the soon to be swing leg with adductors of the stance limb, and shifting the center of pressure in medial stance to unload the stepping limb.(41-43) Progression is based on first separately increasing the speed, amplitude or accuracy of performance prior to undertaking a more complex task.(44) For example, the progression of stepping patterns is, 1) self-paced step forward and across, 2) increase stepping speed, 3) alternate side of stepping, 4) alternate forward with backward stepping. Walking patterns incorporate patterns of muscle coordination and interlimb timing similar to the stepping exercises into walking. Walking patterns progress by separately altering speed, amplitude (e.g. narrowing oval width), or accuracy of performance (e.g. without straying from the desired path), and then to complex walking patterns involving walking past others and with upper extremity object manipulation tasks added, such as carrying, bouncing or tossing a ball.(40) Timing and coordination training was used in both of our pilot studies.(30, 31) The standard impairment-based intervention aims to increase physiologic capacity in body systems that contribute to walking, but does not include task specific exercise necessary to make use of the physiological capacity in body systems (i.e. musculoskeletal and cardiopulmonary systems) for the walking. Whereas, the task specific timing and coordination intervention aims to improve the motor skill of walking by re-aligning biomechanical and neuromotor programs, and improving feedback for adjusting movements. In a sense, an impairment-based intervention is comparable to "building a bigger engine" whereas the task specific timing and coordination intervention is comparable to a "tune-up" of an engine for optimal performance.(45) A complete description of the timing and coordination program, including progression of exercises, can be found in the MOP.

5.6 Treatment Fidelity Plan

5.6.1 Initial Training for Procedural Reliability

Dr. VanSwearingen and Dr. Brach will train the PTs in both the Standard and the Standard-plus interventions. Knowledge of procedures will be tested by psychomotor skills observation. Dr. VanSwearingen and Dr. Brach will document that the PTs are "PRIMA" certified PTs and provide a certificate of completion after they demonstrate competence with the interventions. All PT-CTRC PTs are CPR certified and must maintain this certification throughout the study. As new PTs join the study, training will be conducted on an individualized basis following the same procedures.

5.6.2 Ensuring Ongoing Competency

We will use a multi-faceted approach to ensure ongoing treatment fidelity. The approaches include direct observation of skills, mandatory monthly telephone calls with all treating PTs, periodic review of intervention data sheets, and face-to-face meetings with the intervention team at least once per year.

<u>Direct Observation of Skills</u>: Direct observation of the PTs as they perform the interventions will be conducted by Dr. VanSwearingen or a senior research physical

therapist trained in the intervention. The evaluators will document their observations using a structured checklist that addresses data completeness, physical performance, qualitative observations, and verbal and nonverbal communication. In the Standard intervention the checklist will also document whether the participant completed 5 minutes of warm-up, 10-15 minutes of lower extremity strength training consisting of exercises that address at least 3 muscle groups, 30-40 minutes total of endurance training at an RPE of 10-13, and a 5 minute cool down. In the Standard-plus intervention the checklist will also document whether the participant completed 5 minutes of warm-up, 10-15 minutes of lower extremity strength training consisting of exercises that address at least 3 muscle groups, 15-20 minutes of endurance training at an RPE of 10-13, 15-20 minutes of timing and coordination training and a 5 minute cool down. There will be two observations per quarter for each intervention for new PTs and one observation for each intervention per quarter for PTs who have been with the study more than 6 months. If the total score on the checklist is less than 90% a remediation plan will be developed that will include refresher training to ensure adequate understanding of the protocol and follow-up observation visits.

<u>Mandatory monthly telephone calls</u>: Dr. VanSwearinegen will conduct monthly telephone calls with the PTs delivering the intervention. The study coordinator will take minutes of the telephone meetings and document ongoing training. During these calls, the PTs will be able to ask questions about the intervention delivery. The calls will provide the PTs with an opportunity to brainstorm and problem solve with Dr. VanSwearingen.

<u>Periodic Review of Intervention data sheets</u>: Intervention data sheets will be reviewed periodically to ensure the proper intervention is being delivered and that the intervention is being progressed over time. Review of intervention data sheets will occur at the completion of the on-site intervention. Initially, intervention data sheets from two of the first 5 participants in each intervention arm will be reviewed. Once the initial reviews are completed, subsequent reviews will occur quarterly (i.e. two participants in each intervention). If the documentation of the intervention and/or the progression of the intervention is inadequate, a remediation plan will be developed that will include refresher training to ensure adequate understanding of the protocol and follow-up observation visits.

<u>Face-to-face meetings</u>: Dr. VanSwearingen will visit the PT-CTRC semi-annually to meet with the PTs delivering the interventions. This will be a team building and problem solving visit emphasizing proper delivery of the intervention and discussing issues specific to the site.

5.7 Scheduling Intervention Visits

There are 3 requirements for scheduling of intervention visits:

1. The first intervention visit should be scheduled within 2 weeks of randomization.

2. The general schedule of visits includes 2 visits per week for 12 weeks for a total of 24 visits. The goal is to complete 24 visits. Missed visits because of illness, holidays, or vacations can be rescheduled in the following weeks; however, all visits must occur within a 14 week time period.

3. Intervention visits should never occur on two consecutive days.

5.8 Adherence Assessment

Adherence to the intervention will be defined as the number of intervention sessions completed. Adherence to the exercise intervention (standard or standard-plus) will be tracked separately from the adherence to the behavioral intervention. The staff delivering the intervention will track and record the attendance of the participant in the exercise intervention and the behavioral intervention on the adherence log form. At the completion of the intervention, the final total attendance will be entered into the database.

6 STUDY PROCEDURES

6.1 Schedule of Evaluations

Assessment	Phone Screen	Clinic Screen	Baseline	12 week	24 week	36 week	Each intervention visit	As needed
Phone Screen	X							
Informed Consent Form		X						
Demographics Questionnaire		X						
Physical Exam		X						
Comorbidity Index		X						
Anthropometric Measures		X		X	X	X		
Gait speed screen		X						
Modified Mini-Mental State (3MS) Test		X						
Medical clearance to participate			X					
Fall history questionnaire			X	X	X	X		
Gait Assessment Walkway			X	X	X	X		
Leg strength and power			X	X	X	X		
Six minute walk test			X	X	X	X		
Chair sit and reach			X	X	X	X		
Smoothness of walking			X	X	X	X		
Gait Efficiency			X	X	X	X		
Figure of 8			X	X	X	X		
Gait Efficacy Scale			X	X	X	X		

CHAMPS questionnaire			X	X	X	X		
Late Life Function and Disability Index			X	X	X	X		
Geriatric Depression Scale			X	X	X	X		
Actigraph Diary			X	X	X	X		
Exercise adherence log							X	
Adverse events								X
Change in status								X
Health status update								X
Medical clearance to return after illness or event								X
Protocol deviation								X

6.2 Description of Evaluations

6.2.1 Screening (Phone and Clinic)

Screening will be conducted in two phases, phone and clinic. Initial phone contact uses a structured questionnaire to identify inclusion and exclusion by self-report. Individuals who meet the criteria will be scheduled for an onsite examination (clinic screen) to identify additional potential exclusions.

Consenting Procedure

Prior to the phone screen we will obtain verbal consent. During the initial telephone call, potential participants will be informed of the nature of the screening questions. They will also be informed that their answers to the questions will be stored confidentially. Additionally, they will be reminded that participation in the telephone screening is voluntary. The participant's verbal consent will be noted on the telephone screening form. If the potential participant declines the telephone interview, the form will be destroyed.

Medical Clearance. Once the participant passes the phone screen, we will obtain the name and phone number of the participant's primary care physician so that we can obtain medical clearance for participation in the study. The physician's office will be contacted and the fax number obtained. We will fax the physician's office a letter and a form for medical clearance and ask that it be completed and returned to us by mail or fax. Medical clearance must be obtained prior to the clinic visit for screening and baseline testing.

Prior to performing any of the clinic screen measures, written informed consent will be obtained. During the clinic screen visit, the participant will be given the consent form to read. The consent form will then be reviewed in the presence of one of the study investigators. The investigator will then answer any questions that the participant may have about the study. The investigator will ask the participant several questions about the study to make sure they understand the study procedures. Finally the participant will be asked to sign the consent form. The signed consent form will be stored in the participant's research record in a locked file cabinet. The participant will be given a copy of the consent form for their records.

Clinic Screen

The clinic screen will include the following measures:

- Demographic questionnaire
- Physical Exam
- Comorbidity Index
- Anthropometirc measurements
- Gait speed – 4 meter walk

- Modified Mini-Mental State (3MS) test

All screening measures will be completed prior to determining subject eligibility. Once all measures are collected, the values will be reviewed to determine eligibility. Participants will be informed at the clinic screen visit of their eligibility. Participants who are not eligible will terminate participation at the end of the clinic screen. Participants who are eligible will continue on with baseline testing. All screening tests must be measured within 14 days of randomization.

Enrollment
This study utilizes a single consent form that describes both screening and study procedures. In this study enrollment is defined as the randomization date.

6.2.2 Baseline

Baseline assessment will occur immediately following the clinic screen and will include the following measures:

Mobility Outcome (Primary Outcome):
Gait Speed - The primary mobility outcome is gait speed. We selected gait speed as our primary outcome because of its strong psychometric properties, its significant association with morbidity and mortality, and the continuous nature of the variable. Gait speed is a reliable,(46, 47) valid, sensitive(11) and specific(48) measure that correlates with functional ability and balance confidence. Gait speed is strongly association with future disability, falls, hospitalization, nursing home admission and mortality.(1-5) Gait speed is a responsive measure that can assess change over time(11, 49) thus an improvement in gait speed is likely a good indicator of the effectiveness of an exercise program. In addition, gait speed was chosen by a panel of experts as the standardized assessment to measure locomotion for the Motor Function Domain of the NIH Toolbox.

Gait speed is assessed in usual walking with a computerized walkway. After explanation and demonstration, the participant completes a practice walk the length of the walkway to become accustomed to walking on the mat. Each walk is considered one pass. The subject then completes 6 passes on the walkway at their usual, self-selected walking speed. Gait speed will be averaged over the 4 passes. The test-retest reliability of gait speed measured using a computerized walkway by ICC is 0.98.(50) A higher speed is better.

Outcomes representing components of the interventions (Secondary Outcomes):

In order to assess the integrity of the intervention, we selected measures that represent the main components of the intervention (i.e. strength, endurance, flexibility, and timing and coordination) as our secondary outcomes. Measures representing the underlying components of the interventions are:

Lower Extremity Strength and Power - Leg strength will be measured by determining the 1 repetition maximum (1RM) for each leg individually using a Keiser A420 electronic

pneumatic leg press machine. We will follow the protocol previously described by Callahan et al, 2007.(51) The maximum value observed on the A420 graphical display of either side will be recorded as the peak leg strength. Leg press power will also be measured using the Keiser A420 leg press machine. Leg press power will be measured as the peak power graphically recorded when the individual performs a single leg press repetition pushing out as quickly as possible at 40% and 70% of 1RM. Five repetitions will be recorded for each leg at each resistance. The highest recorded power of all repetitions (either side, either resistance) will be recorded as leg press power.

Six Minute Walk Test (6MWT) - The Six Minute Walk Test (6MWT) of distance walked (meters) in six minutes, including time for rest as needed,(52) will be used to determine the impact of the exercise interventions on endurance. The 6MWT has been used to describe and monitor endurance capacity.(52) The 6MWT has established psychometric properties, test-retest reliability (Pearson r=.95) in older adults,(48, 53) construct validity for graded exercise test and functional classification.(54) We chose the 6MWT because it is responsive to change,(55) widely used, and included in the NIH, PROMIS project to establish measures for clinical assessment. Greater distance covered during six minutes is better.

Chair Sit-and-Reach Test - The Chair Sit-and-Reach Test, a measure of hamstring flexibility, will be used as the main measure of flexibility.(56) Subjects sit on the edge of a chair with their preferred leg extended and the other leg bent with the foot flat on the floor. Subjects bend forward at the hips, keeping the spine as straight as possible. The distance reached in relation to the foot is recorded in cm. The Chair Sit-and Reach test was selected as the measure of flexibility because it does not require the older adult to sit on the floor, which may be difficult for some. The test has established psychometric properties of test-retest reliability (ICC=0.92-0.96) and criterion validity with comparison to the sit-and-reach test (r=0.74). Values can be positive or negative with positive numbers indicating greater flexibility.

Smoothness of walking – Smoothness of Walking is an integrated measure of motor control of walking,(57, 58) and is assessed during usual over ground walking. Linear acceleration of the body is measured along three axes (vertical, anterior-posterior and medial-lateral) using a tri-axial accelerometer attached to the skin over the L3 segment of the lumbar spine. Trunk accelerations are sampled at 200 Hz and are used to calculate the harmonic ratio following the methodology of Menz.(57) The harmonic ratio (HR) is derived in the vertical, medial-lateral and anterior-posterior directions. Higher HRs are better.

Gait variability – Gait variability, defined as fluctuations in gait characteristics from one step to the next,(59) is an important indicator of impaired mobility in older adults and will serve as a second indicator of timing and coordination.(60) Gait variability is quantified using established measures of temporal and spatial gait characteristics including stance time, step length, and step width. Variability will be calculated as the standard deviation of the set of steps recorded over 4 passes on the GaitMat (described above). Approximately 32 steps will be collected from 4 passes on the GaitMat which will be more than adequate to achieve a stable measure of gait variability. Our prior work has shown that 20 steps are sufficient to achieve a reliability of 0.75 and 30 are sufficient for 0.80.(61) In general, lower variability is better although there are exceptions.(60, 62)

Gait Efficiency - We will use the energy cost of walking as our indicator of gait efficiency. The energy cost of walking reflects the energy used for all bodily actions during walking.(63) Subjects walk on a treadmill at a self-selected pace while oxygen consumption data is collected using open circuit spirometry and analysis of expired gases with a VO2000 portable metabolic measurement system, Medgraphics®, Minneapolis, MN. All subjects are familiarized with treadmill walking until comfortable walking on the treadmill, prior to the baseline measurement. The mean rate of oxygen and carbon dioxide consumption is determined over three minutes after reaching steady state.(63, 64) The energy cost of walking reported in ml/kg/m, represents an estimate of energy expenditure per unit of gait speed,(65-67) and relates to metabolic equivalents (METS). It is time independent, repeatable, reflects the physiological cost of gait,(63, 64) is little influenced by fitness,(63) and can be compared across individuals and over time, regardless of changes in gait speed.(63, 65) Lower cost is better.

Activity Outcomes (Tertiary Outcomes):
Using the World Health Organization's International Classification of Functioning, Disability and Health (ICF)(68) model to inform our selection of tertiary outcomes for this study, we will evaluate measures of activity (execution of a physical task).

Activity levels will be assessed with:
The Late Life Function and Disability Instrument (LLFDI-F).(14, 69) The LLFDI-F is our main activity outcome. The LLFDI-F is a self-report measure for assessing physical function in older adults with acute or chronic problems, and designed to be more sensitive to change than similar measures. The LLFDI-F has 32 items in three dimensions, basic lower extremity (BLE), advance lower extremity (ALE) and upper extremity (UE). We've selected the LLFDI-F because 1) it includes a wide variety of life tasks in various social areas thus extending beyond the traditional focus of just activities of daily living, 2) the scale was designed with sufficient breadth of items and increments of rating in order to minimize ceiling and floor effects and maximize the scale's ability to detect change over time, and 3) it is a continuous outcome which gives us greater power than a dichotomous outcome to detect change over time. We will focus our analyses on the LLFDI-F dimension scores (i.e. BLE function, ALE function, UE function). The LLFDI-F has established known groups validity and the test-retest reliability is extremely high for the function component (ICCs range from 0.91-0.98 for the dimensions). Scores range from 0-100; higher scores represent less difficulty.

Participation Outcomes (Tertiary):
Using the ICF(68) model to inform our selection of disability outcomes for this study, we will evaluate measures of participation (involvement in life situations). Participation levels will be assessed with:

The Late Life Function and Disability Instrument (LLFDI-D).(69) The LLFDI-D is our main participation outcome. The LLFDI-D is a self-report measure for assessing disability in older adults with acute or chronic problems, and designed to be more sensitive to change than similar measures. The LLFDI-D component has 16 items representing two dimensions, frequency of performance and limitation in performance of

life tasks. We will focus our analyses on the LLFDI-D dimension scores (i.e. disability frequency and disability limitation). We will also examine the disability domain scores (social role, personal role, instrumental role and management role) since they may provide insight into the impact of the disability on frequency of performance and perceived limitations.(69) The LLFDI-D scales have established known groups validity and the test-retest reliability is moderate to high (ICCs range from 0.68 to 0.82). Scores range from 0-100; higher scores represent less disability.

The Actigraph Accelerometer will be used to assess the participants' usual daily physical activity. The ActiGraph is often used in physical activity research and has recently been shown to be more reliable than other devices.(70) Accelerometers are electronic sensors that measure the quantity and intensity of ambulatory movement.(71) Due to the small size and weight of the monitor, the participant is not expected to experience a disruption in their gait or balance. The Actigraph Accelerometer will be worn on the waist during waking hours for 7 consecutive days, and the participants will be instructed to remove the monitor only for sleeping, during imaging studies, and or during swimming and bathing activities. For pre-intervention physical activity assessment the subject will be given the accelerometer during the baseline assessment. They will be asked to return the accelerometer during their first intervention visit. For post-intervention testing, the subject will receive the accelerometer during the last intervention visit and will be asked to return it during the post-intervention assessment. For the 24 and 36 week follow-ups the participant will be given the accelerometer during their visit and they will be asked to return it by mail in a postage paid envelope. We used the same methodology (returning by mail) in a past study of 120 older adults and received accelerometers back from > 95% of the subjects. The continuous data that are available through actigraphy provide many possible measures for analysis. We focus on 1) total activity counts, 2) percentage of time spent in sedentary activity and 3) number of bouts of moderate intensity activity as our outcomes.

Potential covariates

Trail Making Parts A&B – The Trail Making Test is a widely used test of executive cognitive function(72) that involves multiple cognitive domains and is administered in two parts. Completion of the Trail Making Test A (TMT-A) involves complex visual scanning, motor speed, and agility as participants draw lines to connect consecutively numbered circles as quickly as possible. Completion of Trail Making Test B (TMT-B) requires the additional processes of cognitive flexibility and set shifting as participants connect circles in an alternating sequence of numbers and letters, linking them in ascending order as fast as possible. Time to complete and number of errors for each portion will be recorded. Lower scores (faster times) indicate better performance.

Geriatric Depression Scale (GDS)(73) – The Geriatric Depression Scale has been used extensively in community-dwelling healthy and medically ill older adults to screen for depression. The short form GDS consists of 15 yes or no questions. Scores of 0-4 are considered normal, scores of 5-8 indicate mild depression, scores of 9-11 indicate moderate depression and scores of 12-15 indicate severe depression.

Pittsburgh Sleep Quality Index(74) – The Pittsburgh Sleep Quality Index is a self-rated questionnaire which assess sleep quality and disturbances over a 1-month time period. Nineteen individual items generate seven “component” scores: subjective sleep quality, sleep latency, sleep duration, habitual sleep efficiency, sleep disturbances, use of sleeping medication and daytime dysfunction. The sum of the scores for the seven components yields a global score.

Life Space Mobility(75) – The University of Alabama at Birmingham (UAB) Study of Aging Life-Space Assessment (LSA), measures a person’s usual pattern of mobility in the previous month. Life-space can be visualized as a pattern of areas defined by distance extending from the location where a person sleeps. The LSA allows for a range of mobility assessment from limited to the room where the person sleeps with assistance from another person to independently traveling out of town. The LSA documents mobility based on how far and how often a person travels and the amount of assistance that is needed. Scores range from 0 (totally bed-bound) to 120 (travelled out of town every day without assistance). Higher scores indicate a greater mobility.

Gait Efficacy Scale. In order to determine if changes in walking difficulty are associated with changes in confidence in walking, confidence will be assessed using the Gait Efficacy Scale.(76) The items include a range of gait activities such as walking over different surfaces, up and down curbs, and negotiating stairs. Each item has a 10 point Likert scale scoring option, with the total score for the 10 items, ranging from 0-100. A higher score represents greater confidence.

6.2.3 Follow-up Visits

Follow-up visits will occur at 12 (conclusion of the intervention), 24 and 36 weeks post randomization. Windows for follow-up testing are as follows: 12±2weeks, 24 -2/+4 weeks, and 36 -2/+4 weeks. Measurements will include the following:

12, 24, and 36 weeks:

- Gait Speed
- Lower extremity strength and power
- Six minute walk test
- Chair sit and reach test
- Smoothness of walking
- Gait Efficiency
- Figure of 8 walk test
- Gait Efficacy Scale
- CHAMPS
- LLFDI
- Physical Activity – Actigraph
- Trail Making Parts A&B

- Geriatric Depression Scale
- Pittsburgh Sleep Quality Index

7 SAFETY ASSESSMENTS

7.1 Participant Safety Parameters: Methods and Timing

7.1.1 Screening

Potential participants will be excluded during the screening phases if they have persistent pain (lower extremity or back) that would interfere with participation or if they are medically unstable or have medical conditions which may impact their safety (see exclusion criteria). Individuals with resting systolic blood pressure ≥200 mm Hg or diastolic blood pressure ≥100 mm Hg or resting hear rate > 100 or < 40 beats per minute will also be excluded. All participants must also have physician clearance to participate in a moderate intensity exercise program prior to the start of the exercise intervention.

7.1.2 Safety Considerations for Assessments and Interventions

Expected Adverse Experiences

1. Major risks such as a cardiac event or a fall are expected to be rare – expected to occur in less than 1% of people (less than 1 out of 100 people). Gardner et al, 2000,(77) reviewed controlled clinical trials of exercise interventions for older adults at-risk for falling. No cardiac events or falls were reported in the 12 clinical trials reviewed. The at-risk older adults in the studies reviewed have slightly poorer physical performance than the older persons we expect to recruit for our study. In our recent MOBILE study, a 1 year cohort study of 120 older adults participating in 3 clinic visits over a 1 year period there were no cardiac events or falls reported. In all conditions of testing in which the participant is standing and/or walking (eg conditions with a potential risk for falling), the participant will be directly supervised by the physical therapist. The physical therapist is present for all testing sessions. We expect this level of supervision reduces the risk of falling to an even greater degree.
2. Less severe risks of participation such as such as muscle soreness, fatigue, or minor sprains or strains with each assessment, are expected to be infrequent – expected to occur in 1-10% of people (1-10 out of 100 people). Gardner et al, 2000,(77) reviewed controlled clinical trials of exercise interventions for older adults at-risk for falling, finding reports of such side effects of the intervention reported in only 4 of the 12 studies reviewed. The side effects were not a reason for dropout from the study and were described as soreness or musculoskeletal symptoms, but no injuries.
3. There is a rare risk that confidentiality could be breached. All of the research records will be kept in a locked file cabinet and/or password protected files. All of the investigators and staff that assist with the management of the files are trained in the privacy and confidentiality regulations that govern research.

Minimizing Risk during Assessments and Interventions.

All assessments approved in this study are considered to be a part of everyday clinical practice. We have minimized risks we believe by applying usual safeguards for the physical therapy assessment of gait. Assessment side effects, such as muscle soreness, fatigue, or minor sprains or strains with each assessment, will be recorded by the physical therapist and monitored by Dr. Brach, the Principal Investigators in consultation with the physician investigator, Dr. Nadkarni. Based upon the existing literature and our own clinical experience, we anticipate the frequency of these side effects to be extremely low.(77)

We will maximize the safety of our subjects with the following procedures.

1. Individuals with absolute contraindications to testing will be excluded based on the inclusion/exclusion criteria.
2. Testing will be carefully monitored by a licensed physical therapist and will be adjusted according to the American College of Sports Medicine Guidelines for Exercise Testing and Prescription.(35)
3. In all conditions of testing in which the participant is standing and/or walking (eg conditions with a potential risk for falling), the participant will be directly supervised by the physical therapist.
4. Subjects will be required to get medical clearance from their primary care physician prior to initiating the exercise intervention.

Confidentiality.

Participant's confidentiality will be protected in the data collection process. All personnel involved with the research have read and signed a Confidentiality statement, and approval is being obtained from the University of Pittsburgh Biomedical Institutional Review Board. Consent forms and data collection forms that identify the participant by name will be stored in a locked cabinet. All computers are password protected. If the data are used in scholarly presentations or journal articles, the investigators will protect the anonymity of individual participants and will report only aggregate data (eg group means) where appropriate. The Principal Investigator will review data confidentiality processes monthly or as indicated with the project staff (Study coordinator and Physical Therapists). The Investigators are all certified in Research Practice Fundamentals, Human Subjects Research Module**.**

Management of Adverse Experiences

All assessments and exercise interventions will be delivered in the Physical Therapy Clinical and Translational Research Center (PT-CTRC). The emergency procedures are posted within the PT-CTRC (see attached) along with an AED. Emergency procedures are reviewed with the staff every 6 months. All PT-CTRC staff are certified in the American Heart Association BLS for Healthcare Providers (CPR and AED) program. Blood pressure and heart rate will be routinely monitored before and after the exercise interventions. Any symptoms that occur during activities performed by the participant as

instructed by the study intervention protocol will be monitored and acted upon. Cardiac symptoms will include the following: chest pain, shortness of breath, dizziness, syncope, lightheadedness, arm pain or numbness, acute onset fatigue/exhaustion, palpitation or fluttering in the chest, sweating and feeling of heart racing, acute onset severe headache, weakness or numbness in on one side, part or whole body, speech difficulties and fall to a lower level or floor. Cardiac signs will include the following: extremes of blood pressure or heart rate abnormality, wheezing, leg cramps or claudication, confusion or an acute change in participants' mental status, unresponsiveness or sluggish responses, fever, change in color of skin. Some of these activities may occur on the physical premises of the study site. If cardiac symptoms or signs arise during the exercise component of the intervention or in the physical premises, the staff will immediately terminate the exercise ensuring that the participant is seated or lying supine. Research staff will closely monitor the participant and if a medical emergency should occur, staff will immediately follow BLS procedures and call 911. They will describe the incident to the 911 operator and provide their location (Bridgeside Point I, Suite 470,100 Technology Drive, Pittsburgh PA 15219). Once the emergency situation is under control, the staff will contact the study coordinator and PI to notify them of the situation. If the symptoms are not serious enough to warrant CPR or to call 911 they will follow the procedures outlined in the manual of operations.

Participant Education about Potential Risks

Potential risks associated with study-related activities and interventions will be explained to each participant by trained study personnel during the informed consent process. Each participant will be instructed to report the occurrence of an AE to appropriate study staff at scheduled data collection times, to PTs administering the intervention, or spontaneously at any other time. Participants also will be encouraged to report concerns about the safety of participating in the study to any research staff.

8. Adverse Events and Serious Adverse Events

8.1 Overview

Expected adverse events (AEs) will be captured through interviews at 12, 24 and 36 weeks, based on the Health Status Update questionnaire. Reportable events are those events that have potential implications for participant safety and require individual reporting. Reportable AEs are defined as serious adverse events (SAEs), AEs that have potential implications for participant safety, unexpected AEs, and injury that occurs while a participant is under the supervision of study related personnel. These RAEs will require individual event reporting as described in section 8.4. The timely and complete account of RAEs will be a critical requirement for the protection of human subjects in this trial.

The DSMB will review and approve study-defined expected AEs and will be involved in the regular monitoring of the RAE reporting system. Reporting of expected AEs to the DSMB, the NIA, and the IRB will be through the database. RAEs will be monitored and tracked separately and reviewed by the DSMB and the NIA as outlined in section 8.4.

8.2 Classifying Adverse Events

An AE is any unfavorable or unintended medical occurrence in a human study participant that has taken place during the course of a research project, including any abnormal sign, symptom, or disease, whether or not related to participation in the research.

For the purposes of this study, any event that meets the criteria for an SAE, is unexpected, or results in injury to the participant while he/she is under the supervision of study related personnel will be classified as an RAE. Adequate review, assessment, and monitoring of RAEs require they be classified as to severity, expectedness, and potential relatedness to the study intervention.

8.2.1 Severity
The following guidelines will be used to determine level of severity:

Mild: Awareness of signs and symptoms, but easily tolerated and causing no loss of time from normal activities. No specific medical attention is required.

Moderate: Discomfort enough to cause a low level of inconvenience or concern to the participant and may interfere with daily activities. Symptoms may require minimal, local or noninvasive medical intervention.

Severe: Events interrupt the participant's normal daily activities and are usually incapacitating. Significant symptoms may require hospitalization or invasive medical intervention.

Life-threatening/Disabling: Events that may involve acute, life-threatening metabolic or cardiovascular complications (such as circulatory failure, hemorrhage, sepsis) or life – threatening physiological consequences. Intensive care or emergent invasive procedure is required.

Death: Causing death.

Severity is not synonymous with seriousness. A severe headache is not necessarily an RAE. However, mild chest pain may results in a day's hospitalization and thus would be classified as a RAE.

8.2.2 Expectedness
AEs will be assigned as to whether they were expected or unexpected based on current knowledge. Categories are defined as follows:

<u>Expected:</u> An AE that is anticipated on the basis of prior experience with the intervention under investigation; an event that can be attributed to the underlying condition of the participant being studied; or an event that can be attributed to the patient population being studied (see section--- Expected AEs). Expected AEs are captured in a standardized way by study personnel.

Unexpected: An AE that was not anticipated on the basis of prior experience with the underlying intervention under investigation; an event that can be attributed to the underlying condition of the participant being studied; or to the patient population being studied or an expected event whose frequency or severity exceeds what is anticipated. Unexpected events are reportable.

8.2.3 Relatedness

The PI in consultation with the Co-Investigators and an independent safety monitor will determine the degree to which RAEs are related to study procedures using the criteria below.

Definitely related: The adverse event is clearly related to the investigational procedure – i.e., an event that follows a reasonable temporal sequence from administration of the study intervention, follows a known or expected pattern of response to the study intervention, that is confirmed by improvement on stopping and reappearance of the event on repeated exposure, and that could not be reasonably explained by the known characteristics of the participant's clinical state.

Possibly related: An adverse event that follows a reasonable temporal sequence from administration of the study intervention of that follows a known or expected pattern of response to the study intervention, but that could readily have been produced by a number of other factors.

Unrelated: The adverse event is clearly not related to the investigational procedure (i.e. another cause of the event is most plausible; and/or a clinically plausible temporal sequence is inconsistent with the onset of the event and the study intervention and/or a causal relationship is considered biologically implausible).

8.3 Expected AEs

Expected adverse events (AEs) will be captured through interviews at 12, 24 and 36 weeks, based on the Health Status Update questionnaire. The following are expected adverse events that have been listed in the informed consent form:

- Muscle soreness
- Fatigue
- Chest pain
- Breathing problems
- Cardiac event
- Fall (with or without injury)

8.4 Reportable AEs (RAEs)

Reportable AEs are events that have potential implications for participant safety and that require individual reporting. RAEs will be defined as events that fall into at least one of the following categories:

1. Serious adverse event (SAEs) - SAEs will be defined as any adverse event that results in death, is life threatening, or places the participant at immediate risk of death from the event as it occurred, requires or prolongs hospitalization, causes

persistent or significant disability or incapacity, results in congenital abnormalities or birth defects, or is another condition which investigators judge to represent significant hazards.

2. Unexpected AEs - An unexpected AE is defined as medical events that occur during study participation, but do not commonly occur in the study population and which are not listed in the informed consent document or study protocol.
3. AEs related or possibly related to the research intervention – defined as any AE which in the opinion of the principal investigator, the incident, experience or outcome more likely than not was caused by the procedures involved in the research.

Events that cannot be clearly defined as “reportable” will be discussed with the study physician and the PI to determine if they should be reported. All reportable events will be captured on an Adverse Event form which will be filed in the participant binder and then reported using the following guidelines.

8.5 Reporting of Events

The study PI has primary responsibility for the safety of participants as it relates to the study protocol. The study coordinator will be responsible for reviewing adverse events and assuring accurate and timely reporting of the adverse events. The co-investigators (including study physician) will review, evaluate and classify adverse events and provide follow-up for events until they are resolved. The DSMB will be responsible for monitoring study data for evidence of adverse effects attributable to participation in the study. The PI will be responsible for reporting study-defined AEs and SAEs to the University of Pittsburgh institutional review board (IRB) according to their timeline and format.

<u>8.5.1 Serious Adverse Events.</u> The occurrence of SAEs will be obtained on an Adverse Event form during the 12, 24 and 36 week testing sessions and by any study staff member who learns of a serious event. All study staff will be trained to recognize when there is a SAE and to follow appropriate reporting procedures as they become aware of a reportable event.

All SAEs will be reported to the University of Pittsburgh IRB, the DSMB, and NIA within 24 hours of learning of the event. The expedited report of the SAE will be submitted by telephone, fax, or email. Recognizing that the information available during this 24 hour period may not be sufficient to permit accurate completion of the required adverse event reporting forms, a detailed written SAE report will be completed as soon as the information is available. Follow up information can be requested by the DSMB or the NIA or its representative.

<u>8.5.2 Unexpected Adverse Event.</u> An unexpected AE may be witnessed by a member of the research team or a staff member may be told about the occurrence or an unexpected event that may meet the criteria for reporting. Unexpected AEs that have a

potential relationship to study procedures and activities will be reported to the University of Pittsburgh IRB and the DSMB within 24 hours of learning of the event.

8.5.3 Adverse Events Related or Possibly Related to the Research Intervention. An AE which in the opinion of the principal investigator, the incident, experience or outcome more likely than not was caused by the procedures involved in the research will be reported to the IRB and the DSMB within 10 working days of the investigator learning of the event. If the AE is serious or unexpected it will be reported as described above.

8.6 Follow-up for Adverse Events

All RAEs will be forwarded to an Independent Safety Monitor (ISM) for adjudication and follow-up. The ISM may contact the study coordinator to request additional information from the participant, significant other, or their health care provider and may seek medical records from a physician or care setting if needed to make a determination about relatedness to the study. If the event is potentially related to the study, the ISM will contact the PI who will consider whether the event was listed in the protocol and consent form and whether modifications to the protocol and consent form should be considered. To ensure the appropriate classification of events, the clinical site physician and/or PI may also be called to review the information.
The ISM will be responsible for providing follow-up for ongoing reportable events as follows:

1. The ISM will follow up on RAEs until a final status has been determined for the event. Some RAEs may have a status that is still present at the conclusion of the study. The categories of RAE status are: not recovered/not resolved, recovered/resolved, recovered/resolved with sequelae, recovering/resolving, fatal, unknown.

2. Once the event is no longer ongoing and a final status for the event has been determined, the ISM will record the final status, enter the closed date, and sign and date the report.

8.6.1 Action Taken

The PI, in consultation with the study physician and the ISM, will decide whether or not an RAE requires that the participant be removed from the study intervention. The DSMB will be notified of the recommended course of action. Actions taken in response to the RAE will fall into one of four categories:

- No action taken
- Study procedure interrupted
- Study procedure discontinued
- Study procedure modified

8.7 Responsibilities

The PI has primary responsibility for the safety of participants as it relates to the study protocol. The study coordinator will be responsible for reviewing RAEs and assuring

accurate and timely reporting of the RAEs. The study physician and the ISM will review, evaluate, and classify RAEs and provide follow-up for events until they are resolved. The DSMB will be responsible for reviewing and monitoring data for evidence of harm attributable to participation in the study.

8.7.1 Study Physician

The study physician will be available by telephone for consultation with the study personnel during all time periods when participants are engaged in the assessments or interventions. In addition, the study physician will be responsible for reviewing RAEs requiring immediate notification of the IRB and for being “on call” for study related emergencies. We will also obtain the name and contact information for the participant’s primary care physician. In rare cases when the study physician is unavailable, the participant’s primary physician will be contacted.

8.7.2 Independent Safety Monitor (ISM)

The DSMB safety officer will serve as the ISM. The ISM will be responsible for:

- Reviewing reports of RAEs
- Confirming or refuting classification of the event as a RAE
- Requesting additional information as needed in order to make a determination
- Providing the PI with follow-up reports for ongoing RAEs as new information becomes available
- Notifying the PI of final status for the event once it has been determined

8.8 Reporting Expected AEs and RAEs to the DSMB

The DSMB will review tabulated data on non-serious and expected AEs on a semi-annual basis and monitor for adverse event rates out of proportion to those expected. The PI will forward the individual Reportable Adverse Event Records, including a narrative for each event, as well as a table showing all RAEs to the DSMB and NIA semi-annually. The DSMB will review all RAEs that are temporally related to the interventions in aggregate form at its scheduled meetings.

All RAEs that relate to hazards of the study interventions or are cause for urgent concern will be reported to the DSMB chairperson, the NIA and the University of Pittsburgh IRB immediately after recognition of their importance. If the DSMB chairperson concludes that an RAE is of universal and immediate concern, the DSMB chairperson may recommend convening the DSMB to review participant safety based on any individual report or accumulating evidence, including evidence according to treatment assignments.

9. INTERVENTION DISCONTINUATION

Certain events may result in a temporary interruption or early discontinuation of the trial assessments and interventions or components of these assessments and interventions. Please refer to the appropriate MOP chapter(s) for specific instructions on stopping criteria during screening, assessment, interventions, and follow-up assessments.

After such events occur, a participant may resume the trial intervention when the study physician and the primary care provider agree that it is appropriate. For mild problems that require temporary cessation of the intervention, the PI in consultation with the study physician and the participant, may agree to reintroduce the participant to the study intervention.

At any time, the DSMB may recommend discontinuation of any component of the intervention or intervention group of the study for any of the following reasons:

1. Compelling evidence from this or any other study of an adverse effect of the study intervention(s) that is sufficient to override the potential benefit of the interventions to the target population
2. Compelling evidence from this or any other study of a significant beneficial effect of the study intervention(s), such that it is continued denial to other study group(s) would be unethical
3. A very low probability of addressing the study goals within a feasible timeframe.

The participants' participation in this research study is completely voluntary. The participant may withdraw, at any time, their consent for participation in this research study. Any identifiable research information recorded for, or resulting from, their participation in this research study prior to the date that they formally withdrew their consent may continue to be used and disclosed by the investigators. To formally withdraw consent for participation in this research study the participant should provide a written and dated notice of this decision to the principal investigator of this research study. If the participant withdraws from the intervention, study staff will ask permission to continue to follow the participant for follow-up assessment. If participation is discontinued for medical reasons and the participant is unable to complete the performance-based testing, all attempts will be made to obtain the self-reported outcomes.

10 DATA ANALYSES

All statistical analyses will be performed using SAS® version 9.3 (SAS Institute, Inc., Cary, North Carolina) based on the **intention-to-treat** philosophy. First, we will evaluate the distributional characteristics of the data set, the prevalence of missing values and general data quality using appropriate descriptive statistics (e.g. mean, median, standard deviation, range, frequencies, percentages) and graphical summarizations (histograms, boxplots, probability plots, scatterplots, lineplots) for all variables for each walk speed stratum for each intervention group for each available time point, as well as change scores from baseline for outcome variables. Second, the baseline pre-intervention values of these variables will be compared between the two intervention arms using independent samples *t*- or Wilcoxon rank sum tests, as appropriate, for continuous variables and a chi-square or Fisher's exact tests, as appropriate, for categorical variables. Although no significant differences are expected due to randomized treatment assignment and the relatively large sample size, any variables found to be significantly different will be noted and accounted for, by controlling for them as additional covariates in supplements to main analyses. Third,

main analyses to address the specific aims and hypotheses will be performed as outlined below. If an analysis of residuals reveals violations of standard assumptions of linear models, we will use a Box-Cox transformation(78) to the response variable before fitting the models. Multiple imputation will be used to account for any missing data in the main analysis.(79, 80) Finally, exploratory follow-up analyses will be performed as outlined below.

10.1 Main Analysis.

First, we will fit a series of linear mixed models using the SAS® MIXED procedure with change from baseline in each of the continuous outcome measures as the dependent variable; intervention arm (standard/standard plus), follow-up time point (12-/24-/36-week) and their interaction as fixed effects of interest; baseline value of outcome as a fixed-effect covariate; and a subject random effect to account for multiple follow-up assessments from the same patients over time and resulting stochastic non-independence of observations. We will appropriately construct means contrasts to compare the two intervention gains at 12 weeks (immediate effect), 24 weeks (delayed effect) and 36 weeks (sustained effect). Test of significance at $\alpha=0.05$ for gait speed outcome at 12 weeks will be considered the formal test of the primary hypothesis 1.1. Second, we will repeat the above mixed models analysis entirely, but with stratification by baseline gait speed (<1.0/1.0+ m/s). Tests of significance for gait speed outcome at 12 weeks in each of the two strata will be considered formal tests of the primary hypothesis 1.2. Statistical significance of at 24- and 36-week comparisons or other outcomes will be considered as formal tests of the secondary hypotheses involving activity and participation as well as delayed and sustained effects. Third, we will repeat the analyses after controlling for covariates identified a priori or found to be different between intervention arms by including them as additional fixed effects in supplements to main analyses.

10.2 Exploratory Analyses.

To address our subgroup analysis exploratory aim, we will repeat the main analyses after stratification by various other baseline participant characteristics (besides baseline gait speed) to identify, if any, subgroups which are most/least likely to benefit from interventions. A priori, we anticipate that stratification by baseline walking confidence and cognitive function may show differential gains due to the two interventions. We are aware that any definitive subgroup analyses should be supported by strong evidence in the form of a significant interaction effect.(81) Tests of interaction generally have low statistical power, and it is not feasible in the proposed Phase II efficacy trial to recruit the large number of participants required for definitive subgroup analyses beyond the already included pre-planned subgroups based on baseline walking speed. Thus, we will interpret our subgroup analyses with caution and an exploratory rather than a confirmatory philosophy, whose results are to be confirmed in a larger subsequent trial.

10.3 Compliance and Dropout Analyses.

We will estimate compliance by the number of sessions attended and estimate the proportion of subjects in each group with various levels of compliance. We also will

calculate the proportion of subjects missing each session to describe the pattern of compliance. Dropout rates will be calculated as proportions of subjects randomized, and as a cumulative probability of remaining in the study, using survival analysis techniques such as the Kaplan-Meier product-limit estimator.(82) Unlike proportions, the latter statistics, which can be estimated at various times following randomization, take into account when dropouts occur. As with the compliance measure, these statistics will be calculated separately for each treatment group. The information contained in these descriptive analyses may help improve our retention strategies.

10.4 Interim Analysis

We do not plan to perform any interim analyses for efficacy. We will follow the recommendations of the external Data and Safety Monitoring Board (DSMB) to establish the specific rules for interim analyses for safety, either before commencement or very early stages of the trial. Specifically, we a priori plan to use the proportion of completed person-time of follow-up as the information statistic; test-statistic for comparison of proportions of (serious) adverse events to define the Brownian motion process; and an alpha-spending function discussed in Lan and DeMets (1983) to define a safety boundary. We will defer to the recommendation of DSMB regarding specific time points for interim safety analyses (expecting them to be approximately annually or otherwise coincide with DSMB meetings); whether the functional form of the alpha-spending function should be Pocock, Obrien-Fleming or another function; and any other aspect of interim safety monitoring.

10.5 Sample Size Adequacy

Sample size is estimated based on preliminary data from our pilot studies; our ability to detect statistical significance in two-tailed tests conducted at the $\alpha=0.05$ level unless otherwise noted; and conservative expected dropout rates of 10, 15 and 20% during the 12-, 24- and 36-week follow-up. Thus, we conservatively anticipate 55, 52 and 49 participants to complete the 12-, 24- and 36-week assessments, respectively. We used standard sample size and power computation methods available in the literature and commercial software (PASS®; Number Cruncher Statistical Systems, Inc., Kaysville, Utah).

10.5.1 Primary Outcome

Our primary outcome on which the sample size justification is based is gait speed. Our preliminary data suggest a conservative estimate of between-subject standard deviation in baseline gait speed is 0.17 m/s and baseline to follow-up change in gait speed is 0.15 m/s. Moreover, a meaningful change in gait speed is approximately 0.10 m/s.(11) With the proposed number of 124 in each intervention, we will have >99% power to detect statistical significance of a between-intervention difference of such magnitude in 12-week change (primary hypothesis 1.1). We anticipate that approximately half of the participants will belong to the slower baseline gait speed stratum. Thus, with the expected number of 62 participants within each arm within each stratum, we will be able to detect statistical significance of a between-intervention difference in 12-week change

within each stratum (slower/faster baseline gait speed) with 96% statistical power (hypothesis 1.2). For 24- and 36-week gait speed change, we will have 92% and 90% statistical power, respectively, within each stratum (delayed effect and sustained effect hypotheses). If the anticipated split between slower and faster walker strata turns out to be no worse than 55%-45%, we will still have ≥90% power for both strata for hypothesis 1.2; and if it is no worse than 67%-33%, we will still have ≥80%.

10.5.2 Secondary and Tertiary Outcomes

Regarding secondary and tertiary outcomes, our preliminary data suggest conservative estimates of between-subject standard deviations in baseline measures of 0.9 s (Figure 8 time), 8.8 points (LLFDI function), 12.1 points (LLFDI disability limitation) and 53.1 m (6-minute walk test). Baseline to follow-up change standard deviations, respectively, were 1.08 s, 5.5 points, 11.8 points and 42.8 m. With the proposed sample size of 124 (and 98 anticipated completers at 36 weeks) per intervention arm, we will be able to detect statistical significance of between-intervention differences as small as 0.43 s, 2.2 points, 4.7 points and 17.2 m, respectively, with 80% power in two-tailed tests. These differences for the sustained effect hypotheses correspond to small-moderate Cohen's effect sizes of 0.25-0.48 for all secondary outcomes.(83) Statistical power for the immediate and delayed effect hypotheses should be even greater due to greater numbers of participants in study at the earlier 12- and 24-week time points.

In summary, we have proposed an adequate sample size for successfully addressing the aims of the project, with a higher degree of statistical sensitivity reflected by ≥90% power for detecting meaningful differences in the primary outcome gait speed; and adequate statistical sensitivity reflected by ≥80% power for detecting small to moderate effects in secondary and tertiary outcomes.

10.6 Treatment Assignment Procedures - Randomization

We will use the high quality pseudo-random deviate generator available in SAS® (SAS Institute, Inc., Cary, North Carolina) to randomize participants to standard or standard-plus interventions in a 1:1 ratio, stratified by whether a slower (<1.0 m/s) or faster (≥1.0 m/s) walker at baseline. Within each baseline gait speed stratum, we will do a blocked randomization to force continued approximate balance between the numbers of subjects in each arm during recruitment. The block size will be randomly selected to be 4 or 6 to prevent personnel from predicting treatment arm. For example, if the first two participants are randomized to active interventions, the assignment of the next participant cannot with certainty be predicted to be control. The study statistician will create randomization schedules for the two strata which contains a randomization sequence number (different from a participant's study identification number) and assigned arm. Randomization sequence numbers will be readily distinguishable between the strata (ie. starting from 4001 for slower and 8001 for faster baseline walkers). Then he will pass the randomization schedules to data management personnel so that they can be incorporated into the data management system itself. At the time of randomization, research personnel will indicate the eligible participant to be randomized in the data management system. The system will first determine the stratum of the participant being randomized, and then use the next available randomization sequence number for that particular stratum to determine the intervention

assignment based on the schedule constructed by the study statistician, record the intervention assignment in a restricted location in the database inaccessible by the blinded personnel, and retire the used randomization sequence number and randomized participant number from the randomization process. Personnel assessing follow-up outcomes will be blind to intervention assignment.

11 DATA COLLECTION AND QUALITY ASSURANCE

11.1 Data Collection Forms

Data collection will consist of a combination of paper forms and direct data entry. Data collected on paper forms will be entered into the electronic database by the research staff. A complete set of paper forms will be available at all times to be used if the database is temporarily unavailable. Please see the Manual of Procedures for a copy of all data entry forms.

Screening and baseline data collection, which will occur prior to randomization, will be conducted by research staff trained in the outcomes and may include the study coordinator if necessary. Outcome assessments at 12, 24 and 36 weeks will only be conducted by research staff trained in the outcomes and who are blinded to the intervention group assignment.

Participants' confidentiality will be protected in the data collection process. All study personnel are certified in Research Practice Fundamentals, Human Subjects Research Module. Consent forms and paper data collection forms will be stored in locked file cabinets. All computers are password protected. The database will be web-based and accessible with a username and password. Only authorized team members will have access to personal information needed for tracking and informed consent.

11.2 Data Management

The PI will oversee all aspects of data management. The Data Center (DC) will create an electronic System for Data Management (eSYSDM), based on detailed study protocols and requirements that includes an electronic case report form and a tracking system. The eSYSDM is developed using .NET 2.0 to create the interface and SQL for the database. The DC will work closely with investigative team and other study personnel to ensure that protocols are being followed, data integrity and confidentiality are maintained, and that the data contains a minimum amount of missing data. All study files residing in designated network folders will be backed-up daily and archived weekly. The weekly archived files are maintained for 1 year until the data are erased. All study subjects will be assigned unique study identifiers that will appear on all data collection instruments, tapes, documents, and files used in the statistical analysis and manuscript preparation. Only authorized team members will have access to personal information needed for tracking and informed consent. Other data quality assurance measures will include detailed documentation of computer operations and data editing procedures and regular meetings with project staff to review any changes in procedure. The DC also

has specific data quality measures that will be implemented. These include data verification, built in data validation mechanisms such as logic and out of range data checks, and repeated evaluation of the data collection and entry process.

11.3 Quality Assurance

Ongoing QA monitoring will be performed by the data center, study statistician (Dr. Perera) and the study coordinator. The DC or Dr. Perera will perform exploratory statistical investigations of aggregate data to identify unusual patterns and distributions. Monthly reports will be generated showing the frequency of missing data, delinquent forms, and other study performance parameters. These reports will be reviewed by the study coordinator who will promptly initiate action to remedy any problems, and will perform follow-up evaluations of actions taken, if necessary.

A quality assurance meeting will be held with all study staff prior to the enrollment of the first subject. Additional QA meetings will be held annually during the enrollment and follow-up period. If problems are identified, the PI will develop a corrective action plan for the staff with clearly defined tasks and timelines. The PI will track the implementation of the plan and assure that all tasks are completed within the defined timeframe.

At the QA meetings, study staff will discuss any difficulties they are having and will work collectively with the study investigators to resolve the problems. Additional meetings will be held if there are concerns about data quality or protocol adherence.

The purposes of the QA meetings will be to:

- Assure the rights and safety of participants
- Assure that informed consent has been obtained and documented in accordance with the protocol and NIH regulations
- Verify adherence to the protocol and exam staff knowledge via discussions
- Discuss screenings, informed consent process, assessments and interventions and to confirm that staff are following good clinical practice
- Ensure secure maintenance of required documents
- Review participant files for completeness
- Assure that information recorded on forms is complete and accurate
- Assure accurate reporting and documentation of all AEs
- Review data quality reports

Protocol deviations will be captured on the protocol deviation form and entered into the database. Protocol deviations will reviewed and discussed at monthly team staff meetings. The study coordinator, with input from the study investigators, will promptly initiate action to remedy any problems, and will perform follow-up evaluations of actions taken, if necessary.

12 PARTICIPANT RIGHTS AND CONFIDENTIALITY

12.1 Institutional Review Board (IRB) Review

The study protocol, the informed consent document and any subsequent modifications will be reviewed and approved by the University of Pittsburgh IRB.

12.2 Informed Consent Forms

All potential participants will be adults (65 years of age or older) who are capable of providing direct consent for their participation in the study. Written informed consent will be obtained at the clinic screening visit prior to performing any of the clinic screening procedures. One of the study investigators will explain the study and the participant will be given a copy of the consent form to read. The consent form will describe the purpose of the study, the procedures to be followed, and the risks and benefits of participation. The investigator will answer any questions that the participant may have about the study. Finally, the participant will be asked to sign and date the consent form. The participant will be given a copy of the consent form for their records.

12.3 Participant Confidentiality

Participant privacy will be protected throughout the research process. Research assessments and interventions are conducted in the PT-CTRC located in the Bridgeside Point Building at the University of Pittsburgh. This space includes several small rooms that can be used to privately conduct questionnaires and simple physical examination measures. Many of the walking assessments are done in the open area of the center, which includes the oval track, the computerized walkway system and treadmills. Participants may be screened individually, which further protects their privacy. Participants are informed via the consent process that the treatment programs may be conducted in small groups. Although not completely private, this level of exposure to others during exercise is similar to what one might experience at a physical therapy appointment or during an exercise routine at a public gym. This research will not involve the use or disclosure of existing identifiable medical information, such as personal medical or hospital records.

Participants' confidentiality will be protected in the data collection process. Consent forms and data collection forms that identify the participant by name will be stored in a locked cabinet. All computers are password protected. If the data are used in scholarly presentations or journal articles, the investigators will protect the anonymity of individual participants and will report only aggregate data (eg group means) where appropriate. The Principal Investigator will review data confidentiality processes monthly or as indicated with the project staff (Study coordinator and Physical Therapists). The Investigators are all certified in Research Practice Fundamentals, Human Subjects Research Module.

12.4 Study Discontinuation

The study may be discontinued at any time by the IRB, the NIA, the OHRP, or other

government agencies as part of their duties to ensure that research participants are protected.

13 PUBLICATION OF RESEARCH FINDINGS

Publications will be operationally defined as manuscripts for publications; abstracts for platform or poster presentation at scientific meetings and other professional meetings; slides for presentation at scientific and other meetings; doctoral dissertations; and master's theses.

The goal of the publication policy is to encourage and facilitate publication of study results. The purposes of this policy are to ensure the following:

- PRIMA publication will be of the highest scientific quality
- PRIMA will be described in a consistent manner across publications
- Measures are reported in consistent ways across publications
- Proper acknowledgements are included
- Appropriate authorship credit is determined prior to submission of manuscripts for publication consideration.

Publications from PRIMA will be overseen by the PI and Co-Investigators.

14 ANCILLARY STUDY POLICIES

An ancillary study will be defined as a study that (1) uses supplementary data that will be collected on participants who are recruited in PRIMA, over and above the data collection required by the PRIMA protocol, (2) collects biological specimens (e.g. blood) or performs diagnostic tests (e.g. bone density scans); and/or (3) collects data on subjects not enrolled in PRIMA but who may be compared to PRIMA subjects (e.g. participant who receive an alternative intervention). Ancillary studies will be distinct from databank studies, which use data previously collected on participants who are enrolled in PRIMA.

Ancillary studies will be reviewed and approved by the PI and CO-I's prior to initiation to ensure they do not conflict with the main study protocol. All approved ancillary studies will also be reviewed by the DSMB and NIA prior to initiation. If approved, the ancillary study PI will report to the DSMB on the same schedule as the main study. Review by the PI and Co-I's will also be required for presentation or publication of ancillary study results.

PRIMA investigators will be encouraged to consider ancillary studies and to involve other investigators, within and outside of PRIMA personnel. Participation in an ancillary study will be subject to approval by the PI, Co-I's and DSMB. The following factors will be considered in determining approval of the proposed ancillary study:

1. Participant burden

    a. The proposed study must be acceptable to the participants (e.g. in terms of time, discomfort, privacy, etc).
    b. The proposed study must not reduce enrollment or hamper continued participation in the main study.
2. Study interference
    a. The proposed study must not interfere with the other parts of the main study.
    b. The proposed study must put little to no additional demands on the PRIMA resources.
3. The prosed study must be of the highest scientific merit.
4. The investigators must have adequate resources to effectively complete the ancillary study including:
    a. Sufficient budget
    b. Staff having the required expertise to meet the objectives of the project.

15 REFERENCES

1. Guralnik J, Ferrucci L, Pieper C, Leveille S, Markides K, Ostir G, et al. Lower extremity function and subsequent disability: consistency across studies, predictive models, and value of gait speed alone compared with the short physical performance battery. J Gerontol Med Sci. 2000;55A:M221-M31.
2. Cesari M, Kritchevsky S, Bauer D, Visser M, Rubin S, Harris T, et al. Prognostic value of usual gait speed in well-functioning older people--results from the Health, Aging and Body Composition Study. J Am Geriatr Soc. 2005;53:1675-80.
3. Studenski S, Perera S, Patel K, Rosano C, Faulkner K, Inzitari M, et al. Gait speed and survival in older adults. JAMA. 2011;305(1):50-8.
4. Guralnik J, Ferrucci L, Simonsick E, Salive M, Wallace R. Lower extremity function in persons over the age of 70 years as a predictor of subsequent disability. New Engl J Med. 1995;332:556-61.
5. Guralnik J, Simonsick E, Ferrucci L, Glynn R, Berkman L, Blazer D, et al. A short physical performance battery assessing lower extremity function: Association with self-reported disability and prediction of mortality and nursing home admission. J Gerontol. 1994;49:M85-M94.
6. Fried L, Bandeen-Roche K, Chaves P, Johnson B. Preclinical Mobility Disability Predicts Incident Mobility Disability in Older Women. Journal of Gerontology. 2000;55A(1):M43-M52.
7. Hoffman J, Ciol M, Huynh M, Chan L. Estimating transitions probabilities in mobility and total costs for Medicare beneficiaries. Arch Phys Med Rehabil. 2010;91:1849-55.
8. Pate R, Pratt M, Blair S, Haskell W, Macera C, Bouchard C, et al. Physical activity and public health: a recommendation from the Centers of Disease Control and Prevention and the American College of Sports Medicine. JAMA. 1995;273(5):402-7.
9. Nelson M, Rejeski W, Blair S, Duncan P, Judge J, King A, et al. Physical activity and public health in older adults: recommendation from the American College of Sports Medicine and the American Heart Association. Med Sci Sports. 2007;39(8):1435-45.
10. LIFE S, Investigators. Effects of a physical activity intervention on measures of physical performance: results of the Lifestyle Interventions and independence for elders pilot (LIFE-P) study. J Gerontol Med Sci. 2006;61A:1157-65.

11. Perera S, Mody S, Woodman R, Studenski S. Meaningful change and responsiveness in common physical performance measures in older adults. J Am Geriatr Soc. 2006;54:743-9.
12. Brach J, Studenski S, Perera S, VanSwearingen J, Newman A. Stance time and step width variability have unique contributing impairments in older persons. Gait Posture. 2008;27:431-9.
13. Ferrucci L, Baninelli S, Benvenuti E, DiIorio A, Macchi C, Harris T, et al. Subsystems contributing to the decline in ability to walk: Bridging the gap between epidemiology and geriatric practice in the InCHIANTI study. J Am Geriatr Soc. 2000;48(12):1618-25.
14. Haley S, Jette A, Coster W, Kooyoomijian J, Levenson S, Heeren T, et al. Late life function and disability instrument:II. Development and evaluation of the function component. J Gerontol. 2002;57A:M217-M22.
15. Milton J, Small S, Solodkin A. On the road to automatic: dynamic aspects in the development of expertise. J Clin Neurophys. 2004;21:134-43.
16. Brooks V. The Neural Basis of Motor Control. New York: Oxford University Press; 1986.
17. McArdle W, Katch F, Katch V. Exercise Physiology: Energy, Nutrition, and Human Perofrmance. Fifth ed. Baltimore, MD: Lippincott Williams & Williams; 2001.
18. VanSwearingen J, Perera S, Brach J, Wert D, Studenski S. Exercise to improve gait efficiency: impact on activity and participation in older adults with mobility limitations. Phys Ther. 2011;91:1740-51.
19. de V, PL, Samson M, van M, NL, Duursma S, Verhaar H. Functional-task exercise versus resistance strength exercise to improve daily function in older women: a randomized controlled trial. J Am Geriatr Soc. 2005;53:2-10.
20. Judge J. Balance training to maintain mobility and prevent disability. Am J Prev Med. 2003;25(3 Suppl 2):150-6.
21. Brown M, Holloszy J. Effects of a low intensity exercise program on selected physical performance characteristics of 60- to 71-year olds. Aging (Milano). 1991;3:129-39.
22. Fiatarone M, Marks M, Ryan E, Meredith N, Lipsitz C, Evans W. High intensity strength training in nonagenarians. Effects on skeletal muscle. JAMA. 1990;263:3029-34.
23. Judge J, Underwood M, Gennosa T. Exercise to improve gait velocity in older persons. Archives of Physical Medicine and Rehabilitation. 1993;74(4):400-6.
24. Fiatarone M, O'Neill E, Ryan N, Clements K, Solares G, Nelson M, et al. Exercise training and nutritional supplementation for physical frailty in very elderly people. New Engl J Med. 1994;330:1769-75.
25. Topp R, Mikesky A, Wigglesworth J, Holt W, Edwards J. The effect of a 12-week dynamic resistance strength training program on gait velocity and balance of older adults. Gerontologist. 1993;33(4):501-6.
26. Menz H, Lord S, Fitzpatrick R. Age-related differences in walking stability. Age Aging. 2003;32:137-42.
27. Morgan M, Phillips J, Bradshaw J, Mattingley J, Iansek R, Bradshaw J. Age-related motor slowness: simply strategic? Journal of Gerontology. 1994;49(3):M133-M9.
28. Welford A. Motor Skills and Aging. In: Mortimer J, Pirozzolo F, Maletta G, eds. The Aging motor System. New York: Praeger Publishers; 1982:152-87.
29. Welford A. Between bodily performance and slowing with age. Exp Aging Res. 1984(10):73-88.
30. VanSwearingen J, Perera S, Brach J, Cham R, Rosano C, Studenski S. A randomized trial of two forms of therapeutic activity to improve walking: effect on the energy cost of walking. J Gerontol A Biol Sci Med Sc. 2009;64A:1190-8.
31. Brach J, VanSwearingen J, Perera S, Wert D, Studenski S. Motor learning versus standard walking exercise in older adults with subclinical gait dysfunction: A randomized clinical trial. J Am Geriatr Soc. 2013;61:1879-86.

32. VanSwearingen J, Perera S, Brach J, Cham R, Rosano C, Studenski S**.** Exercise to reduce the energy cost of walking: a randomized trial. J Gerontol Med Sci. 2009;64(1):1190-8.
33. Brach J, Lowry K, Perera S, Wert D, Hornyak V, Studenski S, et al. Improving motor control in walking: a randomized clinical trial in older adults with subclinical walking difficulty. . Arch Phys Med Rehabil. 2014;in press.
34. Kramer M, McWilliams J, Chen H, Siminerio L**.** A community-based diabetes prevention program: evaluation of the Group Lifestyle Balance Program devilvered by diabetes educators. The Diabetes Educator. 2011;37(5):659-68.
35. ACSM's Guidelines for Exercise Testing and Prescription. 5th ed. Baltimore, MD: Williams & Wilkins; 1995.
36. Nelson W**.** Physical principles for economies of skilled movements. Biol Cybernetics. 1983;46:135-47.
37. Daly J, Ruff R**.** Construction of efficacious gait and upper limb functional interventions based on brain plasticity evidence and model-based measures for stroke patients. The Scientific World Journal. 2007;7:2031-45.
38. Lay B, Sparrow W, Hughes K, O'Dwyer N**.** Practice effects on coordination and control, metabolic energy expenditure, and muscle activation. Human Movement Science. 2002;21:807-30.
39. Newman M, Dawes H, van d, Berg, M, Wade D, Burridge J, Izadi H**.** Can aerobic treadmill training reduce the effort of walking and fatigue in people with multiple sclerosis: a pilot study. Multiple Sclerosis. 2007;13:113-9.
40. Gentile A**.** Skill acquisition: action, movement, and neuromotor processes. In: JH C, RB S, J G, AM G, JM H, eds. Movement Sciences. 1 ed. Rockville: Aspen Publishers; 1987:93-154.
41. Polcyn A, Lipsitz L, Kerrigan C, Collins J**.** Age-related changes in the initiation of gait: degradation of central mechanisms for momentum generation. Arch Phys Med Rehabil. 1998;79:1582-9.
42. Capaday C**.** The special nature of human walking and its neural control. Trends in Neurosciences. 2002;25(7):370-6.
43. Alexander R**.** Walking made simple. Science. 2005;308:58-9.
44. Schmidt R**.** Organizing and Scheduling Prcatice. In: RA S, ed. Motor Learning and Practice: From Principles to Practice. Champaign, IL: Human Kinetics Books; 1991:199-225.
45. Brach J, VanSwearingen J**.** Interventions to improve walking in older adults. Curr Transl Geriatr and Exp Gerontol Rep. 2013;2:230-8.
46. Brach J, Perera S, Studenski S, Newman A**.** Reliability and validity of measures of gait variability in community-dwelling older adults. Arch Phys Med Rehabil. 2008;89:2293-6.
47. Mangione K, Craik R, McCormick A, Blevins H, White M, Sullivan-Marx E, et al. Detectable changes in physical performance measures in elderly African Americans. Phys Ther. 2010;90(6):921-7.
48. Harada N, Chiu V, Damron-Rodriguez J, Fowler E, Siu A, Reuben D**.** Screening for balance and mobility impairment in elderly individuals living in residential care facilities. Physical Therapy. 1995;75(6):462-9.
49. Hardy S, Perera S, Roumani Y, Chandler J, Studenski S**.** Improvement in usual gait speed predicts better survival in older adults. J Am Geriatr Soc. 2007;55(11):1727-34.
50. Brach J, et a**.** Meaningful change in measures of gait variability in older adults. J Am Geriatr Soc. 2008;submitted.
51. Callahan D, Phillips E, Carabello R, Frontera W, Fielding RA**.** Assessment of lower extremity muscle power in functionally-limited elders. Aging Clin Exp Res. 2007;19(3):194-9.

52. Butland R, Pang J, Gross E, Woodcock A, Geddes D. Two-, six-, and 12-minute walking tests in reespiratory disease. BMJ. 1982;284:1607-8.
53. Harada N, Chiu V, Stewart A. Mobility-related function in older adults: assessment with a 6-minute walk test. Arch Phys Med Rehabil. 1999;80:837-41.
54. Guyatt G, Sullivan M, Thompson P. The 6-minute walk: a new measure of exercise capacity in patients with chronic heart failure. Can Med Assoc. 1985;132:919-23.
55. Solway S, Brooks D, Lacasse Y, Tomas S. A qualitative, systematic overview of the measurement properties of the functional walk tests used in the cardioresepiratory domain. Chest. 2001;119:256-70.
56. Jones C, Rikli R, Max J, Noffal G. The reliability and validity of a chair Sit-and-Reach test as a measure of hamstring flexibility in older adults. Research Quarterly for Exercise and Sport. 1998;69(4):338-43.
57. Menz H, Lord S, Fitzpatrick R. Acceleration patterns of the head and pelvis when walking on level and irregular surfaces. Gait Posture. 2003;18:35-46.
58. Brach J, McGurl D, Wert d, VanSwearingen J, Perera S, Cham R, et al. Validation of a measure of smoothness of walking. J Gerontol A Biol Sci Med Sc. 2011;66:136-41.
59. Gabell A, Nayak U. The effect of age and variability in gait. Journal of Gerontology. 1984;39(6):662-6.
60. Brach J, Studenski S, Perera S, VanSwearingen J, Newman A. Gait variability and the risk of incident mobility disability. J Gerontol Med Sci. 2007;62A:983-8.
61. Perera S, Brach J, Talkowski J, Wert d, Studenski S. Measuring stride time variability: estimating test-retest reliability and required walk length using bootstrapping. Program & Abstracts of the ISPGR 18th International Conference. 2007:55-6.
62. Brach J, Berlin J, VanSwearingen J, Newman A, Studenski S. Too much or too little step width variability is associated with a fall history in older persons who walk at or near normal gait speed. J Neuroengineering Rehabil. 2005;2(21).
63. Boyd R, Rodda J, Olesch C, et a. High- or low-technology measurements of energy expenditure in clinical gait analysis? Dev Med Child Neurol. 1999;41:676-82.
64. MacGregor J. The objective measurement of physical performance with long term ambulatory physiological surveillance equipment (LAPSE). In: FD S, EB R, L G, eds. Proceedings of the Third International Symposium on Ambulatory Monitoring. London: Academic Press; 1980:29-39.
65. Waters R, Lunsford B. Energy cost of paraplegic ambulation. J Bone Joint Surg. 1985;67:1245-50.
66. Bernardi M, Macaluso A, Sproviero E, Castellano V, Coratella D, Felici F, et al. Cost of Walking and Locomotor Impairment. Journal of Electromyography and Kinesiology. 1999;9(2):149-57.
67. Macko R, Katzel L, Yataco A, Tretter L. Low-velocity graded treadmill stress testing in hemiparetic stroke patients. Stroke. 1997;28(5):988-92.
68. World H, Organization. ICF: International Classification of Functioning, Disability and Health., 5/22/1 AD 1905. Geneva, Switzerland: World Health Organization; 2001.
69. Jette A, Haley S, Coster W, Kooyoomijian J, Levenson S, Heeren T, et al. Late life function and disability instrument: I. Development and evaluation of the disability component. J Gerontol. 2002;57A:M209-M16.
70. Welk G, Schaben J, Morrow J. Reliability of accelerometry-based activity monitors: a generalizability study. Med Sci Sports Exer. 2004;36(9):1637-45.
71. Bassett D, Ainsworth B, Swartz A, Strath S, O'Brien W, King G. Validity of four motion sensors in measuring moderate intensity physical activity. Med Sci Sports Exer. 2000;32:S471-S80.
72. Reitan R, Wolfson D. The Hasltead-Reitan Neuropsychological Test battery: Therapy and Clinical Interpretation. Tucson, AZ: Neuropsychological Press; 1985.

73. Yesavage J, Brink T, Rose T, Lum O, Huang V, Adey M. Development and validation of a geriatric depression screening scale: a preliminary report. J Psychiatr Res. 1982;17(1):37-49.
74. Buysse D, Reynolds C, 3rd, Monk T, Berman S, Kupfer D. The Pittsburgh Sleep Quality Index: a new instrument for psychiatric practice and research. Psychiatry Res. 1989;28:193-213.
75. Peel C, Sawyer B, Roth D, Brown C, Bodner E, Allman R. Assessing Mobility in Older ADults: The UAB Study of Aging Life-Space Assessment. Physical Therapy. 2005;85(10):1008-19.
76. Newell A, VanSwearingen J, Hile E, Brach J. The modified gait efficacy scale: establishing the psychometric properties in older adults. Phys Ther. 2012;92:318-28.
77. Gardner M, Robertson C, Campbell A. Exercise in preventing falls and fall related injuries in older people: a review of randomized controlled trials. Br J Sports Med. 2000;34:7-17.
78. Box G, Cox D. An analysis of transformations. Journal of the Royal Statistical Society-Series B. 1964;26:211-43.
79. Rubin D. Multiple Imputation for Nonresponse in Surveys: John Wiley and Sons; 1987.
80. Rubin D. Multiple imputation after 18+ years. Statistics in Medicine. 1991;14:1913-25.
81. Wang R, Lagakos S, Ware J, Hunter D, Drazen J. Statistics in medicine-reporting of subgroup analyses in clinical trials. N Engl J Med. 2007;357(21):2189-94.
82. Lawless J. Statistical Models and Methods for Lifetime Data. New York: Wiley; 2002.
83. Cohen J. Satistical Power Analysis for the Behavioral Sciences. New York: Academic Press; 1977.

TASK SPECIFIC TIMING AND COORDINATION EXERCISES TO IMPROVE MOBILITY IN OLDER ADULTS

(PRIMA: Program to Improve Mobility in Aging)

Principal Investigator:

Jennifer S. Brach, PhD, PT

Supported by:

The National Institute on Aging

AG045252

TASK SPECIFIC TIMING AND COORDINATION EXERCISES TO IMPROVE MOBILITY IN OLDER ADULTS

PROTOCOL (Version 1.4)

Summary of Modifications to Protocol Version 1.2 for Version 1.3

Version (date)	Section	Brief Summary of Modification
Version 1.1 (2/9/2016)	4.1	Removed inclusion criteria #5 – Not meeting physical activity recommendations defined as reporting less than 150 minutes of moderate intensity activity per week in the past month. This question will remain on phone screen so we can track information.
Version 1.2 (3/15/2016)	5.4	Updated strength training description—all subjects will completed hip extension and abduction strengthening as well as 1-2 other LE strengthening exercises at the PTs discretion
Version 1.2 (3/15/2016)	6.1	Changed timepoint for "medical clearance to participate" to clinic screen; removed CHAMPS questionnaire; added Life Space Assessment and Pitt Sleep Quality Index for baseline, 12 week, 24 week, and 36 week timepoints
Version 1.2 (3/15/2016)	6.2.3	Removed CHAMPS; added Life Space Assessment
Version 1.3 (6/6/2016)	5.6.2	Changed monthly phone calls to monthly check ins to be documented by study coordinator
Version 1.3 (6/6/2016)	5.7	Updated requirements for schedule intervention visits to include first session 8-14 days after baseline testing; added randomization timing to details

Summary of Modifications to Protocol Version 1.3 for Version 1.4

Version (date)	Section	Brief Summary of Modification
Version 1.4 (8/19/2016)	8.5-8.7	Updated information on who RAEs should be reported to and in which order. SAEs that are not related to the protocol will not be considered RAEs.

TABLE OF CONTENTS

Page

Task Specific timing and coordination exercises to improve mobility in older adults i

TABLE OF CONTENTS iii

PRÉCIS vii

STUDY TEAM ROSTER 1

Principal Investigator: 1

Co-Investigators: 1

1 Study objectives 2

1.1 Primary Objective 2

1.2 Secondary Objectives 2

2 BACKGROUND AND RATIONALE 2

2.1 Exercise for Health Promotion: Have we forgotten the nervous system? 3

2.2 Why would the standard-plus intervention impact activity and participation? 3

2.3 Why would the benefits of the standard-plus intervention be sustained after the intervention has ended? 4

2.4 Study Rationale 4

3 STUDY DESIGN 7

3.1 Overview and Study Design 7

4 SELECTION AND ENROLLMENT OF PARTICIPANTS 7

4.1 Inclusion Criteria 8

4.2 Exclusion Criteria 8

4.3 Study Enrollment Procedures 9

4.3.1 Identifying and Recruiting Participants 9

5 STUDY INTERVENTIONS 9

5.1 Overview 9

5.2 Blinding and Study Staff 10

5.3 Group Lifestyle Balance^{TM} (GLB) - Behavioral Intervention 10

5.4 Standard Intervention (include progression and modifications) 10
5.5 Standard-Plus Intervention (include progression and modifications)................ 11
5.6 Treatment Fidelity Plan.. 12
5.6.1 Initial Training for Procedural Reliability... 12
5.6.2 Ensuring Ongoing Competency.. 12
5.7 Scheduling Intervention Visits.. 13
5.8 Adherence Assessment 13

6 STUDY PROCEDURES.. 14

6.1 Schedule of Evaluations... 15
6.2 Description of Evaluations... 18
6.2.1 Screening (Phone and Clinic) 18
6.2.2 Baseline... 19
6.2.3 Follow-up Visits 23

***7* SAFETY ASSESSMENTS 24**

7.1 Participant Safety Parameters: Methods and Timing.. 24
7.1.1 Screening.. 24
7.1.2 Safety Considerations for Assessments and Interventions................ 24
8. Adverse Events and Serious Adverse Events 26
8.6 Follow-up for Adverse Events.. 30

9. INTERVENTION DISCONTINUATION 31

10 Data Analyses.. 32

10.1 Main Analysis. 33
10.2 Exploratory Analyses.. 33
10.3 Compliance and Dropout Analyses.. 34
10.4 Interim Analysis.. 34
10.5 Sample Size Adequacy 34
10.5.1 Primary Outcome 34
10.5.2 Secondary and Tertiary Outcomes... 35
10.6 Treatment Assignment Procedures - Randomization.. 35

11 DATA COLLECTION AND QUALITY ASSURANCE 36

11.1 Data Collection Forms.. 36
11.2 Data Management 36

11.3 Quality Assurance....37

12 PARTICIPANT RIGHTS AND CONFIDENTIALITY 38

12.1 Institutional Review Board (IRB) Review....38
12.2 Informed Consent Forms....38
12.3 Participant Confidentiality....38
12.4 Study Discontinuation....39

13 PUBLICATION OF RESEARCH FINDINGS 39

14 Ancillary Study Policies 39

15 REFERENCES....40

PRÉCIS

Study Title

Task Specific Timing and Coordination Exercise to Improve Mobility in Older Adults

Objectives. The primary objective of the proposed project is to evaluate the impact of adding timing and coordination training to standard strength and endurance training on mobility. Secondary objectives include examining 1) additional outcomes representing the components of the intervention and measures of activity and participation, 2) the delayed and sustained effects of the intervention, and 3) the effects of the intervention within various other subgroups of interest.

Design and Outcomes. This is a 5 year randomized single-blind two arm intervention trial to compare the effects on mobility, activity and participation of a standard strength, endurance, and flexibility program to the standard plus timing and coordination program in 248 community-dwelling older adults walking slower than the desired gait speed of 1.2 m/s. Exercise sessions are twice weekly for 12 weeks. Participants are assessed at baseline, 12 weeks (post intervention), 24 weeks and 36 weeks. The primary, secondary, and tertiary outcomes are conducted by assessors masked to group assignment. The primary outcome is gait speed. Secondary outcomes of maximum voluntary isometric contraction of the quadriceps femoris, Six minute walk test, Chair Sit-and-Reach Test, Smoothness of walking, and Gait variability represent the components of the intervention (i.e. strength, endurance, flexibility and timing and coordination). Tertiary outcomes represent measures of activity (Late Life Function and Disability Instrument – function subscale and confidence in walking) and participation (Late Life Function and Disability Instrument – disability subscale and daily physical activity).

Interventions and Duration. Intervention sessions are twice weekly for 12 weeks supervised by a physical therapist. All sessions last about 50-65 minutes and include a warm-up, 40-55 minutes of exercise, and a cool down period. Both intervention groups will also receive physical activity behavioral change intervention. The Standard Intervention includes a brief warm-up period, lower extremity strength training, endurance training, and a brief cool down period. The Standard-Plus Task Specific Timing and Coordination Intervention or Standard-Plus intervention includes all components of the Standard Intervention plus task specific timing and coordination training. The total intervention time will be equal between the two intervention groups.

Sample Size and Population. The target sample is community-dwelling older adults who walk faster than 0.60 m/s and slower than the desired gait speed of 1.2 m/s. We plan to enroll approximately 124 subjects who walk slowly (i.e. gait speed > 0.60 and < 1.0 m/s) and approximately 124 faster walkers (i.e. gait speed ≥ 1.0 and < 1.20 m/s) for a total of 248 subjects. Within each baseline gait speed stratum we plan to randomize individuals to each of the treatment arms so that the gait speed strata are equally represented in each of the arms. Persons who are unable to participate in testing, have medical conditions which would make testing or participation in an exercise program unsafe, or who have plans to permanently leave the area during the study are excluded.

STUDY TEAM ROSTER

Principal Investigator:

Jennifer S. Brach, PhD, PT
Bridgeside Point 1
100 Technology Drive
Pittsburgh, PA 15219-3130
Phone: 412-383-6533
Fax: 412-648-5970
jbrach@pitt.edu
Main responsibilities/Key roles: Oversees and is responsible for all aspects of the study

Co-Investigators:

**Rakie Cham, PhD**<br>Department of Bioengineering<br>302 Benedum Hall<br>Pittsburgh, PA<br>(412) 624-7227<br>rcham@pitt.edu<br>Main responsibilities: Gait outcomes	**Andrea Kriska, PhD**<br>Department of Epidemiology<br>505B Public Health<br>Pittsburgh, PA<br>(412) 624-3996<br>aky@pitt.edu<br>Main responsibilities: GLB behavioral intervention
**Neelesh Nadkarni, MD, PhD**<br>Division of Geriatric Medicine<br>Kaufmann Building, Suite 500<br>Pittsburgh, PA<br>Phone: (412) 692-2383<br>nkn3@pitt.edu<br>Main responsibilities: Study physician	**Subashan Perera, PhD**<br>Division of Geriatric Medicine<br>Kaufmann Building, Suite 500<br>Pittsburgh, PA<br>Phone: (412) 692-2365<br>Ksp9@pitt.edu<br>Main responsibilities: Randomization, data management and study statistician
**Jessie VanSwearingen, PhD, PT**<br>Bridgeside Point 1<br>100 Technology Drive<br>Pittsburgh, PA 15219-3130<br>Phone: 412-383-6533<br>jessievs@pitt.edu<br>Main responsibilities/Key roles: Quality control of the intervention	

1 STUDY OBJECTIVES

1.1 Primary Objective

To determine if a standard-plus program is more successful in improving walking ability (i.e. gait speed) at 12 weeks after study entry compared to a standard exercise program. *Compared to participants receiving the standard program, participants receiving the standard-plus program will have greater gains in gait speed at 12 weeks after study entry (Hypothesis 1.1); and differences in gains between the programs will occur in both slower (<1.0 m/s)and faster (≥1.0 m/s) walkers (Hypothesis 1.2).*

1.2 Secondary Objectives

To assess the effect of the interventions on secondary and tertiary outcomes that represent the main components of the intervention and measures of activity and participation. *Compared to participants receiving the standard program, participants receiving the standard-plus program will have greater gains in timing and coordination (smoothness of walking, gait variability), activity (Late Life Function and Disability Instrument - function), and participation (Late Life Function and Disability Instrument-disability and physical activity as measured by Actigraph accelerometer) at 12 weeks after study entry; gains in strength (leg press strength and power), endurance (Six Minute Walk Test) and flexibility (Chair Sit-and-Reach) will be similar between the two active treatment groups.*

To determine if walking ability, secondary, and tertiary outcomes differ between standard-plus and standard intervention groups after a delay and are sustained over a period of time. *Compared to the participants receiving the standard program, participants receiving the standard-plus program will have greater gains at 24 (delayed effect) and 36 weeks (sustained effect) post randomization.*

To explore the effects of the interventions on outcomes within various other subgroups of interest (i.e. baseline confidence, physical activity and cognition subgroups).

2 BACKGROUND AND RATIONALE

Walking difficulty in older adults contributes to loss of independence, higher rates of morbidity and increased mortality.(1-5) Mobility loss is also a sentinel predictor of other disabilities that restrict independent living.(6) Compared to older adults without self-reported walking difficulty, those who developed mild walking difficulty over one year had higher healthcare costs (mean $1,128 per person). Extrapolated to the estimated 22% of older adults who develop walking difficulty annually, the cost to society is an additional 3.6 billion dollars per year.(7) Therefore, preventing or delaying the onset of walking difficulty might have a substantial impact on quality of life and healthcare costs of older adults.

2.1 Exercise for Health Promotion: Have we forgotten the nervous system?

Current exercise recommendations for older adults from the American College of Sports Medicine and the American Heart Association focus primarily on strength, endurance and flexibility training.(8, 9) Based on these exercise recommendations, the ongoing Lifestyle Interventions and Independence for Elders (LIFE) study examines a standard walking endurance, strength, static balance and flexibility intervention.(10) The LIFE pilot study, using the same intervention, demonstrated significant but *modest* effects.(11) For example, walking speed improved by only 0.01 m/s in the exercise group and decreased 0.02 m/s in the control group. The key question is: have we addressed all the most important contributors to walking ability? Might we be missing something?

Walking is a complex task that places demands on multiple systems including the musculoskeletal (muscles, bones, and joints), cardiopulmonary (heart and lungs), and nervous systems (brain, spinal cord and peripheral nerves).(12, 13) Current exercise recommendations target the musculoskeletal and cardiopulmonary systems and overlook the nervous system. A motor skill based exercise approach uses task specific timing and coordination exercises to challenge the brain to adapt and learn the sequence of movements and timing with the postures and phases of gait to improve walking. Improvements in walking occur by restoring the pattern of brain and neuromuscular activation that optimizes the use of capacities to meet the demands of the task of walking. Given the widespread subclinical and clinical neurological abnormalities with aging, it is time to incorporate explicit neurological training into exercise for older adults. Thus a potentially important but as yet unaddressed strategy to promote walking ability through exercise is to add training of timing and coordination in gait (i.e. task specific timing and coordination training) to usual strength, endurance, and flexibility training. Our preliminary data, presented below, suggests that task specific timing and coordination training has beneficial effects on mobility (gait speed) that are greater than a standard strength and endurance program.

2.2 Why would the standard-plus intervention impact activity and participation?

Walking underlies many activities of daily living and walking difficulty is associated with reduced activity and participation.(5, 14) The timing and coordination component of the standard-plus intervention, is a <u>task-oriented</u> approach which emphasizes the sequence of movements and timing with the postures and phases of gait to improve walking. The ultimate goal of the timing and coordination training is to make the older adult a skilled, “expert” walker. For motor tasks, expert movers, or those with greater motor skill for a specific activity, tire less easily than novices because of the greater efficiency of skilled motor performance.(15-17) Older adults who are “expert walkers” (i.e. skilled walkers) have efficient gait, tire less easily and as a result will likely walk more, participate in more activities and report less disability.(18)

2.3 Why would the benefits of the standard-plus intervention be sustained after the intervention has ended?

Intervention strategies challenge the brain to improve walking performance in different ways. An impairment-based intervention, such as the standard strength and endurance program, challenges the brain to use increased capacity in body systems to compensate for gait difficulties. Walking performance likely improves secondary to increased ability to produce muscle forces, to move joints through a greater range of motion, and to deliver more oxygenated blood to the active tissues. The use of greater capacity of body systems for walking makes the outcome of the impairment-based intervention approach potentially inefficient and difficult to sustain. A motor skill based approach challenges the brain to adapt and learn the sequence of movements and timing with the postures and phases of gait to improve walking. Improvements in walking occur by restoring the pattern of brain and neuromuscular activation that optimizes the use of capacities to meet the demands of the task of walking. The task-oriented focus of the motor skill based approach has the potential to lead to not only an efficient and automatic motor sequence pattern for walking, but also reward-based adaptive changes in the brain which may be sustainable. Recently, in a randomized controlled trial comparing strength exercises to task-oriented exercises in older women, improvements in functional task performance were sustained for six months after the end of training only in the task-oriented group.(19)

2.4 Study Rationale

Rationale for Interventions

Standard impairment-based strength and endurance interventions, which ignore the timing and coordination of movement, have a real but modest impact on walking ability in older adults.(10, 20-25) Aging and disease alter timing and coordination as reflected by slowed neuromotor performance, increased gait variability and reduced smoothness of movement.(26-29) A task specific timing and coordination intervention that includes practice of smooth coordinated aspects of gait timing over multiple walking conditions has the potential to improve walking ability greater than a standard program.(30)

We conducted two pilot studies, involving contrasting subject groups, to examine the short-term impact of a timing and coordination exercise program on walking. The first study (RESTORE) included older adults with slow (gait speed < 1.0 m/s) and variable gait and has been published.(30) The second study (PRIME) has recently been completed.(31) It included older adults with near normal gait speed (gait speed > 1.0 m/s) but with difficulty with aspects of the timing and coordination of walking (i.e. Figure of 8 test time > 8.0 see measures section below for details of this measure). Participant retention for the 12 week posttests was over 95%.

In the RESTORE study, 50 subjects (mean age 77.2±5.5 years, 65% women) were randomly assigned to either a standard exercise program (endurance, strength and typical static balance training) or a timing and coordination program, for one hour, 2

times per week for 12 weeks, with baseline and 12 week follow up assessments. Of the 50 who entered, 47 (94%) completed the study. Both groups increased gait speed (timing and coordination by 0.21 m/s and standard by 0.14 m/s); adjusted group difference 0.07 m/s, p=0.10. The timing and coordination group had a 3.5±1.7 (p=0.04) point greater gain than the standard group in basic lower extremity function (LLFDI) and a 2.6±1.7 (p=0.12) point greater gain than the standard group in advanced LE function (LLFDI).(32) Note that this pilot targets a population similar to that proposed here but that the treatment arms differ from the current proposal in that the timing and coordination group did not receive endurance training.

In the PRIME study, 38 subjects (mean age 78.5±5.6 years, 65% women) were randomly assigned to either a standard endurance and strength exercise program or a timing and coordination plus strengthening program, 2 times per week for 12 weeks, with assessments at baseline and immediately following the 12 week intervention. Preliminary analyses indicate that the timing and coordination group had greater improvements in gait speed than the standard group (adjusted group difference 0.11 m/s, p=0.008). Both groups had improvements in endurance (6MWT: p<0.05) and the timing and coordination group had greater improvements in the timing and coordination of walking (Smoothness of walking HR_{AP} adjusted group difference = 0.53, p=0.05 and Figure of 8 test time adjusted group difference = -1.39s, p<0.0001). The timing and coordination group reported increased participation (LLFDI-disability limitation increased 6.0 points, p=0.05; however, neither group improved on activity measures (LLFDI–function) most likely due to the high baseline values and potential ceiling effect. The percent of time spent in sedentary behavior did not differ significantly between the groups immediately following the intervention (adjusted group difference -1.7%, p=0.27). We hypothesize that changes in daily activity will likely be delayed, in that subjects need time to adjust to their improved walking prior to increasing their daily activity.(31, 33)

Figure 1 presents the changes in gait speed achieved in the LIFE-P study, and our two

Figure1. Changes in gait speed with standard and timing and coordination (T&C) training.

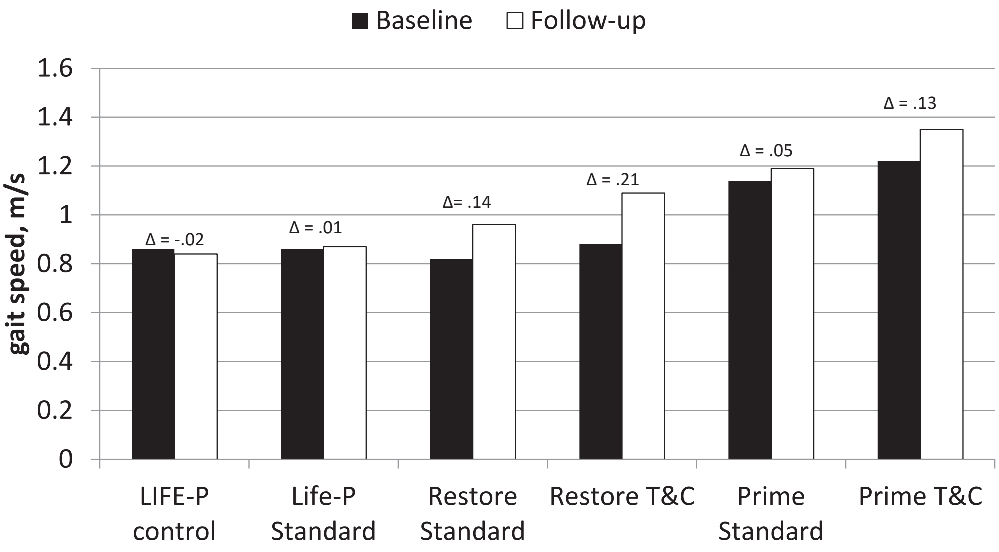

pilot studies (RESTORE and PRIME). Improvements in gait speed were modest in LIFE-P and the standard program groups of RESTORE and PRIME and much larger in the timing and coordination groups. Note improvements in gait speed with timing and coordination training were evident in both slower (RESTORE) and faster (PRIME) walkers. Though the overall improvements in gait speed with timing and coordination training were greater for the slower walkers in RESTORE, adjusted group differences in gait speed were larger for the faster walkers in PRIME (PRIME group difference = 0.11m/s, SE=0.04; p=0.008 and RESTORE group difference = 0.07m/s, SE=0.04; p=0.10). Individuals with gait speed > 1.0 m/s did not benefit as much from the standard intervention compared to the task specific timing and coordination training (i.e. gait speed increased 0.05 m/s for the standard training and 0.13 m/s for the task specific timing and coordination training), suggesting that task specific timing and coordination may play a critical role in improving mobility in older adults with near normal gait speed (i.e. > 1.0 m/s).

3 STUDY DESIGN

3.1 Overview and Study Design

A randomized single-blind two arm intervention trial of 248 community-dwelling older adults walking slower than the desired gait speed of 1.2 m/s will be carried out at the University of Pittsburgh. Half of the participants will receive the standard strength, endurance, and flexibility program (Standard) and the other half will receive the standard plus timing and coordination program (Standard plus). Both groups will receive 16 sessions of behavioral interventions to promote physical activity. Potential participants will be screened over the phone to determine initial eligibility and then will be scheduled for a clinical screening visit. Prior to the clinic screening visit, the participant's physician will be contacted and clearance to participate in a moderate intensity exercise program will be obtained. At the clinic screening visit, written informed consent will be obtained and the screening examination will occur to determine final eligibility. Eligible participants (i.e. those who meet all inclusion and exclusion

Figure 1. Timeline for recruitment, randomization, and follow-up.

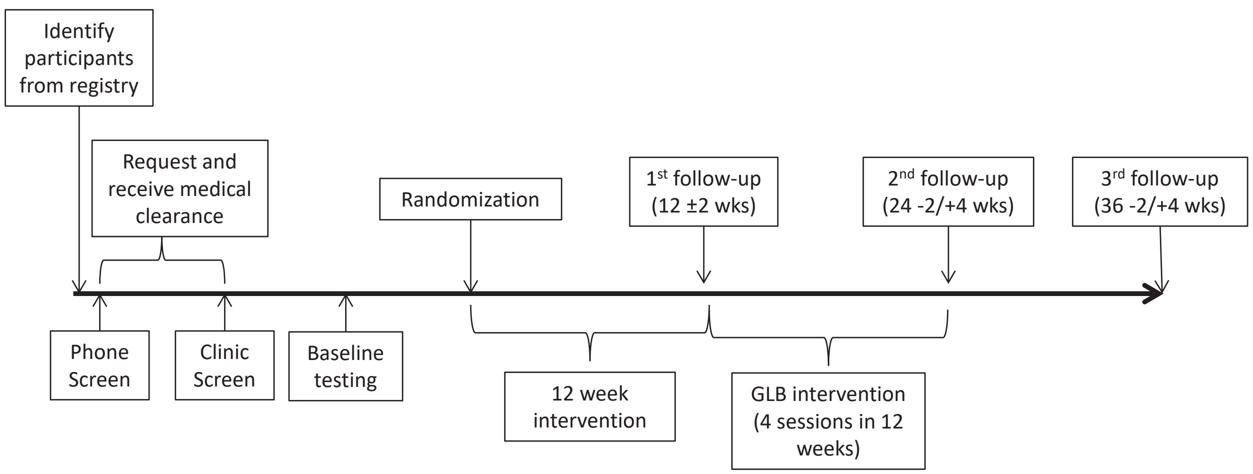

criteria) will undergo a comprehensive baseline assessment and will then be randomized to one of the two treatment groups. Assessments will be collected again at 12 weeks (post intervention), 24 weeks and 36 weeks post randomization. Figure 1 shows the sequence of participant contacts from the time of initial contact to the final assessment approximately 36 weeks after randomization.

4 SELECTION AND ENROLLMENT OF PARTICIPANTS

Candidates will be recruited through the Pittsburgh Pepper Center Registry. Screening will be conducted in 2 phases (phone and clinic). Initial phone contact uses a structured questionnaire to identify inclusion and exclusion criteria by self-report. During the phone screen, we will obtain the name and phone number of the participant's primary care physician in order to obtain medical clearance for participation in the exercise

intervention. Medical clearance for participation in the exercise intervention must be obtained prior to the onsite clinical examination. Individuals who meet criteria on the phone screen will be scheduled for an onsite clinic screen to identify additional potential exclusions. Prior to initiating the onsite examination, informed consent will be obtained. To determine eligibility based on gait speed, gait speed will be measured using a timed 4 meter corridor walk. We plan to enroll 124 subjects who walk slowly (i.e. gait speed > 0.60 and < 1.0 m/s) and 124 faster walkers (i.e. gait speed ≥ 1.0 and < 1.20 m/s) for a total of 248 subjects. The phone and clinic screening are described in greater detail in section 6.2.1 below.

4.1 Inclusion Criteria

Participants must meet all of the following inclusion criteria to participate in this study. Inclusion criteria include the following:

1. 65 years of age and older
2. Ambulatory without an assistive device or the assistance of another person
3. Usual 4 meter gait speed > 0.60 m/s and < 1.2 m/s
4. Physician clearance to participate in a moderate intensity exercise program

4.2 Exclusion Criteria

All candidates meeting any of the exclusion criteria at time of screening will be excluded from study participation. Exclusion criteria include the following:

1. Inability to participate in testing or exercise intervention:
    a. Persistent lower extremity pain that is present on most days of the week that would interfere with participation in the exercise program.
    b. back pain that is present on most days of the weeks and interferes with walking and activities of daily living or back pain that increases with walking (lumbar stenosis)
    c. calf pain or cramping which worsens with walking and is relieved by rest (PAD)
    d. refuse to walk on a treadmill
    e. plans to move out of the area in the next 5 years
2. Safety concerns:
    a. dyspnea at rest or during activities of daily living or use supplemental oxygen (CHF, COPD)
    b. any acute illness or medical condition that is not stable according to the approving physician
    c. resting systolic blood pressure ≥ 200 mm Hg or diastolic blood pressure ≥ 100 mm Hg or resting heart rate > 100 or < 40 beats per minute[71]

d. diagnosed dementia or cognitive impairment defined as 3MS<79
e. hospitalized in the past 6 months for acute illness or surgery, other than minor surgical procedures
f. severe visual impairment as indicated by difficulty navigating in the clinic
g. fixed or fused lower extremity joints such as hip, knee or ankle
h. lower extremity strength <3/5 on manual muscle testing
i. lower extremity amputation
j. progressive movement disorder such as MS, ALS or Parkinson's disease

4.3 Study Enrollment Procedures

4.3.1 Identifying and Recruiting Participants

Candidates will be recruited through the Pittsburgh Pepper Center Registry of over 2200 older adults who have signed consents to be directly contacted by participating researchers about mobility research studies. This sample is diverse in gender, age and ethnicity and has a wide range of self-reported mobility. Potential participants from the registry will be sent a letter and asked to contact the study coordinator by phone. If the potential participant does not respond within 2 weeks we will contact them by phone and ask if they would like to hear more about the study.

During the initial phone call, potential participants will be informed of the nature of the screening questions. They will also be informed that their answers to the questions will be stored confidentially. Additionally, they will be reminded that their participation in the phone screening is strictly voluntary. The participant's verbal consent will be noted on the telephone screening form. If the potential participant declines the telephone interview, the form will be destroyed. If the after the phone interview the potential participant is ineligible, the reason for ineligibility will be documented on the screening log. If after the phone interview the participant is eligible we will obtain the name and phone number for the primary care physician so that medical clearance can be obtained. They will then be scheduled for a clinic screen visit and notified of the date, time and location of the visit. Written informed consent will be obtained prior to conducting the clinic screen. Details of the clinic screen, baseline testing and randomization can be found below in section 6.2.

5 <u>STUDY INTERVENTIONS</u>

5.1 Overview

Participants will be assigned to one of two treatment groups: 1) Standard or 2) Standard-Plus. The Standard intervention group will receive strength, endurance and flexibility training and the Standard-Plus intervention group will receive strength, endurance, flexibility and timing and coordination training. Both groups will receive 24

sessions approximately 60 minutes in duration from a study PT. Participants will receive 2 sessions per week on non-consecutive days for 12 weeks. Training sessions will occur at the Physical Therapy Clinical and Translational Research Center (PT-CTRC).

5.2 Blinding and Study Staff

Given the nature of the interventions being tested in this study, it will not be possible to blind the participants or the PT providing the interventions. To minimize "contamination" of the interventions, participants receiving different interventions will not be scheduled during the same time at the PT-CTRC. Study staff involved in the randomization or intervention will not perform follow-up data collection. Study staff performing the follow-up assessments will be blinded to treatment assignments and will be asked not to seek this information when in contact with the participants. Participants will be asked not to discuss their treatment experience during study visits. Only a small number of staff members and members of the DSMB will see group-specific study results.

5.3 Group Lifestyle Balance™ (GLB) - Behavioral Intervention

Both intervention groups will also receive a physical activity behavioral change intervention based on the Group Lifestyle Balance™ (GLB) program. The GLB program is a behavioral lifestyle intervention which is modeled closely on the original and highly successful Diabetes Prevention Program (DPP). The DPP is a NIH funded study that demonstrated that small changes in lifestyle such as healthy eating and increased physical activity could lower the risk of developing type 2 diabetes in those at high risk for the disease. The GLB was designed to help individuals reach a balance between two parts of their lifestyle, eating and physical activity.(34)

We will primarily focus on the physical activity component of the program. The physical activity goal of the GLB program is for participants to achieve and maintain a physical activity level of at least 150 minutes each week of moderate intensity activity similar to a brisk walk. Participants in the Standard and Standard-plus interventions will participate in 16 GLB sessions. The first 12 sessions will be weekly, individual sessions. The final 4 sessions will be delivered in a small group setting over a 12 week time period as follows: every other week for 4 weeks (2 sessions), once every 4 weeks for 8 weeks (2 sessions). Sessions cover topics such as "Jump Start Your Activity Plan", "Problem Solving", "Ways to Stay Motivated", "Stress and Time Management", etc. All sessions are thoroughly described in the behavioral intervention manual.

5.4 Standard Intervention (include progression and modifications)

The Standard Intervention includes a brief warm-up period (5 minutes), lower extremity strength training (10-15 minutes), endurance training (30-40 minutes), and a brief cool down period (5 minutes) for a total of 50 to 65 minutes of intervention. The warm-up and cool down periods contain gentle stretches for the lower extremity and trunk. The strength training is conducted on Magnum stacked weight training equipment (leg

extension/ curl combo, leg press machine, and multi-hip combo) and can include the following exercises: knee extension, knee flexion, leg press, hip abduction, hip extension, etc. When subjects are able to complete 2 sets of 15 repetitions with minimal effort (i.e. RPE < 10), resistance is increased for progression of the exercises. Subjects will always complete hip extension and hip abduction strengthening exercises as well as 1-2 other lower extremity strengthening exercises based on individual needs determined by the supervising physical therapist. The endurance training consists of treadmill walking at a submaximal workload with a self-reported rating of perceived exertion (RPE) of 10-13, somewhat hard. When subjects are able to tolerate a 10-13 RPE level for 15 minutes, the workload is increased. The goal is to achieve 40 minutes of continuous treadmill walking exercise at a somewhat hard level of exertion. For safety, participants are told they should stop walking immediately if they feel they cannot continue (symptom-limited), if they or the PT observes shortness of breath, if they demonstrate problems in the walking pattern (e.g. toe drags on the floor during the swing-through phase of gait), or they report or the physical therapist observes any of the general indications for stopping nondiagnostic exercise tests as recommended by ACSM (e.g. ACSM Exercise Guidelines, Table 4-6, p78).(35) A complete description of the standard program, including progression of exercises, can be found in the MOP.

5.5 Standard-Plus Intervention (include progression and modifications)

In addition to the Standard Intervention described above the subjects receiving the Standard-Plus Intervention will complete task specific timing and coordination training. In order to keep the total intervention time equal and the time spent in walking/standing activities equal, the subjects in the Standard Plus intervention complete a 5 minute warm-up, 10-15 minutes of lower extremity strength training, 15-20 minutes of endurance training, 15-20 minutes of timing and coordination training and a 5 minute cool-down for a total of 50-65 minutes of intervention. The timing and coordination training is based on principles of motor learning that enhance "skill" or smooth and automatic movement control.(16, 36-40) This program uses goal-oriented progressively more difficult stepping and walking patterns to promote the timing and coordination of stepping, integrated with the phases of the gait cycle.(16, 37, 38, 40) Conceptually, the exercise is intended to achieve its effects by shifting the center of pressure posteriolateral then forward, encouraging hip extension prior to stepping, loading the trailing limb, coordinating activation of the abductors of the soon to be swing leg with adductors of the stance limb, and shifting the center of pressure in medial stance to unload the stepping limb.(41-43) Progression is based on first separately increasing the speed, amplitude or accuracy of performance prior to undertaking a more complex task.(44) For example, the progression of stepping patterns is, 1) self-paced step forward and across, 2) increase stepping speed, 3) alternate side of stepping, 4) alternate forward with backward stepping. Walking patterns incorporate patterns of muscle coordination and interlimb timing similar to the stepping exercises into walking. Walking patterns progress by separately altering speed, amplitude (e.g. narrowing oval width), or accuracy of performance (e.g. without straying from the desired path), and then to complex walking patterns involving walking past others and with upper extremity object manipulation tasks added, such as carrying, bouncing or tossing a ball.(40) Timing and coordination training was used in both of our pilot studies.(30, 31) The

standard impairment-based intervention aims to increase physiologic capacity in body systems that contribute to walking, but does not include task specific exercise necessary to make use of the physiological capacity in body systems (i.e. musculoskeletal and cardiopulmonary systems) for the walking. Whereas, the task specific timing and coordination intervention aims to improve the motor skill of walking by re-aligning biomechanical and neuromotor programs, and improving feedback for adjusting movements. In a sense, an impairment-based intervention is comparable to "building a bigger engine" whereas the task specific timing and coordination intervention is comparable to a "tune-up" of an engine for optimal performance.(45) A complete description of the timing and coordination program, including progression of exercises, can be found in the MOP.

5.6 Treatment Fidelity Plan

5.6.1 Initial Training for Procedural Reliability

Dr. VanSwearingen and Dr. Brach will train the PTs in both the Standard and the Standard-plus interventions. Knowledge of procedures will be tested by psychomotor skills observation. Dr. VanSwearingen and Dr. Brach will document that the PTs are "PRIMA" certified PTs and provide a certificate of completion after they demonstrate competence with the interventions. All PT-CTRC PTs are CPR certified and must maintain this certification throughout the study. As new PTs join the study, training will be conducted on an individualized basis following the same procedures.

5.6.2 Ensuring Ongoing Competency

We will use a multi-faceted approach to ensure ongoing treatment fidelity. The approaches include direct observation of skills, mandatory monthly telephone calls with all treating PTs, periodic review of intervention data sheets, and face-to-face meetings with the intervention team at least once per year.

<u>Direct Observation of Skills</u>: Direct observation of the PTs as they perform the interventions will be conducted by Dr. VanSwearingen or a senior research physical therapist trained in the intervention. The evaluators will document their observations using a structured checklist that addresses data completeness, physical performance, qualitative observations, and verbal and nonverbal communication. In the Standard intervention the checklist will also document whether the participant completed 5 minutes of warm-up, 10-15 minutes of lower extremity strength training consisting of exercises that address at least 3 muscle groups, 30-40 minutes total of endurance training at an RPE of 10-13, and a 5 minute cool down. In the Standard-plus intervention the checklist will also document whether the participant completed 5 minutes of warm-up, 10-15 minutes of lower extremity strength training consisting of exercises that address at least 3 muscle groups, 15-20 minutes of endurance training at an RPE of 10-13, 15-20 minutes of timing and coordination training and a 5 minute cool down. There will be two observations per quarter for each intervention for new PTs and one observation for each intervention per quarter for PTs who have been with the study more than 6 months. If the total score on the checklist is less than 90% a remediation plan will be developed that will include refresher training to ensure adequate understanding of the protocol and follow-up observation visits.

Mandatory monthly check ins: Dr. VanSwearinegen will conduct monthly check ins with the PTs delivering the intervention. The study coordinator be included in the phone call or email exchange and will document minutes and ongoing training where applicable. During these check ins, the PTs will be able to ask questions about the intervention delivery. The check ins will provide the PTs with an opportunity to brainstorm and problem solve with Dr. VanSwearingen.

Periodic Review of Intervention data sheets: Intervention data sheets will be reviewed periodically to ensure the proper intervention is being delivered and that the intervention is being progressed over time. Review of intervention data sheets will occur at the completion of the on-site intervention. Initially, intervention data sheets from two of the first 5 participants in each intervention arm will be reviewed. Once the initial reviews are completed, subsequent reviews will occur quarterly (i.e. two participants in each intervention). If the documentation of the intervention and/or the progression of the intervention is inadequate, a remediation plan will be developed that will include refresher training to ensure adequate understanding of the protocol and follow-up observation visits.

Face-to-face meetings: Dr. VanSwearingen will visit the PT-CTRC semi-annually to meet with the PTs delivering the interventions. This will be a team building and problem solving visit emphasizing proper delivery of the intervention and discussing issues specific to the site.

5.7 Scheduling Intervention Visits

There are several requirements for scheduling of intervention visits:

1. The first intervention visit should be scheduled 8-14 days after baseline testing is completed. Randomization is to be completed 1-2 business days before first intervention visit OR 14 days after baseline testing is completed.

2. The general schedule of visits includes 2 visits per week for 12 weeks for a total of 24 visits. The goal is to complete 24 visits. Missed visits because of illness, holidays, or vacations can be rescheduled in the following weeks; however, all visits must occur within a 14 week time period.

3. Intervention visits should never occur on two consecutive days.

5.8 Adherence Assessment

Adherence to the intervention will be defined as the number of intervention sessions completed. Adherence to the exercise intervention (standard or standard-plus) will be tracked separately from the adherence to the behavioral intervention. The staff

delivering the intervention will track and record the attendance of the participant in the exercise intervention and the behavioral intervention on the adherence log form. At the completion of the intervention, the final total attendance will be entered into the database.

6 STUDY PROCEDURES

6.1 Schedule of Evaluations

Assessment	Phone Screen	Clinic Screen	Baseline	12 week	24 week	36 week	Each intervention visit	As needed
Phone Screen	X							
Informed Consent Form		X						
Demographics Questionnaire		X						
Physical Exam		X						
Comorbidity Index		X						
Anthropometric Measures		X		X	X	X		
Gait speed screen		X						
Modified Mini-Mental State (3MS) Test		X						
Medical clearance to participate		X						
Fall history questionnaire			X	X	X	X		
Gait Assessment Walkway			X	X	X	X		
Leg strength and power			X	X	X	X		
Six minute walk test			X	X	X	X		
Chair sit and reach			X	X	X	X		
Smoothness of walking			X	X	X	X		
Gait Efficiency			X	X	X	X		
Figure of 8			X	X	X	X		
Gait Efficacy Scale			X	X	X	X		
Late Life Function and Disability Index			X	X	X	X		
Life Space Assessment			X	X	X	X		

Pitt Sleep Quality Index			X	X	X	X		
Geriatric Depression Scale			X	X	X	X		
Actigraph Diary			X	X	X	X		
Exercise adherence log							X	
Adverse events				X	X	X		X
Change in status								X
Health status update								X
Medical clearance to return after illness or event								X
Protocol deviation								X

6.2 Description of Evaluations

6.2.1 Screening (Phone and Clinic)

Screening will be conducted in two phases, phone and clinic. Initial phone contact uses a structured questionnaire to identify inclusion and exclusion by self-report. Individuals who meet the criteria will be scheduled for an onsite examination (clinic screen) to identify additional potential exclusions.

Consenting Procedure

Prior to the phone screen we will obtain verbal consent. During the initial telephone call, potential participants will be informed of the nature of the screening questions. They will also be informed that their answers to the questions will be stored confidentially. Additionally, they will be reminded that participation in the telephone screening is voluntary. The participant's verbal consent will be noted on the telephone screening form. If the potential participant declines the telephone interview, the form will be destroyed.

Medical Clearance. Once the participant passes the phone screen, we will obtain the name and phone number of the participant's primary care physician so that we can obtain medical clearance for participation in the study. The physician's office will be contacted and the fax number obtained. We will fax the physician's office a letter and a form for medical clearance and ask that it be completed and returned to us by mail or fax. Medical clearance must be obtained prior to the clinic visit for screening and baseline testing.

Prior to performing any of the clinic screen measures, written informed consent will be obtained. During the clinic screen visit, the participant will be given the consent form to read. The consent form will then be reviewed in the presence of one of the study investigators. The investigator will then answer any questions that the participant may have about the study. The investigator will ask the participant several questions about the study to make sure they understand the study procedures. Finally the participant will be asked to sign the consent form. The signed consent form will be stored in the participant's research record in a locked file cabinet. The participant will be given a copy of the consent form for their records.

Clinic Screen

The clinic screen will include the following measures:

- Demographic questionnaire
- Physical Exam
- Comorbidity Index
- Anthropometirc measurements
- Gait speed – 4 meter walk

- Modified Mini-Mental State (3MS) test

All screening measures will be completed prior to determining subject eligibility. Once all measures are collected, the values will be reviewed to determine eligibility. Participants will be informed at the clinic screen visit of their eligibility. Participants who are not eligible will terminate participation at the end of the clinic screen. Participants who are eligible will continue on with baseline testing. All screening tests must be measured within 14 days of randomization.

6.2.2 Baseline

Baseline assessment will occur immediately following the clinic screen and will include the following measures:

Mobility Outcome (Primary Outcome):
Gait Speed - The primary mobility outcome is gait speed. We selected gait speed as our primary outcome because of its strong psychometric properties, its significant association with morbidity and mortality, and the continuous nature of the variable. Gait speed is a reliable,(46, 47) valid, sensitive(11) and specific(48) measure that correlates with functional ability and balance confidence. Gait speed is strongly association with future disability, falls, hospitalization, nursing home admission and mortality.(1-5) Gait speed is a responsive measure that can assess change over time(11, 49) thus an improvement in gait speed is likely a good indicator of the effectiveness of an exercise program. In addition, gait speed was chosen by a panel of experts as the standardized assessment to measure locomotion for the Motor Function Domain of the NIH Toolbox.

Gait speed is assessed in usual walking with a computerized walkway. After explanation and demonstration, the participant completes a practice walk the length of the walkway to become accustomed to walking on the mat. Each walk is considered one pass. The subject then completes 6 passes on the walkway at their usual, self-selected walking speed. Gait speed will be averaged over the 4 passes. The test-retest reliability of gait speed measured using a computerized walkway by ICC is 0.98.(50) A higher speed is better.

Outcomes representing components of the interventions (Secondary Outcomes):
In order to assess the integrity of the intervention, we selected measures that represent the main components of the intervention (i.e. strength, endurance, flexibility, and timing and coordination) as our secondary outcomes. Measures representing the underlying components of the interventions are:

Lower Extremity Strength and Power - Leg strength will be measured by determining the 1 repetition maximum (1RM) for each leg individually using a Keiser A420 electronic pneumatic leg press machine. We will follow the protocol previously described by Callahan et al, 2007.(51) The maximum value observed on the A420 graphical display of either side will be recorded as the peak leg strength. Leg press power will also be

measured using the Keiser A420 leg press machine. Leg press power will be measured as the peak power graphically recorded when the individual performs a single leg press repetition pushing out as quickly as possible at 40% and 70% of 1RM. Five repetitions will be recorded for each leg at each resistance. The highest recorded power of all repetitions (either side, either resistance) will be recorded as leg press power.

Six Minute Walk Test (6MWT) - The Six Minute Walk Test (6MWT) of distance walked (meters) in six minutes, including time for rest as needed,(52) will be used to determine the impact of the exercise interventions on endurance. The 6MWT has been used to describe and monitor endurance capacity.(52) The 6MWT has established psychometric properties, test-retest reliability (Pearson r=.95) in older adults,(48, 53) construct validity for graded exercise test and functional classification.(54) We chose the 6MWT because it is responsive to change,(55) widely used, and included in the NIH, PROMIS project to establish measures for clinical assessment. Greater distance covered during six minutes is better.

Chair Sit-and-Reach Test - The Chair Sit-and-Reach Test, a measure of hamstring flexibility, will be used as the main measure of flexibility.(56) Subjects sit on the edge of a chair with their preferred leg extended and the other leg bent with the foot flat on the floor. Subjects bend forward at the hips, keeping the spine as straight as possible. The distance reached in relation to the foot is recorded in cm. The Chair Sit-and Reach test was selected as the measure of flexibility because it does not require the older adult to sit on the floor, which may be difficult for some. The test has established psychometric properties of test-retest reliability (ICC=0.92-0.96) and criterion validity with comparison to the sit-and-reach test (r=0.74). Values can be positive or negative with positive numbers indicating greater flexibility.

Smoothness of walking – Smoothness of Walking is an integrated measure of motor control of walking,(57, 58) and is assessed during usual over ground walking. Linear acceleration of the body is measured along three axes (vertical, anterior-posterior and medial-lateral) using a tri-axial accelerometer attached to the skin over the L3 segment of the lumbar spine. Trunk accelerations are sampled at 200 Hz and are used to calculate the harmonic ratio following the methodology of Menz.(57) The harmonic ratio (HR) is derived in the vertical, medial-lateral and anterior-posterior directions. Higher HRs are better.

Gait variability – Gait variability, defined as fluctuations in gait characteristics from one step to the next,(59) is an important indicator of impaired mobility in older adults and will serve as a second indicator of timing and coordination.(60) Gait variability is quantified using established measures of temporal and spatial gait characteristics including stance time, step length, and step width. Variability will be calculated as the standard deviation of the set of steps recorded over 4 passes on the GaitMat (described above). Approximately 32 steps will be collected from 4 passes on the GaitMat which will be more than adequate to achieve a stable measure of gait variability. Our prior work has shown that 20 steps are sufficient to achieve a reliability of 0.75 and 30 are sufficient for 0.80.(61) In general, lower variability is better although there are exceptions.(60, 62)

Gait Efficiency - We will use the energy cost of walking as our indicator of gait efficiency. The energy cost of walking reflects the energy used for all bodily actions

during walking.(63) Subjects walk on a treadmill at a self-selected pace while oxygen consumption data is collected using open circuit spirometry and analysis of expired gases with a VO2000 portable metabolic measurement system, Medgraphics®, Minneapolis, MN. All subjects are familiarized with treadmill walking until comfortable walking on the treadmill, prior to the baseline measurement. The mean rate of oxygen and carbon dioxide consumption is determined over three minutes after reaching steady state.(63, 64) The energy cost of walking reported in ml/kg/m, represents an estimate of energy expenditure per unit of gait speed,(65-67) and relates to metabolic equivalents (METS). It is time independent, repeatable, reflects the physiological cost of gait,(63, 64) is little influenced by fitness,(63) and can be compared across individuals and over time, regardless of changes in gait speed.(63, 65) Lower cost is better.

Activity Outcomes (Tertiary Outcomes):
Using the World Health Organization's International Classification of Functioning, Disability and Health (ICF)(68) model to inform our selection of tertiary outcomes for this study, we will evaluate measures of activity (execution of a physical task).

Activity levels will be assessed with:
The Late Life Function and Disability Instrument (LLFDI-F).(14, 69) The LLFDI-F is our main activity outcome. The LLFDI-F is a self-report measure for assessing physical function in older adults with acute or chronic problems, and designed to be more sensitive to change than similar measures. The LLFDI-F has 32 items in three dimensions, basic lower extremity (BLE), advance lower extremity (ALE) and upper extremity (UE). We've selected the LLFDI-F because 1) it includes a wide variety of life tasks in various social areas thus extending beyond the traditional focus of just activities of daily living, 2) the scale was designed with sufficient breadth of items and increments of rating in order to minimize ceiling and floor effects and maximize the scale's ability to detect change over time, and 3) it is a continuous outcome which gives us greater power than a dichotomous outcome to detect change over time. We will focus our analyses on the LLFDI-F dimension scores (i.e. BLE function, ALE function, UE function). The LLFDI-F has established known groups validity and the test-retest reliability is extremely high for the function component (ICCs range from 0.91-0.98 for the dimensions). Scores range from 0-100; higher scores represent less difficulty.

Participation Outcomes (Tertiary):
Using the ICF(68) model to inform our selection of disability outcomes for this study, we will evaluate measures of participation (involvement in life situations). Participation levels will be assessed with:

The Late Life Function and Disability Instrument (LLFDI-D).(69) The LLFDI-D is our main participation outcome. The LLFDI-D is a self-report measure for assessing disability in older adults with acute or chronic problems, and designed to be more sensitive to change than similar measures. The LLFDI-D component has 16 items representing two dimensions, frequency of performance and limitation in performance of life tasks. We will focus our analyses on the LLFDI-D dimension scores (i.e. disability frequency and disability limitation). We will also examine the disability domain scores

(social role, personal role, instrumental role and management role) since they may provide insight into the impact of the disability on frequency of performance and perceived limitations.(69) The LLFDI-D scales have established known groups validity and the test-retest reliability is moderate to high (ICCs range from 0.68 to 0.82). Scores range from 0-100; higher scores represent less disability.

The Actigraph Accelerometer will be used to assess the participants' usual daily physical activity. The ActiGraph is often used in physical activity research and has recently been shown to be more reliable than other devices.(70) Accelerometers are electronic sensors that measure the quantity and intensity of ambulatory movement.(71) Due to the small size and weight of the monitor, the participant is not expected to experience a disruption in their gait or balance. The Actigraph Accelerometer will be worn on the waist during waking hours for 7 consecutive days, and the participants will be instructed to remove the monitor only for sleeping, during imaging studies, and or during swimming and bathing activities. For pre-intervention physical activity assessment the subject will be given the accelerometer during the baseline assessment. They will be asked to return the accelerometer during their first intervention visit. For post-intervention testing, the subject will receive the accelerometer during the last intervention visit and will be asked to return it during the post-intervention assessment. For the 24 and 36 week follow-ups the participant will be given the accelerometer during their visit and they will be asked to return it by mail in a postage paid envelope. We used the same methodology (returning by mail) in a past study of 120 older adults and received accelerometers back from > 95% of the subjects. The continuous data that are available through actigraphy provide many possible measures for analysis. We focus on 1) total activity counts, 2) percentage of time spent in sedentary activity and 3) number of bouts of moderate intensity activity as our outcomes.

Potential covariates

Trail Making Parts A&B – The Trail Making Test is a widely used test of executive cognitive function(72) that involves multiple cognitive domains and is administered in two parts. Completion of the Trail Making Test A (TMT-A) involves complex visual scanning, motor speed, and agility as participants draw lines to connect consecutively numbered circles as quickly as possible. Completion of Trail Making Test B (TMT-B) requires the additional processes of cognitive flexibility and set shifting as participants connect circles in an alternating sequence of numbers and letters, linking them in ascending order as fast as possible. Time to complete and number of errors for each portion will be recorded. Lower scores (faster times) indicate better performance.

Geriatric Depression Scale (GDS)(73) – The Geriatric Depression Scale has been used extensively in community-dwelling healthy and medically ill older adults to screen for depression. The short form GDS consists of 15 yes or no questions. Scores of 0-4 are considered normal, scores of 5-8 indicate mild depression, scores of 9-11 indicate moderate depression and scores of 12-15 indicate severe depression.

Pittsburgh Sleep Quality Index(74) – The Pittsburgh Sleep Quality Index is a self-rated questionnaire which assess sleep quality and disturbances over a 1-month time period.

Nineteen individual items generate seven “component” scores: subjective sleep quality, sleep latency, sleep duration, habitual sleep efficiency, sleep disturbances, use of sleeping medication and daytime dysfunction. The sum of the scores for the seven components yields a global score.

Life Space Mobility(75) – The University of Alabama at Birmingham (UAB) Study of Aging Life-Space Assessment (LSA), measures a person’s usual pattern of mobility in the previous month. Life-space can be visualized as a pattern of areas defined by distance extending from the location where a person sleeps. The LSA allows for a range of mobility assessment from limited to the room where the person sleeps with assistance from another person to independently traveling out of town. The LSA documents mobility based on how far and how often a person travels and the amount of assistance that is needed. Scores range from 0 (totally bed-bound) to 120 (travelled out of town every day without assistance). Higher scores indicate a greater mobility.

Gait Efficacy Scale. In order to determine if changes in walking difficulty are associated with changes in confidence in walking, confidence will be assessed using the Gait Efficacy Scale.(76) The items include a range of gait activities such as walking over different surfaces, up and down curbs, and negotiating stairs. Each item has a 10 point Likert scale scoring option, with the total score for the 10 items, ranging from 0-100. A higher score represents greater confidence.

6.2.3 Follow-up Visits

Follow-up visits will occur at 12 (conclusion of the intervention), 24 and 36 weeks post randomization. Windows for follow-up testing are as follows: 12±2weeks, 24 -2/+4 weeks, and 36 -2/+4 weeks. Measurements will include the following:

12, 24, and 36 weeks:

- Gait Speed
- Lower extremity strength and power
- Six minute walk test
- Chair sit and reach test
- Smoothness of walking
- Gait Efficiency
- Figure of 8 walk test
- Gait Efficacy Scale

- LLFDI
- Physical Activity – Actigraph
- Trail Making Parts A&B
- Geriatric Depression Scale
- Pittsburgh Sleep Quality Index
- Life Space Assessment

7 SAFETY ASSESSMENTS

7.1 Participant Safety Parameters: Methods and Timing

7.1.1 Screening

Potential participants will be excluded during the screening phases if they have persistent pain (lower extremity or back) that would interfere with participation or if they are medically unstable or have medical conditions which may impact their safety (see exclusion criteria). Individuals with resting systolic blood pressure ≥200 mm Hg or diastolic blood pressure ≥100 mm Hg or resting hear rate > 100 or < 40 beats per minute will also be excluded. All participants must also have physician clearance to participate in a moderate intensity exercise program prior to the start of the exercise intervention.

7.1.2 Safety Considerations for Assessments and Interventions

Expected Adverse Experiences

1. Major risks such as a cardiac event or a fall are expected to be rare – expected to occur in less than 1% of people (less than 1 out of 100 people). Gardner et al, 2000,(77) reviewed controlled clinical trials of exercise interventions for older adults at-risk for falling. No cardiac events or falls were reported in the 12 clinical trials reviewed. The at-risk older adults in the studies reviewed have slightly poorer physical performance than the older persons we expect to recruit for our study. In our recent MOBILE study, a 1 year cohort study of 120 older adults participating in 3 clinic visits over a 1 year period there were no cardiac events or falls reported. In all conditions of testing in which the participant is standing and/or walking (eg conditions with a potential risk for falling), the participant will be directly supervised by the physical therapist. The physical therapist is present for all testing sessions. We expect this level of supervision reduces the risk of falling to an even greater degree.
2. Less severe risks of participation such as such as muscle soreness, fatigue, or minor sprains or strains with each assessment, are expected to be infrequent – expected to occur in 1-10% of people (1-10 out of 100 people). Gardner et al, 2000,(77) reviewed controlled clinical trials of exercise interventions for older adults at-risk for falling, finding reports of such side effects of the intervention reported in only 4 of the 12 studies reviewed. The side effects were not a reason for dropout from the study and were described as soreness or musculoskeletal symptoms, but no injuries.
3. There is a rare risk that confidentiality could be breached. All of the research records will be kept in a locked file cabinet and/or password protected files. All of the investigators and staff that assist with the management of the files are trained in the privacy and confidentiality regulations that govern research.

Minimizing Risk during Assessments and Interventions.

All assessments approved in this study are considered to be a part of everyday clinical practice. We have minimized risks we believe by applying usual safeguards for the physical therapy assessment of gait. Assessment side effects, such as muscle soreness, fatigue, or minor sprains or strains with each assessment, will be recorded by the physical therapist and monitored by Dr. Brach, the Principal Investigators in consultation with the physician investigator, Dr. Nadkarni. Based upon the existing literature and our own clinical experience, we anticipate the frequency of these side effects to be extremely low.(77)

We will maximize the safety of our subjects with the following procedures.

1. Individuals with absolute contraindications to testing will be excluded based on the inclusion/exclusion criteria.
2. Testing will be carefully monitored by a licensed physical therapist and will be adjusted according to the American College of Sports Medicine Guidelines for Exercise Testing and Prescription.(35)
3. In all conditions of testing in which the participant is standing and/or walking (eg conditions with a potential risk for falling), the participant will be directly supervised by the physical therapist.
4. Subjects will be required to get medical clearance from their primary care physician prior to initiating the exercise intervention.

Confidentiality.

Participant's confidentiality will be protected in the data collection process. All personnel involved with the research have read and signed a Confidentiality statement, and approval is being obtained from the University of Pittsburgh Biomedical Institutional Review Board. Consent forms and data collection forms that identify the participant by name will be stored in a locked cabinet. All computers are password protected. If the data are used in scholarly presentations or journal articles, the investigators will protect the anonymity of individual participants and will report only aggregate data (eg group means) where appropriate. The Principal Investigator will review data confidentiality processes monthly or as indicated with the project staff (Study coordinator and Physical Therapists). The Investigators are all certified in Research Practice Fundamentals, Human Subjects Research Module.

Management of Adverse Experiences

All assessments and exercise interventions will be delivered in the Physical Therapy Clinical and Translational Research Center (PT-CTRC). The emergency procedures are posted within the PT-CTRC (see attached) along with an AED. Emergency procedures are reviewed with the staff every 6 months. All PT-CTRC staff are certified in the American Heart Association BLS for Healthcare Providers (CPR and AED) program. Blood pressure and heart rate will be routinely monitored before and after the exercise interventions. Any symptoms that occur during activities performed by the participant as instructed by the study intervention protocol will be monitored and acted upon. Cardiac

symptoms will include the following: chest pain, shortness of breath, dizziness, syncope, lightheadedness, arm pain or numbness, acute onset fatigue/exhaustion, palpitation or fluttering in the chest, sweating and feeling of heart racing, acute onset severe headache, weakness or numbness in on one side, part or whole body, speech difficulties and fall to a lower level or floor. Cardiac signs will include the following: extremes of blood pressure or heart rate abnormality, wheezing, leg cramps or claudication, confusion or an acute change in participants' mental status, unresponsiveness or sluggish responses, fever, change in color of skin. Some of these activities may occur on the physical premises of the study site. If cardiac symptoms or signs arise during the exercise component of the intervention or in the physical premises, the staff will immediately terminate the exercise ensuring that the participant is seated or lying supine. Research staff will closely monitor the participant and if a medical emergency should occur, staff will immediately follow BLS procedures and call 911. They will describe the incident to the 911 operator and provide their location (Bridgeside Point I, Suite 470,100 Technology Drive, Pittsburgh PA 15219). Once the emergency situation is under control, the staff will contact the study coordinator and PI to notify them of the situation. If the symptoms are not serious enough to warrant CPR or to call 911 they will follow the procedures outlined in the manual of operations.

Participant Education about Potential Risks
Potential risks associated with study-related activities and interventions will be explained to each participant by trained study personnel during the informed consent process. Each participant will be instructed to report the occurrence of an AE to appropriate study staff at scheduled data collection times, to PTs administering the intervention, or spontaneously at any other time. Participants also will be encouraged to report concerns about the safety of participating in the study to any research staff.

8. Adverse Events and Serious Adverse Events

8.1 Overview
Expected adverse events (AEs) will be captured through interviews at 12, 24 and 36 weeks, based on the Health Status Update questionnaire. Reportable events are those events that have potential implications for participant safety and require individual reporting. Reportable AEs are defined as), AEs that have potential implications for participant safety, unexpected AEs, and injury that occurs while a participant is under the supervision of study related personnel. These RAEs will require individual event reporting as described in section 8.4. The timely and complete account of RAEs will be a critical requirement for the protection of human subjects in this trial.

The DSMB will review and approve study-defined expected AEs and will be involved in the regular monitoring of the RAE reporting system. Reporting of expected AEs to the DSMB, the NIA, and the IRB will be through the database. RAEs will be monitored and tracked separately and reviewed by the DSMB and the NIA as outlined in section 8.4.

8.2 Classifying Adverse Events
An AE is any unfavorable or unintended medical occurrence in a human study participant that has taken place during the course of a research project, including any

abnormal sign, symptom, or disease, whether or not related to participation in the research.

For the purposes of this study, any event that meets the criteria for an SAE, is unexpected, or results in injury to the participant while he/she is under the supervision of study related personnel will be classified as an RAE. Adequate review, assessment, and monitoring of RAEs require they be classified as to severity, expectedness, and potential relatedness to the study intervention.

8.2.1 Severity

The following guidelines will be used to determine level of severity:

Mild: Awareness of signs and symptoms, but easily tolerated and causing no loss of time from normal activities. No specific medical attention is required.

Moderate: Discomfort enough to cause a low level of inconvenience or concern to the participant and may interfere with daily activities. Symptoms may require minimal, local or noninvasive medical intervention.

Severe: Events interrupt the participant's normal daily activities and are usually incapacitating. Significant symptoms may require hospitalization or invasive medical intervention.

Life-threatening/Disabling: Events that may involve acute, life-threatening metabolic or cardiovascular complications (such as circulatory failure, hemorrhage, sepsis) or life – threatening physiological consequences. Intensive care or emergent invasive procedure is required.

Death: Causing death.

Severity is not synonymous with seriousness. A severe headache is not necessarily an RAE. However, mild chest pain may results in a day's hospitalization and thus would be classified as a RAE.

8.2.2 Expectedness

AEs will be assigned as to whether they were expected or unexpected based on current knowledge. Categories are defined as follows:

<u>Expected:</u> An AE that is anticipated on the basis of prior experience with the intervention under investigation; an event that can be attributed to the underlying condition of the participant being studied; or an event that can be attributed to the patient population being studied (see section--- Expected AEs). Expected AEs are captured in a standardized way by study personnel.

<u>Unexpected:</u> An AE that was not anticipated on the basis of prior experience with the underlying intervention under investigation; an event that can be attributed to the

underlying condition of the participant being studied; or to the patient population being studied or an expected event whose frequency or severity exceeds what is anticipated. Unexpected events are reportable.

8.2.3 Relatedness

The PI in consultation with the Co-Investigators and an independent safety monitor will determine the degree to which RAEs are related to study procedures using the criteria below.

Definitely related: The adverse event is clearly related to the investigational procedure – i.e., an event that follows a reasonable temporal sequence from administration of the study intervention, follows a known or expected pattern of response to the study intervention, that is confirmed by improvement on stopping and reappearance of the event on repeated exposure, and that could not be reasonably explained by the known characteristics of the participant's clinical state.

Possibly related: An adverse event that follows a reasonable temporal sequence from administration of the study intervention of that follows a known or expected pattern of response to the study intervention, but that could readily have been produced by a number of other factors.

Unrelated: The adverse event is clearly not related to the investigational procedure (i.e. another cause of the event is most plausible; and/or a clinically plausible temporal sequence is inconsistent with the onset of the event and the study intervention and/or a causal relationship is considered biologically implausible).

8.3 Expected AEs

Expected adverse events (AEs) will be captured through interviews at 12, 24 and 36 weeks, based on the Health Status Update questionnaire. The following are expected adverse events that have been listed in the informed consent form:

- Muscle soreness
- Fatigue
- Chest pain
- Breathing problems
- Cardiac event
- Fall (with or without injury)

8.4 Reportable AEs (RAEs)

Reportable AEs are events that have potential implications for participant safety and that require individual reporting. RAEs will be defined as events that fall into at least one of the following categories:

1. Serious adverse event (SAEs) - SAEs will be defined as any adverse event that results in death, is life threatening, or places the participant at immediate risk of death from the event as it occurred, requires or prolongs hospitalization, causes persistent or significant disability or incapacity, results in congenital abnormalities or birth defects, or is another condition which investigators judge to represent

significant hazards. Only SAEs that are possibly related to the study will be reported.

2. Unexpected AEs - An unexpected AE is defined as medical events that occur during study participation, but do not commonly occur in the study population and which are not listed in the informed consent document or study protocol.
3. AEs related or possibly related to the research intervention – defined as any AE which in the opinion of the principal investigator, the incident, experience or outcome more likely than not was caused by the procedures involved in the research.

Events that cannot be clearly defined as “reportable” will be discussed with the study physician and the PI to determine if they should be reported. All reportable events will be captured on an Adverse Event form which will be filed in the participant binder and then reported using the following guidelines.

8.5 Reporting of Events

The study PI has primary responsibility for the safety of participants as it relates to the study protocol. The study coordinator will be responsible for reviewing adverse events and assuring accurate and timely reporting of the adverse events. The co-investigators (including study physician) will review, evaluate and classify adverse events and provide follow-up for events until they are resolved. The DSMB will be responsible for monitoring study data for evidence of adverse effects attributable to participation in the study. The PI will be responsible for reporting study-defined AEs and SAEs to the University of Pittsburgh institutional review board (IRB) according to their timeline and format. The reporting sequence of RAEs will be as follows: trial coordinator, principal investigator, Dr. Nadkarni (Study MD), IRB, Dr. Studenski (DSMB) and Dr Wagster (NIA). Dr. Studenski and Dr. Wagster will determine which RAEs will be shared with the entire DSMB.
8.5.1 Serious Adverse Events. The occurrence of SAEs will be obtained on an Adverse Event form during the 12, 24 and 36 week testing sessions and by any study staff member who learns of a serious event. All study staff will be trained to recognize when there is a SAE and to follow appropriate reporting procedures as they become aware of a reportable event.

SAEs possibly related to the study will be reported to the University of Pittsburgh IRB, the DSMB, and NIA within 24 hours of learning of the event. The expedited report of the SAE will be submitted by telephone, fax, or email. Recognizing that the information available during this 24 hour period may not be sufficient to permit accurate completion of the required adverse event reporting forms, a detailed written SAE report will be completed as soon as the information is available. Follow up information can be requested by the DSMB or the NIA or its representative.

8.5.2 Unexpected Adverse Event. An unexpected AE may be witnessed by a member of the research team or a staff member may be told about the occurrence or an

unexpected event that may meet the criteria for reporting. Unexpected AEs that have a potential relationship to study procedures and activities will be reported to the University of Pittsburgh IRB and the DSMB within 24 hours of learning of the event.

8.5.3 Adverse Events Related or Possibly Related to the Research Intervention. An AE which in the opinion of the principal investigator, the incident, experience or outcome more likely than not was caused by the procedures involved in the research will be reported to the IRB and the DSMB within 10 working days of the investigator learning of the event. If the AE is serious or unexpected it will be reported as described above.

8.6 Follow-up for Adverse Events

All RAEs will be forwarded to Dr. Studenski (Independent Safety Monitor – ISM) for adjudication and follow-up. The ISM may contact the study coordinator to request additional information from the participant, significant other, or their health care provider and may seek medical records from a physician or care setting if needed to make a determination about relatedness to the study. If the event is potentially related to the study, the ISM will contact the PI who will consider whether the event was listed in the protocol and consent form and whether modifications to the protocol and consent form should be considered. To ensure the appropriate classification of events, the clinical site physician and/or PI may also be called to review the information.
The ISM will be responsible for providing follow-up for ongoing reportable events as follows:

1. The ISM will follow up on RAEs until a final status has been determined for the event. Some RAEs may have a status that is still present at the conclusion of the study. The categories of RAE status are: not recovered/not resolved, recovered/resolved, recovered/resolved with sequelae, recovering/resolving, fatal, unknown.

2. Once the event is no longer ongoing and a final status for the event has been determined, the ISM will record the final status, enter the closed date, and sign and date the report.

8.6.1 Action Taken

The PI, in consultation with the study physician and the ISM, will decide whether or not an RAE requires that the participant be removed from the study intervention. The DSMB will be notified of the recommended course of action. Actions taken in response to the RAE will fall into one of four categories:

- No action taken
- Study procedure interrupted
- Study procedure discontinued
- Study procedure modified

8.7 Responsibilities

The PI has primary responsibility for the safety of participants as it relates to the study protocol. The study coordinator will be responsible for reviewing RAEs and assuring accurate and timely reporting of the RAEs. The study physician and the ISM will review, evaluate, and classify RAEs and provide follow-up for events until they are resolved. The DSMB will be responsible for reviewing and monitoring data for evidence of harm attributable to participation in the study.

8.7.1 Study Physician
The study physician will be available by telephone for consultation with the study personnel during all time periods when participants are engaged in the assessments or interventions. In addition, the study physician will be responsible for reviewing RAEs requiring immediate notification of the IRB and for being "on call" for study related emergencies. We will also obtain the name and contact information for the participant's primary care physician. In rare cases when the study physician is unavailable, the participant's primary physician will be contacted.

8.7.2 Independent Safety Monitor (ISM)
Dr. Studenski, the DSMB safety officer will serve as the ISM. The ISM will be responsible for:

- Reviewing reports of RAEs
- Confirming or refuting classification of the event as a RAE
- Requesting additional information as needed in order to make a determination
- Providing the PI with follow-up reports for ongoing RAEs as new information becomes available
- Notifying the PI of final status for the event once it has been determined

8.8 Reporting Expected AEs and RAEs to the DSMB
The DSMB will review tabulated data on non-serious and expected AEs on a semi-annual basis and monitor for adverse event rates out of proportion to those expected. The PI will forward the individual Reportable Adverse Event Records, including a narrative for each event, as well as a table showing all RAEs to the DSMB and NIA semi-annually. The DSMB will review all RAEs that are temporally related to the interventions in aggregate form at its scheduled meetings.

All RAEs that relate to hazards of the study interventions or are cause for urgent concern will be reported to the DSMB chairperson, the NIA and the University of Pittsburgh IRB immediately after recognition of their importance. If the DSMB chairperson concludes that an RAE is of universal and immediate concern, the DSMB chairperson may recommend convening the DSMB to review participant safety based on any individual report or accumulating evidence, including evidence according to treatment assignments.

9. <u>INTERVENTION DISCONTINUATION</u>

Certain events may result in a temporary interruption or early discontinuation of the trial assessments and interventions or components of these assessments and interventions.

Please refer to the appropriate MOP chapter(s) for specific instructions on stopping criteria during screening, assessment, interventions, and follow-up assessments.

After such events occur, a participant may resume the trial intervention when the study physician and the primary care provider agree that it is appropriate. For mild problems that require temporary cessation of the intervention, the PI in consultation with the study physician and the participant, may agree to reintroduce the participant to the study intervention.

At any time, the DSMB may recommend discontinuation of any component of the intervention or intervention group of the study for any of the following reasons:

1. Compelling evidence from this or any other study of an adverse effect of the study intervention(s) that is sufficient to override the potential benefit of the interventions to the target population
2. Compelling evidence from this or any other study of a significant beneficial effect of the study intervention(s), such that it is continued denial to other study group(s) would be unethical
3. A very low probability of addressing the study goals within a feasible timeframe.

The participants' participation in this research study is completely voluntary. The participant may withdraw, at any time, their consent for participation in this research study. Any identifiable research information recorded for, or resulting from, their participation in this research study prior to the date that they formally withdrew their consent may continue to be used and disclosed by the investigators. To formally withdraw consent for participation in this research study the participant should provide a written and dated notice of this decision to the principal investigator of this research study. If the participant withdraws from the intervention, study staff will ask permission to continue to follow the participant for follow-up assessment. If participation is discontinued for medical reasons and the participant is unable to complete the performance-based testing, all attempts will be made to obtain the self-reported outcomes.

10 DATA ANALYSES

All statistical analyses will be performed using SAS® version 9.3 (SAS Institute, Inc., Cary, North Carolina) based on the **intention-to-treat** philosophy. First, we will evaluate the distributional characteristics of the data set, the prevalence of missing values and general data quality using appropriate descriptive statistics (e.g. mean, median, standard deviation, range, frequencies, percentages) and graphical summarizations (histograms, boxplots, probability plots, scatterplots, lineplots) for all variables for each walk speed stratum for each intervention group for each available time point, as well as change scores from baseline for outcome variables. Second, the baseline pre-intervention values of these variables will be compared between the two intervention arms using independent samples *t*- or Wilcoxon rank sum tests, as appropriate, for continuous variables and a chi-square or Fisher's exact tests, as appropriate, for categorical variables. Although no significant differences are expected

due to randomized treatment assignment and the relatively large sample size, any variables found to be significantly different will be noted and accounted for, by controlling for them as additional covariates in supplements to main analyses. Third, main analyses to address the specific aims and hypotheses will be performed as outlined below. If an analysis of residuals reveals violations of standard assumptions of linear models, we will use a Box-Cox transformation(78) to the response variable before fitting the models. Multiple imputation will be used to account for any missing data in the main analysis.(79, 80) Finally, exploratory follow-up analyses will be performed as outlined below.

10.1 Main Analysis.

First, we will fit a series of linear mixed models using the SAS® MIXED procedure with change from baseline in each of the continuous outcome measures as the dependent variable; intervention arm (standard/standard plus), follow-up time point (12-/24-/36-week) and their interaction as fixed effects of interest; baseline value of outcome as a fixed-effect covariate; and a subject random effect to account for multiple follow-up assessments from the same patients over time and resulting stochastic non-independence of observations. We will appropriately construct means contrasts to compare the two intervention gains at 12 weeks (immediate effect), 24 weeks (delayed effect) and 36 weeks (sustained effect). Test of significance at $\alpha=0.05$ for gait speed outcome at 12 weeks will be considered the formal test of the primary hypothesis 1.1. Second, we will repeat the above mixed models analysis entirely, but with stratification by baseline gait speed (<1.0/1.0+ m/s). Tests of significance for gait speed outcome at 12 weeks in each of the two strata will be considered formal tests of the primary hypothesis 1.2. Statistical significance of at 24- and 36-week comparisons or other outcomes will be considered as formal tests of the secondary hypotheses involving activity and participation as well as delayed and sustained effects. Third, we will repeat the analyses after controlling for covariates identified a priori or found to be different between intervention arms by including them as additional fixed effects in supplements to main analyses.

10.2 Exploratory Analyses.

To address our subgroup analysis exploratory aim, we will repeat the main analyses after stratification by various other baseline participant characteristics (besides baseline gait speed) to identify, if any, subgroups which are most/least likely to benefit from interventions. A priori, we anticipate that stratification by baseline walking confidence and cognitive function may show differential gains due to the two interventions. We are aware that any definitive subgroup analyses should be supported by strong evidence in the form of a significant interaction effect.(81) Tests of interaction generally have low statistical power, and it is not feasible in the proposed Phase II efficacy trial to recruit the large number of participants required for definitive subgroup analyses beyond the already included pre-planned subgroups based on baseline walking speed. Thus, we will interpret our subgroup analyses with caution and an exploratory rather than a confirmatory philosophy, whose results are to be confirmed in a larger subsequent trial.

10.3 Compliance and Dropout Analyses.

We will estimate compliance by the number of sessions attended and estimate the proportion of subjects in each group with various levels of compliance. We also will calculate the proportion of subjects missing each session to describe the pattern of compliance. Dropout rates will be calculated as proportions of subjects randomized, and as a cumulative probability of remaining in the study, using survival analysis techniques such as the Kaplan-Meier product-limit estimator.(82) Unlike proportions, the latter statistics, which can be estimated at various times following randomization, take into account when dropouts occur. As with the compliance measure, these statistics will be calculated separately for each treatment group. The information contained in these descriptive analyses may help improve our retention strategies.

10.4 Interim Analysis

We do not plan to perform any interim analyses for efficacy. We will follow the recommendations of the external Data and Safety Monitoring Board (DSMB) to establish the specific rules for interim analyses for safety, either before commencement or very early stages of the trial. Specifically, we a priori plan to use the proportion of completed person-time of follow-up as the information statistic; test-statistic for comparison of proportions of (serious) adverse events to define the Brownian motion process; and an alpha-spending function discussed in Lan and DeMets (1983) to define a safety boundary. We will defer to the recommendation of DSMB regarding specific time points for interim safety analyses (expecting them to be approximately annually or otherwise coincide with DSMB meetings); whether the functional form of the alpha-spending function should be Pocock, Obrien-Fleming or another function; and any other aspect of interim safety monitoring.

10.5 Sample Size Adequacy

Sample size is estimated based on preliminary data from our pilot studies; our ability to detect statistical significance in two-tailed tests conducted at the α=0.05 level unless otherwise noted; and conservative expected dropout rates of 10, 15 and 20% during the 12-, 24- and 36-week follow-up. Thus, we conservatively anticipate 55, 52 and 49 participants to complete the 12-, 24- and 36-week assessments, respectively. We used standard sample size and power computation methods available in the literature and commercial software (PASS®; Number Cruncher Statistical Systems, Inc., Kaysville, Utah).

10.5.1 Primary Outcome

Our primary outcome on which the sample size justification is based is gait speed. Our preliminary data suggest a conservative estimate of between-subject standard deviation in baseline gait speed is 0.17 m/s and baseline to follow-up change in gait speed is 0.15 m/s. Moreover, a meaningful change in gait speed is approximately 0.10 m/s.(11) With the proposed number of 124 in each intervention, we will have >99% power to detect statistical significance of a between-intervention difference of such magnitude in 12-

week change (primary hypothesis 1.1). We anticipate that approximately half of the participants will belong to the slower baseline gait speed stratum. Thus, with the expected number of 62 participants within each arm within each stratum, we will be able to detect statistical significance of a between-intervention difference in 12-week change within each stratum (slower/faster baseline gait speed) with 96% statistical power (hypothesis 1.2). For 24- and 36-week gait speed change, we will have 92% and 90% statistical power, respectively, within each stratum (delayed effect and sustained effect hypotheses). If the anticipated split between slower and faster walker strata turns out to be no worse than 55%-45%, we will still have ≥90% power for both strata for hypothesis 1.2; and if it is no worse than 67%-33%, we will still have ≥80%.

10.5.2 Secondary and Tertiary Outcomes

Regarding secondary and tertiary outcomes, our preliminary data suggest conservative estimates of between-subject standard deviations in baseline measures of 0.9 s (Figure 8 time), 8.8 points (LLFDI function), 12.1 points (LLFDI disability limitation) and 53.1 m (6-minute walk test). Baseline to follow-up change standard deviations, respectively, were 1.08 s, 5.5 points, 11.8 points and 42.8 m. With the proposed sample size of 124 (and 98 anticipated completers at 36 weeks) per intervention arm, we will be able to detect statistical significance of between-intervention differences as small as 0.43 s, 2.2 points, 4.7 points and 17.2 m, respectively, with 80% power in two-tailed tests. These differences for the sustained effect hypotheses correspond to small-moderate Cohen's effect sizes of 0.25-0.48 for all secondary outcomes.(83) Statistical power for the immediate and delayed effect hypotheses should be even greater due to greater numbers of participants in study at the earlier 12- and 24-week time points.

In summary, we have proposed an adequate sample size for successfully addressing the aims of the project, with a higher degree of statistical sensitivity reflected by ≥90% power for detecting meaningful differences in the primary outcome gait speed; and adequate statistical sensitivity reflected by ≥80% power for detecting small to moderate effects in secondary and tertiary outcomes.

10.6 Treatment Assignment Procedures - Randomization

We will use the high quality pseudo-random deviate generator available in SAS® (SAS Institute, Inc., Cary, North Carolina) to randomize participants to standard or standard-plus interventions in a 1:1 ratio, stratified by whether a slower (<1.0 m/s) or faster (≥1.0 m/s) walker at baseline. Within each baseline gait speed stratum, we will do a blocked randomization to force continued approximate balance between the numbers of subjects in each arm during recruitment. The block size will be randomly selected to be 4 or 6 to prevent personnel from predicting treatment arm. For example, if the first two participants are randomized to active interventions, the assignment of the next participant cannot with certainty be predicted to be control. The study statistician will create randomization schedules for the two strata which contains a randomization sequence number (different from a participant's study identification number) and assigned arm. Randomization sequence numbers will be readily distinguishable between the strata (ie. starting from 4001 for slower and 8001 for faster baseline walkers). Then he will pass the randomization schedules to data management personnel so that they can be incorporated into the data management system itself. At

the time of randomization, research personnel will indicate the eligible participant to be randomized in the data management system. The system will first determine the stratum of the participant being randomized, and then use the next available randomization sequence number for that particular stratum to determine the intervention assignment based on the schedule constructed by the study statistician, record the intervention assignment in a restricted location in the database inaccessible by the blinded personnel, and retire the used randomization sequence number and randomized participant number from the randomization process. Personnel assessing follow-up outcomes will be blind to intervention assignment.

11 DATA COLLECTION AND QUALITY ASSURANCE

11.1 Data Collection Forms

Data collection will consist of a combination of paper forms and direct data entry. Data collected on paper forms will be entered into the electronic database by the research staff. A complete set of paper forms will be available at all times to be used if the database is temporarily unavailable. Please see the Manual of Procedures for a copy of all data entry forms.

Screening and baseline data collection, which will occur prior to randomization, will be conducted by research staff trained in the outcomes and may include the study coordinator if necessary. Outcome assessments at 12, 24 and 36 weeks will only be conducted by research staff trained in the outcomes and who are blinded to the intervention group assignment.

Participants' confidentiality will be protected in the data collection process. All study personnel are certified in Research Practice Fundamentals, Human Subjects Research Module. Consent forms and paper data collection forms will be stored in locked file cabinets. All computers are password protected. The database will be web-based and accessible with a username and password. Only authorized team members will have access to personal information needed for tracking and informed consent.

11.2 Data Management

The PI will oversee all aspects of data management. The Data Center (DC) will create an electronic System for Data Management (eSYSDM), based on detailed study protocols and requirements that includes an electronic case report form and a tracking system. The eSYSDM is developed using .NET 2.0 to create the interface and SQL for the database. The DC will work closely with investigative team and other study personnel to ensure that protocols are being followed, data integrity and confidentiality are maintained, and that the data contains a minimum amount of missing data. All study files residing in designated network folders will be backed-up daily and archived weekly. The weekly archived files are maintained for 1 year until the data are erased. All study subjects will be assigned unique study identifiers that will appear on all data collection instruments, tapes, documents, and files used in the statistical analysis and manuscript

preparation. Only authorized team members will have access to personal information needed for tracking and informed consent. Other data quality assurance measures will include detailed documentation of computer operations and data editing procedures and regular meetings with project staff to review any changes in procedure. The DC also has specific data quality measures that will be implemented. These include data verification, built in data validation mechanisms such as logic and out of range data checks, and repeated evaluation of the data collection and entry process.

11.3 Quality Assurance

Ongoing QA monitoring will be performed by the data center, study statistician (Dr. Perera) and the study coordinator. The DC or Dr. Perera will perform exploratory statistical investigations of aggregate data to identify unusual patterns and distributions. Monthly reports will be generated showing the frequency of missing data, delinquent forms, and other study performance parameters. These reports will be reviewed by the study coordinator who will promptly initiate action to remedy any problems, and will perform follow-up evaluations of actions taken, if necessary.

A quality assurance meeting will be held with all study staff prior to the enrollment of the first subject. Additional QA meetings will be held annually during the enrollment and follow-up period. If problems are identified, the PI will develop a corrective action plan for the staff with clearly defined tasks and timelines. The PI will track the implementation of the plan and assure that all tasks are completed within the defined timeframe.

At the QA meetings, study staff will discuss any difficulties they are having and will work collectively with the study investigators to resolve the problems. Additional meetings will be held if there are concerns about data quality or protocol adherence.

The purposes of the QA meetings will be to:

- Assure the rights and safety of participants
- Assure that informed consent has been obtained and documented in accordance with the protocol and NIH regulations
- Verify adherence to the protocol and exam staff knowledge via discussions
- Discuss screenings, informed consent process, assessments and interventions and to confirm that staff are following good clinical practice
- Ensure secure maintenance of required documents
- Review participant files for completeness
- Assure that information recorded on forms is complete and accurate
- Assure accurate reporting and documentation of all AEs
- Review data quality reports

Protocol deviations will be captured on the protocol deviation form and entered into the database. Protocol deviations will reviewed and discussed at monthly team staff meetings. The study coordinator, with input from the study investigators, will promptly

initiate action to remedy any problems, and will perform follow-up evaluations of actions taken, if necessary.

12 PARTICIPANT RIGHTS AND CONFIDENTIALITY

12.1 Institutional Review Board (IRB) Review

The study protocol, the informed consent document and any subsequent modifications will be reviewed and approved by the University of Pittsburgh IRB.

12.2 Informed Consent Forms

All potential participants will be adults (65 years of age or older) who are capable of providing direct consent for their participation in the study. Written informed consent will be obtained at the clinic screening visit prior to performing any of the clinic screening procedures. One of the study investigators will explain the study and the participant will be given a copy of the consent form to read. The consent form will describe the purpose of the study, the procedures to be followed, and the risks and benefits of participation. The investigator will answer any questions that the participant may have about the study. Finally, the participant will be asked to sign and date the consent form. The participant will be given a copy of the consent form for their records.

12.3 Participant Confidentiality

Participant privacy will be protected throughout the research process. Research assessments and interventions are conducted in the PT-CTRC located in the Bridgeside Point Building at the University of Pittsburgh. This space includes several small rooms that can be used to privately conduct questionnaires and simple physical examination measures. Many of the walking assessments are done in the open area of the center, which includes the oval track, the computerized walkway system and treadmills. Participants may be screened individually, which further protects their privacy. Participants are informed via the consent process that the treatment programs may be conducted in small groups. Although not completely private, this level of exposure to others during exercise is similar to what one might experience at a physical therapy appointment or during an exercise routine at a public gym. This research will not involve the use or disclosure of existing identifiable medical information, such as personal medical or hospital records.

Participants' confidentiality will be protected in the data collection process. Consent forms and data collection forms that identify the participant by name will be stored in a locked cabinet. All computers are password protected. If the data are used in scholarly presentations or journal articles, the investigators will protect the anonymity of individual participants and will report only aggregate data (eg group means) where appropriate. The Principal Investigator will review data confidentiality processes monthly or as indicated with the project staff (Study coordinator and Physical Therapists). The Investigators are all certified in Research Practice Fundamentals, Human Subjects Research Module.

12.4 Study Discontinuation

The study may be discontinued at any time by the IRB, the NIA, the OHRP, or other government agencies as part of their duties to ensure that research participants are protected.

13 PUBLICATION OF RESEARCH FINDINGS

Publications will be operationally defined as manuscripts for publications; abstracts for platform or poster presentation at scientific meetings and other professional meetings; slides for presentation at scientific and other meetings; doctoral dissertations; and master's theses.

The goal of the publication policy is to encourage and facilitate publication of study results. The purposes of this policy are to ensure the following:

- PRIMA publication will be of the highest scientific quality
- PRIMA will be described in a consistent manner across publications
- Measures are reported in consistent ways across publications
- Proper acknowledgements are included
- Appropriate authorship credit is determined prior to submission of manuscripts for publication consideration.

Publications from PRIMA will be overseen by the PI and Co-Investigators.

14 ANCILLARY STUDY POLICIES

An ancillary study will be defined as a study that (1) uses supplementary data that will be collected on participants who are recruited in PRIMA, over and above the data collection required by the PRIMA protocol, (2) collects biological specimens (e.g. blood) or performs diagnostic tests (e.g. bone density scans); and/or (3) collects data on subjects not enrolled in PRIMA but who may be compared to PRIMA subjects (e.g. participant who receive an alternative intervention). Ancillary studies will be distinct from databank studies, which use data previously collected on participants who are enrolled in PRIMA.

Ancillary studies will be reviewed and approved by the PI and CO-I's prior to initiation to ensure they do not conflict with the main study protocol. All approved ancillary studies will also be reviewed by the DSMB and NIA prior to initiation. If approved, the ancillary study PI will report to the DSMB on the same schedule as the main study. Review by the PI and Co-I's will also be required for presentation or publication of ancillary study results.

PRIMA investigators will be encouraged to consider ancillary studies and to involve other investigators, within and outside of PRIMA personnel. Participation in an ancillary

study will be subject to approval by the PI, Co-I's and DSMB. The following factors will be considered in determining approval of the proposed ancillary study:

1. Participant burden
   a. The proposed study must be acceptable to the participants (e.g. in terms of time, discomfort, privacy, etc).
   b. The proposed study must not reduce enrollment or hamper continued participation in the main study.
2. Study interference
   a. The proposed study must not interfere with the other parts of the main study.
   b. The proposed study must put little to no additional demands on the PRIMA resources.
3. The prosed study must be of the highest scientific merit.
4. The investigators must have adequate resources to effectively complete the ancillary study including:
   a. Sufficient budget
   b. Staff having the required expertise to meet the objectives of the project.

15 REFERENCES

1. Guralnik J, Ferrucci L, Pieper C, Leveille S, Markides K, Ostir G, et al. Lower extremity function and subsequent disability: consistency across studies, predictive models, and value of gait speed alone compared with the short physical performance battery. J Gerontol Med Sci. 2000;55A:M221-M31.
2. Cesari M, Kritchevsky S, Bauer D, Visser M, Rubin S, Harris T, et al. Prognostic value of usual gait speed in well-functioning older people--results from the Health, Aging and Body Composition Study. J Am Geriatr Soc. 2005;53:1675-80.
3. Studenski S, Perera S, Patel K, Rosano C, Faulkner K, Inzitari M, et al. Gait speed and survival in older adults. JAMA. 2011;305(1):50-8.
4. Guralnik J, Ferrucci L, Simonsick E, Salive M, Wallace R. Lower extremity function in persons over the age of 70 years as a predictor of subsequent disability. New Engl J Med. 1995;332:556-61.
5. Guralnik J, Simonsick E, Ferrucci L, Glynn R, Berkman L, Blazer D, et al. A short physical performance battery assessing lower extremity function: Association with self-reported disability and prediction of mortality and nursing home admission. J Gerontol. 1994;49:M85-M94.
6. Fried L, Bandeen-Roche K, Chaves P, Johnson B. Preclinical Mobility Disability Predicts Incident Mobility Disability in Older Women. Journal of Gerontology. 2000;55A(1):M43-M52.
7. Hoffman J, Ciol M, Huynh M, Chan L. Estimating transitions probabilities in mobility and total costs for Medicare beneficiaries. Arch Phys Med Rehabil. 2010;91:1849-55.
8. Pate R, Pratt M, Blair S, Haskell W, Macera C, Bouchard C, et al. Physical activity and public health: a recommendation from the Centers of Disease Control and Prevention and the American College of Sports Medicine. JAMA. 1995;273(5):402-7.
9. Nelson M, Rejeski W, Blair S, Duncan P, Judge J, King A, et al. Physical activity and public health in older adults: recommendation from the American College of Sports Medicine and the American Heart Association. Med Sci Sports. 2007;39(8):1435-45.

10. LIFE S, Investigators. Effects of a physical activity intervention on measures of physical performance: results of the Lifestyle Interventions and independence for elders pilot (LIFE-P) study. J Gerontol Med Sci. 2006;61A:1157-65.
11. Perera S, Mody S, Woodman R, Studenski S. Meaningful change and responsiveness in common physical performance measures in older adults. J Am Geriatr Soc. 2006;54:743-9.
12. Brach J, Studenski S, Perera S, VanSwearingen J, Newman A. Stance time and step width variability have unique contributing impairments in older persons. Gait Posture. 2008;27:431-9.
13. Ferrucci L, Baninelli S, Benvenuti E, DiIorio A, Macchi C, Harris T, et al. Subsystems contributing to the decline in ability to walk: Bridging the gap between epidemiology and geriatric practice in the InCHIANTI study. J Am Geriatr Soc. 2000;48(12):1618-25.
14. Haley S, Jette A, Coster W, Kooyoomijian J, Levenson S, Heeren T, et al. Late life function and disability instrument:II. Development and evaluation of the function component. J Gerontol. 2002;57A:M217-M22.
15. Milton J, Small S, Solodkin A. On the road to automatic: dynamic aspects in the development of expertise. J Clin Neurophys. 2004;21:134-43.
16. Brooks V. The Neural Basis of Motor Control. New York: Oxford University Press; 1986.
17. McArdle W, Katch F, Katch V. Exercise Physiology: Energy, Nutrition, and Human Perofrmance. Fifth ed. Baltimore, MD: Lippincott Williams & Williams; 2001.
18. VanSwearingen J, Perera S, Brach J, Wert D, Studenski S. Exercise to improve gait efficiency: impact on activity and participation in older adults with mobility limitations. Phys Ther. 2011;91:1740-51.
19. de V, PL, Samson M, van M, NL, Duursma S, Verhaar H. Functional-task exercise versus resistance strength exercise to improve daily function in older women: a randomized controlled trial. J Am Geriatr Soc. 2005;53:2-10.
20. Judge J. Balance training to maintain mobility and prevent disability. Am J Prev Med. 2003;25(3 Suppl 2):150-6.
21. Brown M, Holloszy J. Effects of a low intensity exercise program on selected physical performance characteristics of 60- to 71-year olds. Aging (Milano). 1991;3:129-39.
22. Fiatarone M, Marks M, Ryan E, Meredith N, Lipsitz C, Evans W. High intensity strength training in nonagenarians. Effects on skeletal muscle. JAMA. 1990;263:3029-34.
23. Judge J, Underwood M, Gennosa T. Exercise to improve gait velocity in older persons. Archives of Physical Medicine and Rehabilitation. 1993;74(4):400-6.
24. Fiatarone M, O'Neill E, Ryan N, Clements K, Solares G, Nelson M, et al. Exercise training and nutritional supplementation for physical frailty in very elderly people. New Engl J Med. 1994;330:1769-75.
25. Topp R, Mikesky A, Wigglesworth J, Holt W, Edwards J. The effect of a 12-week dynamic resistance strength training program on gait velocity and balance of older adults. Gerontologist. 1993;33(4):501-6.
26. Menz H, Lord S, Fitzpatrick R. Age-related differences in walking stability. Age Aging. 2003;32:137-42.
27. Morgan M, Phillips J, Bradshaw J, Mattingley J, Iansek R, Bradshaw J. Age-related motor slowness: simply strategic? Journal of Gerontology. 1994;49(3):M133-M9.
28. Welford A. Motor Skills and Aging. In: Mortimer J, Pirozzolo F, Maletta G, eds. The Aging motor System. New York: Praeger Publishers; 1982:152-87.
29. Welford A. Between bodily performance and slowing with age. Exp Aging Res. 1984(10):73-88.
30. VanSwearingen J, Perera S, Brach J, Cham R, Rosano C, Studenski S. A randomized trial of two forms of therapeutic activity to improve walking: effect on the energy cost of walking. J Gerontol A Biol Sci Med Sc. 2009;64A:1190-8.

31. Brach J, VanSwearingen J, Perera S, Wert D, Studenski S. Motor learning versus standard walking exercise in older adults with subclinical gait dysfunction: A randomized clinical trial. J Am Geriatr Soc. 2013;61:1879-86.
32. VanSwearingen J, Perera S, Brach J, Cham R, Rosano C, Studenski S. Exercise to reduce the energy cost of walking: a randomized trial. J Gerontol Med Sci. 2009;64(1):1190-8.
33. Brach J, Lowry K, Perera S, Wert D, Hornyak V, Studenski S, et al. Improving motor control in walking: a randomized clinical trial in older adults with subclinical walking difficulty. . Arch Phys Med Rehabil. 2014;in press.
34. Kramer M, McWilliams J, Chen H, Siminerio L. A community-based diabetes prevention program: evaluation of the Group Lifestyle Balance Program devilvered by diabetes educators. The Diabetes Educator. 2011;37(5):659-68.
35. ACSM's Guidelines for Exercise Testing and Prescription. 5th ed. Baltimore, MD: Williams & Wilkins; 1995.
36. Nelson W. Physical principles for economies of skilled movements. Biol Cybernetics. 1983;46:135-47.
37. Daly J, Ruff R. Construction of efficacious gait and upper limb functional interventions based on brain plasticity evidence and model-based measures for stroke patients. The Scientific World Journal. 2007;7:2031-45.
38. Lay B, Sparrow W, Hughes K, O'Dwyer N. Practice effects on coordination and control, metabolic energy expenditure, and muscle activation. Human Movement Science. 2002;21:807-30.
39. Newman M, Dawes H, van d, Berg, M, Wade D, Burridge J, Izadi H. Can aerobic treadmill training reduce the effort of walking and fatigue in people with multiple sclerosis: a pilot study. Multiple Sclerosis. 2007;13:113-9.
40. Gentile A. Skill acquisition: action, movement, and neuromotor processes. In: JH C, RB S, J G, AM G, JM H, eds. Movement Sciences. 1 ed. Rockville: Aspen Publishers; 1987:93-154.
41. Polcyn A, Lipsitz L, Kerrigan C, Collins J. Age-related changes in the initiation of gait: degradation of central mechanisms for momentum generation. Arch Phys Med Rehabil. 1998;79:1582-9.
42. Capaday C. The special nature of human walking and its neural control. Trends in Neurosciences. 2002;25(7):370-6.
43. Alexander R. Walking made simple. Science. 2005;308:58-9.
44. Schmidt R. Organizing and Scheduling Prcatice. In: RA S, ed. Motor Learning and Practice: From Principles to Practice. Champaign, IL: Human Kinetics Books; 1991:199-225.
45. Brach J, VanSwearingen J. Interventions to improve walking in older adults. Curr Transl Geriatr and Exp Gerontol Rep. 2013;2:230-8.
46. Brach J, Perera S, Studenski S, Newman A. Reliability and validity of measures of gait variability in community-dwelling older adults. Arch Phys Med Rehabil. 2008;89:2293-6.
47. Mangione K, Craik R, McCormick A, Blevins H, White M, Sullivan-Marx E, et al. Detectable changes in physical performance measures in elderly African Americans. Phys Ther. 2010;90(6):921-7.
48. Harada N, Chiu V, Damron-Rodriguez J, Fowler E, Siu A, Reuben D. Screening for balance and mobility impairment in elderly individuals living in residential care facilities. Physical Therapy. 1995;75(6):462-9.
49. Hardy S, Perera S, Roumani Y, Chandler J, Studenski S. Improvement in usual gait speed predicts better survival in older adults. J Am Geriatr Soc. 2007;55(11):1727-34.
50. Brach J, et a. Meaningful change in measures of gait variability in older adults. J Am Geriatr Soc. 2008;submitted.

51. Callahan D, Phillips E, Carabello R, Frontera W, Fielding RA. Assessment of lower extremity muscle power in functionally-limited elders. Aging Clin Exp Res. 2007;19(3):194-9.
52. Butland R, Pang J, Gross E, Woodcock A, Geddes D. Two-, six-, and 12-minute walking tests in reespiratory disease. BMJ. 1982;284:1607-8.
53. Harada N, Chiu V, Stewart A. Mobility-related function in older adults: assessment with a 6-minute walk test. Arch Phys Med Rehabil. 1999;80:837-41.
54. Guyatt G, Sullivan M, Thompson P. The 6-minute walk: a new measure of exercise capacity in patients with chronic heart failure. Can Med Assoc. 1985;132:919-23.
55. Solway S, Brooks D, Lacasse Y, Tomas S. A qualitative, systematic overview of the measurement properties of the functional walk tests used in the cardioresepiratory domain. Chest. 2001;119:256-70.
56. Jones C, Rikli R, Max J, Noffal G. The reliability and validity of a chair Sit-and-Reach test as a measure of hamstring flexibility in older adults. Research Quarterly for Exercise and Sport. 1998;69(4):338-43.
57. Menz H, Lord S, Fitzpatrick R. Acceleration patterns of the head and pelvis when walking on level and irregular surfaces. Gait Posture. 2003;18:35-46.
58. Brach J, McGurl D, Wert d, VanSwearingen J, Perera S, Cham R, et al. Validation of a measure of smoothness of walking. J Gerontol A Biol Sci Med Sc. 2011;66:136-41.
59. Gabell A, Nayak U. The effect of age and variability in gait. Journal of Gerontology. 1984;39(6):662-6.
60. Brach J, Studenski S, Perera S, VanSwearingen J, Newman A. Gait variability and the risk of incident mobility disability. J Gerontol Med Sci. 2007;62A:983-8.
61. Perera S, Brach J, Talkowski J, Wert d, Studenski S. Measuring stride time variability: estimating test-retest reliability and required walk length using bootstrapping. Program & Abstracts of the ISPGR 18th International Conference. 2007:55-6.
62. Brach J, Berlin J, VanSwearingen J, Newman A, Studenski S. Too much or too little step width variability is associated with a fall history in older persons who walk at or near normal gait speed. J Neuroengineering Rehabil. 2005;2(21).
63. Boyd R, Rodda J, Olesch C, et a. High- or low-technology measurements of energy expenditure in clinical gait analysis? Dev Med Child Neurol. 1999;41:676-82.
64. MacGregor J. The objective measurement of physical performance with long term ambulatory physiological surveillance equipment (LAPSE). In: FD S, EB R, L G, eds. Proceedings of the Third International Symposium on Ambulatory Monitoring. London: Academic Press; 1980:29-39.
65. Waters R, Lunsford B. Energy cost of paraplegic ambulation. J Bone Joint Surg. 1985;67:1245-50.
66. Bernardi M, Macaluso A, Sproviero E, Castellano V, Coratella D, Felici F, et al. Cost of Walking and Locomotor Impairment. Journal of Electromyography and Kinesiology. 1999;9(2):149-57.
67. Macko R, Katzel L, Yataco A, Tretter L. Low-velocity graded treadmill stress testing in hemiparetic stroke patients. Stroke. 1997;28(5):988-92.
68. World H, Organization. ICF: International Classification of Functioning, Disability and Health., 5/22/1 AD 1905. Geneva, Switzerland: World Health Organization; 2001.
69. Jette A, Haley S, Coster W, Kooyoomijian J, Levenson S, Heeren T, et al. Late life function and disability instrument: I. Development and evaluation of the disability component. J Gerontol. 2002;57A:M209-M16.
70. Welk G, Schaben J, Morrow J. Reliability of accelerometry-based activity monitors: a generalizability study. Med Sci Sports Exer. 2004;36(9):1637-45.

71. Bassett D, Ainsworth B, Swartz A, Strath S, O'Brien W, King G. Validity of four motion sensors in measuring moderate intensity physical activity. Med Sci Sports Exer. 2000;32:S471-S80.
72. Reitan R, Wolfson D. The Hasltead-Reitan Neuropsychological Test battery: Therapy and Clinical Interpretation. Tucson, AZ: Neuropsychological Press; 1985.
73. Yesavage J, Brink T, Rose T, Lum O, Huang V, Adey M. Development and validation of a geriatric depression screening scale: a preliminary report. J Psychiatr Res. 1982;17(1):37-49.
74. Buysse D, Reynolds C, 3rd, Monk T, Berman S, Kupfer D. The Pittsburgh Sleep Quality Index: a new instrument for psychiatric practice and research. Psychiatry Res. 1989;28:193-213.
75. Peel C, Sawyer B, Roth D, Brown C, Bodner E, Allman R. Assessing Mobility in Older ADults: The UAB Study of Aging Life-Space Assessment. Physical Therapy. 2005;85(10):1008-19.
76. Newell A, VanSwearingen J, Hile E, Brach J. The modified gait efficacy scale: establishing the psychometric properties in older adults. Phys Ther. 2012;92:318-28.
77. Gardner M, Robertson C, Campbell A. Exercise in preventing falls and fall related injuries in older people: a review of randomized controlled trials. Br J Sports Med. 2000;34:7-17.
78. Box G, Cox D. An analysis of transformations. Journal of the Royal Statistical Society-Series B. 1964;26:211-43.
79. Rubin D. Multiple Imputation for Nonresponse in Surveys: John Wiley and Sons; 1987.
80. Rubin D. Multiple imputation after 18+ years. Statistics in Medicine. 1991;14:1913-25.
81. Wang R, Lagakos S, Ware J, Hunter D, Drazen J. Statistics in medicine-reporting of subgroup analyses in clinical trials. N Engl J Med. 2007;357(21):2189-94.
82. Lawless J. Statistical Models and Methods for Lifetime Data. New York: Wiley; 2002.
83. Cohen J. Satistical Power Analysis for the Behavioral Sciences. New York: Academic Press; 1977.

Summary of Modifications from Original Protocol to Final Protocol

Version (date)	Section	Brief Summary of Modification
Version 1.1 (2/9/2016)	4.1	Removed inclusion criteria #5 – Not meeting physical activity recommendations defined as reporting less than 150 minutes of moderate intensity activity per week in the past month. This question will remain on phone screen so we can track information.
Version 1.2 (3/15/2016)	5.4	Updated strength training description—all subjects will completed hip extension and abduction strengthening as well as 1-2 other LE strengthening exercises at the PTs discretion
Version 1.2 (3/15/2016)	6.1	Changed timepoint for "medical clearance to participate" to clinic screen; removed CHAMPS questionnaire; added Life Space Assessment and Pitt Sleep Quality Index for baseline, 12 week, 24 week, and 36 week timepoints
Version 1.2 (3/15/2016)	6.2.3	Removed CHAMPS; added Life Space Assessment
Version 1.3 (6/6/2016)	5.6.2	Changed monthly phone calls to monthly check ins to be documented by study coordinator
Version 1.3 (6/6/2016)	5.7	Updated requirements for schedule intervention visits to include first session 8-14 days after baseline testing; added randomization timing to details
Version 1.4 (8/19/2016)	8.5-8.7	Updated information on who RAEs should be reported to and in which order. SAEs that are not related to the protocol will not be considered RAEs.

Original statistical analysis plan as published in Brach et al, 2020[1] and documented in clinicaltrials.gov.

Overview. All statistical analyses will be performed based on the intention-to-treat philosophy. First, the baseline pre-intervention values of participant characteristics and measurements will be compared between the two arms with and without consideration of gait speed stratum using analysis of variance, Kruskal-Wallis, chi-square and/or Fisher's exact tests, as appropriate, depending on type and distribution of variables. No significant differences are expected due to randomized intervention assignment and the large sample size, but any found to be significantly different will be noted and accounted for, by controlling for them as additional covariates in supplements to the main analyses. We will not alter the main analyses to preserve its a priori and predictable nature. Next, main analyses to address the specific aims and hypotheses will be performed. If an analysis of residuals reveals violations of standard assumptions of linear models, we will use a Box-Cox transformation[2] to the response variable before fitting the models. Multiple imputation will be used to account for any missing data in the main analysis.[3,4] Finally, many exploratory follow-up analyses will be performed as outlined below.

Main Analysis. We will fit a series of linear mixed models using the SAS® MIXED procedure with change from baseline in each of the continuous outcome measures as the dependent variable; intervention arm (standard/standard plus), follow-up time point (12-/24-/36-week) and their interaction as fixed effects; baseline value of outcome as an additional fixed effect covariate; and a participant random effect. We will appropriately construct means contrasts to compare the intervention gains at 12, 24 and 36 weeks. The tests of significance at $\alpha=0.05$ for the 12-week gait speed means contrast will be considered the formal test of the primary hypothesis. Next, we will repeat the above mixed model analysis entirely, but with

stratification by baseline gait speed (<1.0/1.0+ m/s). Statistical significance of 24- and 36-week means contrasts and other outcomes will be considered as tests of the secondary hypotheses involving process, activity and participation outcomes as well as persisting effects.

Exploratory Analyses. To examine subgroup-specific intervention effects, we will repeat the main analyses after stratification by various other baseline participant characteristics (besides baseline gait speed) to identify, if any, subgroups which are most/least likely to benefit from interventions. A priori, we anticipate that stratification by baseline walking confidence and cognitive function may show differential gains. We are aware that any definitive subgroup analyses should be supported by strong evidence in the form of a significant interaction effect.[5] Tests of interaction generally have low statistical power, and it is not a main goal in the present trial to recruit a large number of participants required for definitive subgroup analyses beyond the already included pre-planned subgroups based on baseline walking speed. Thus, we plan to interpret our subgroup analyses with caution and an exploratory rather than a confirmatory philosophy, whose results are to be confirmed in a larger subsequent trial.

Final Statistical analysis plan (current, submitted manuscript)

See published protocol for details.[1] We compared baseline characteristics between groups using independent samples t-, chi-square and Fisher's exact tests. For main results, we performed an intention-to-treat analysis with multiple imputation for missing data.[4] We fit linear mixed models with change from baseline in each continuous outcome measure as the dependent variable; intervention arm, follow-up time point and their interaction as fixed effects; baseline value of outcome as a fixed effect covariate; and a participant random effect. For outcomes based on accelerometers, wear time was an additional covariate. We constructed means contrasts to

compare the intervention gains at each of the follow-up time points. We repeated the analysis with gait speed stratum and stratum × intervention as additional fixed effects to obtain stratum-specific findings. SAS® version 9.4 software (SAS Institute, Inc., Cary, NC) was used.

Summary of Changes

There were three unplanned post-completion deviations, all relatively minor or necessary:

(1) In the subgroup analysis, instead of repeating the main analysis simply stratified by baseline slow/fast stratum, we had included it as an additional fixed effect with interaction terms. This was done so that we could obtain statistical significance of differential findings in the slow and fast strata.

(2) Actigraphy physical activity variables were analyzed separately with respect to multiple imputation from other outcomes due to they not being required prior to randomization, and the resulting large number without that data.

(3) Statistical models for actigraphy physical activity variables included wear time as an additional covariate, in keeping with the standard practice in the field.

References

1. Brach JS, VanSwearingen JM, Gil A, et al. Program to improve mobility in aging (PRIMA) study: Methods and rationale of a task-oriented motor learning exercise program. *Contemp Clin Trials.* 2020;89:105912.
2. Box G, Cox D. An analysis of transformations. *Journal of the Royal Statistical Society-Series B.* 1964;26:211-243.

3. Rubin C, Sizemore M, Loftis P, Loret d, Mola, N. A randomized, controlled trial outpatient geriatric evaluation and management in a large public hospital. *J Am Geriatr Soc.* 1993;41:1023-1028.
4. Rubin D. *Multiple Imputation for Nonresponse in Surveys.* John Wiley and Sons; 1987.
5. Wang R, Lagakos S, Ware J, Hunter D, Drazen J. Statistics in medicine-reporting of subgroup analyses in clinical trials. *N Engl J Med.* 2007;357(21):2189-2194.
