# Supplementary material for: Effect of Timing and Coordination Training on Mobility and Physical Activity Among Community-Dwelling Older Adults: A Randomized Clinical Trial
Source: JAMA Netw Open. 2022 May 23;5(5):e2212921. doi: 10.1001/jamanetworkopen.2022.12921 (PMC9127558; doi:10.1001/jamanetworkopen.2022.12921)
Supplement: Supplement 2. — eTable 1. Inclusion and Exclusion Criteria eTable 2. Physical Activity Subsample: Participant Characteristics and Measures at Baseline: Mean ± Standard Deviation or N (%) eTable 3. Intervention Effects on Secondary Outcomes (Representing Components of Intervention and Additional Mobility Measures) Stratified by Screening Gait Speed eTable 4. Intervention Effects on Tertiary Outcomes (Self-Report Activity and Participation) [file jamanetwopen-e2212921-s002.pdf]

Supplemental Online Content

Brach JS, Perera S, Shuman V, et al. Effect of timing and coordination training on mobility and physical activity among community-dwelling older adults: a randomized clinical trial. *JAMA Netw Open.* 2022;5(5):e2212921. doi:10.1001/jamanetworkopen.2022.12921

eTable 1. Inclusion and Exclusion Criteria

eTable 2. Physical Activity Subsample: Participant Characteristics and Measures at Baseline: Mean ± Standard Deviation or N (%)

eTable 3. Intervention Effects on Secondary Outcomes (Representing Components of Intervention and Additional Mobility Measures) Stratified by Screening Gait Speed

eTable 4. Intervention Effects on Tertiary Outcomes (Self-Report Activity and Participation)

This supplemental material has been provided by the authors to give readers additional information about their work.

© 2022 Brach JS et al. *JAMA Network Open.*

eTable 1. Inclusion and Exclusion Criteria

Inclusion Criteria (all criteria must be met)	Exclusion Criteria (one or more)
1. 65 years of age and older<br>2. Ambulatory without an assistive device or the assistance of another person<br>3. Usual 4-meter gait speed between 0.60 and 1.2 m/s<br>4. Physician clearance to participate in a moderate intensity exercise program	1. Inability to participate in testing or exercise intervention:<br>a. persistent lower extremity pain that is present on most days of the week and crepitus, tenderness or enlargement of joints of the lower extremity (arthritis)<br>b. back pain that is present on most days of the weeks and interferes with walking and activities of daily living or back pain that increases with walking (lumbar stenosis)<br>c. calf pain or cramping which worsens with walking and is relieved by rest (PAD)<br>d. refusal to walk on a treadmill<br>e. plans to move out of the area in the following year
	2. Safety concerns:<br>a. dyspnea at rest or during activities of daily living or use supplemental oxygen (CHF, COPD)<br>b. any acute illness or medical condition that is not stable according to the approving physician<br>c. resting systolic blood pressure ≥ 200 mm Hg or diastolic blood pressure ≥ 100 mm Hg or resting heart rate > 100 or < 40 beats per minute<br>d. diagnosis of any dementia or cognitive impairment defined as 3MS< 79<br>e. hospitalized in the past 6 months for acute illness or surgery, other than minor surgical procedures<br>f. severe visual impairment as indicated by difficulty navigating the clinic<br>g. history of stroke<br>h. fixed or fused lower extremity joints such as hip, knee or ankle

© 2022 Brach JS et al. *JAMA Network Open.*

	i. lower extremity strength <3/5 on manual muscle testing<br>j. lower extremity amputation<br>k. progressive movement disorder such as MS, ALS or Parkinson's disease

© 2022 Brach JS et al. *JAMA Network Open.*

eTable 2. Physical Activity subsample: Participant characteristics and measures at baseline: mean ± standard deviation or N (%)

Characteristic	Standard-Plus N=118	Standard N=122
Age	77.1±6.6	77.5±6.4
Female gender	82 (69.5)	77 (63.1)
Race		
Asian	1 (0.9)	0 (0.0)
Black	7 (5.9)	12 (9.8)
Pacific Islander	1 (0.9)	1 (0.8)
White	105 (89.0)	108 (88.5)
Other	1 (0.9)	0 (0.0)
N/A	3 (2.5)	1 (0.8)
Live alone	56 (47.5)	56 (45.9)
Marital status		
Married	57 (48.3)	54 (44.3)
Separated	0 (0.0)	2 (1.6)
Divorced	18 (15.3)	16 (13.1)
Widowed	26 (22.0)	34 (27.9)
Never Married	13 (11.0)	15 (12.3)
Other	4 (3.4)	1 (0.8)
Education		
Grade 9-12	20 (17.0)	18 (14.8)
College	45 (38.1)	41 (33.6)
Post-graduate	50 (42.4)	58 (47.5)
Other	3 (2.5)	5 (4.1)
Duke comorbidity index	2.9±1.3	2.9±1.3
Cardiac	12 (10.2)	11 (9.0)
Neurologic	8 (6.8)	7 (5.7)
Musculoskeletal	102 (86.4)	105 (86.1)
General	31 (26.3)	36 (29.5)
Visual/hearing	88 (74.6)	96 (78.7)
Diabetes	26 (22.0)	24 (19.7)
Cancer	40 (33.9)	45 (36.9)
Lung	32 (27.1)	30 (24.6)
Geriatric Depression Scale, range 0-15	1.0±1.3	1.1±1.4
3MS	96.1±4.1	96.0±4.0
Trails A, seconds	32.4±11.7	34.1±13.0
Trails B, seconds	77.5±36.7	88.3±46.0
Height, inches	65.2±3.9	65.6±4.0
Weight, pounds	174.3±39.4	174.4±35.6
Body mass index (kg/m^2)	28.8±5.8	28.5±5.8
Fear of falling	49 (41.5)	51 (41.8)
Fall prior year	35 (29.7)	36 (29.5)

© 2022 Brach JS et al. *JAMA Network Open.*

More than 1 fall	12 (10.2)	12 (9.8)
Global mobility rating		
Excellent	25 (21.2)	17 (13.9)
Very good	49 (41.5)	48 (39.3)
Good	33 (28.0)	41 (33.6)
Fair	11 (9.3)	15 (12.3)
Poor	0 (0.0)	1 (0.8)
Global health rating		
Excellent	16 (13.6)	22 (18.0)
Very good	63 (53.4)	53 (43.4)
Good	34 (28.8)	35 (28.7)
Fair	5 (4.2)	10 (8.2)
Poor	0 (0.0)	2 (1.6)
Global balance rating		
Excellent	5 (4.2)	7 (5.7)
Very good	26 (22.0)	28 (23.0)
Good	52 (44.0)	49 (40.2)
Fair	31 (26.3)	33 (27.1)
Poor	4 (3.4)	5 (4.1)
Instrumented walkway gait speed, m/s	1.09±0.15	1.07±0.17
Gait speed stratum		
<1.0 m/s	71 (60.2)	72 (59.0)
≥ 1.0 m/s	47 (39.8)	50 (41.0)
Modified Gait efficacy scale, range 10-100	86.0±13.1	85.1±13.6
Short Physical Performance Battery	9.9±1.4	9.8±1.8
Lower extremity strength 1 RM (greater of the two sides), newtons	184.4±55.3	188.5±61.5
Lower extremity power (greater of the two sides), watts	367.9±128.3	370.9±175.4
Six-minute walk distance, meters	402.8±89.9	400.9±88.5
Figure of 8 walk		
Time to complete, seconds	9.8±1.8	10.2±2.4
Number of steps	17.0±2.7	17.2±3.0
Chair reach test, centimeters	-5.1±11.0	-7.4±10.8
Late Life Function and Disability Index, range 0-100		

© 2022 Brach JS et al. *JAMA Network Open.*

Overall function	62.0±8.7	61.4±8.3
Upper extremity function	78.9±10.6	78.5±11.4
Basic lower extremity function	75.6±13.4	74.0±12.4
Advanced lower extremity function	52.6±13.7	51.8±13.3
Disability frequency	55.6±6.0	55.0±5.4
Social role	51.6±7.4	50.7±7.3
Personal role	68.8±15.5	67.6±13.8
Instrumental role	79.9±13.5	79.1±13.5
Management role	93.0±10.6	91.8±10.1
Disability limitations	79.9±12.8	79.1±12.9
Sedentary behavior, minutes/day	491.1±91.6	498.4±104.8
Moderate/vigorous activity, minutes/day	36.9±32.2	31.6±26.4
Steps, steps/day	4,314±2,186	3,977±2,250
Vector magnitude, counts/day	338,908±151,174	306,809±128,707

[a] Obtained using independent samples *t*-, chi-square or Fisher's exact test

© 2022 Brach JS et al. *JAMA Network Open.*

eTable 3. Intervention effects on secondary outcomes (representing components of intervention and additional mobility measures) stratified by screening gait speed.

	Outcome & Timeframe	Standard-Plus Mean±SD	Standard Mean±SD	Adjusted Difference±SE[b]	p-value[b]
**Slow walkers, (<1.0 m/s)**					
	Lower extremity strength change, newtons				
	12-weeks	7.1±25.8	1.4±24.5	-5.6±7.1	0.43
	24-weeks	-11.1±51.4	0.6±33.2	-11.3±7.7	0.15
	36-weeks	2.2±31.6	-1.0±32.0	-7.7±7.1	0.28
	Lower extremity power change, watts				
	12-weeks	-13.7±58.9	-14.0±67.0	-5.7±17.4	0.74
	24-weeks	-21.4±49.6	-13.1±67.3	-18.1±16.4	0.28
	36-weeks	11.2±70.0	-20.3±74.4	-16.3±20.6	0.44
	Six-minute walk distance change, meters				
	12-weeks	49.9±72.0[a]	38.7±59.7[a]	10.2±13.7	0.46
	24-weeks	33.5±71.5[a]	24.5±90.1	0.0±14.4	1.00
	36-weeks	40.6±74.3[a]	43.9±76.6[a]	5.8±15.1	0.70
	Figure of 8 time change, seconds				
	12-weeks	-1.0±1.8[a]	-1.6±2.7[a]	0.1±0.4	0.70
	24-weeks	-1.0±1.7[a]	-1.2±2.9[a]	0.0±0.4	0.94
	36-weeks	-0.9±1.8[a]	-1.5±3.0[a]	0.1±0.4	0.72
	Figure of 8 steps change				
	12-weeks	-1.0±2.7[a]	-1.6±2.7[a]	0.4±0.5	0.36
	24-weeks	-1.3±2.2[a]	-1.0±2.8[a]	0.0±0.5	0.95
	36-weeks	-1.0±2.2[a]	-1.3±2.6[a]	0.2±0.5	0.75
	Chair sit and reach change, centimeters				
	12-weeks	0.3±7.7	2.2±7.4[a]	-1.7±1.6	0.29
	24-weeks	0.5±7.2	3.5±9.7[a]	-1.8±1.7	0.29
	36-weeks	-2.0±8.0	0.1±9.3	-1.1±1.8	0.56
	Modified Gait Efficacy Scale change				
	12-weeks	-5.7±13.1[a]	1.5±10.7	-6.8±2.0	0.00
	24-weeks	-3.0±11.9	1.0±10.5	-3.9±2.0	0.05

© 2022 Brach JS et al. *JAMA Network Open.*

	36-weeks	-4.2±13.6[a]	1.6±10.5	-5.1±2.1	0.01
	Short Physical Performance Battery change				
	12-weeks	0.1±1.6	0.5±1.6[a]	-0.2±0.3	0.48
	24-weeks	0.1±1.6	0.3±1.5	-0.3±0.3	0.28
	36-weeks	-0.0±1.6	0.7±1.6[a]	-0.5±0.3	0.08
**Fast walkers (≥1.0 m/s)**	Lower extremity strength change, newtons				
	12-weeks	7.7±32.0	4.0±23.1	0.9±5.5	0.87
	24-weeks	1.6±25.8	2.7±25.2	-0.3±6.3	0.96
	36-weeks	7.3±23.4	-0.2±20.9	-1.4±5.8	0.81
	Lower extremity power change, watts				
	12-weeks	-14.5±54.7	2.0±57.6	-14.7±13.5	0.28
	24-weeks	-6.9±49.4	-2.5±54.9	-7.4±12.7	0.56
	36-weeks	-4.4±43.9	-8.6±66.4	-10.7±13.5	0.43
	Six-minute walk distance change, meters				
	12-weeks	33.0±56.2[a]	32.4±49.0[a]	-0.2±11.9	0.98
	24-weeks	30.7±47.8[a]	18.0±66.2[a]	12.7±12.8	0.32
	36-weeks	29.9±38.6[a]	13.2±67.7	13.0±12.0	0.28
	Figure of 8 time change, seconds				
	12-weeks	-1.0±1.5[a]	-0.7±1.3[a]	-0.3±0.3	0.23
	24-weeks	-0.7±1.4[a]	-0.4±1.6	-0.3±0.3	0.31
	36-weeks	-0.6±1.4[a]	-0.3±1.3	-0.1±0.3	0.65
	Figure of 8 steps change				
	12-weeks	-1.0±2.0[a]	-0.4±1.8	-0.6±0.4	0.12
	24-weeks	-0.8±2.1[a]	0.1±1.9	-0.7±0.4	0.08
	36-weeks	-0.5±1.6[a]	-0.3±1.6	-0.1±0.4	0.85
	Chair sit and reach change, centimeters				
	12-weeks	0.8±7.3	2.6±7.2[a]	-1.5±1.3	0.26
	24-weeks	2.6±7.9[a]	0.2±7.1	2.7±1.4	0.06
	36-weeks	0.2±7.7	-0.2±8.1	0.4±1.5	0.77

© 2022 Brach JS et al. *JAMA Network Open.*

	Modified Gait Efficacy Scale change				
	12-weeks	1.2±7.8	-1.4±11.0	2.4±1.7	0.16
	24-weeks	-0.2±8.0	-2.9±8.4[a]	2.3±1.7	0.18
	36-weeks	-0.9±10.6	-3.9±8.2[a]	2.2±1.7	0.21
	Short Physical Performance Battery change				
	12-weeks	0.2±1.4	0.3±1.1[a]	-0.2±0.2	0.41
	24-weeks	0.6±1.2[a]	0.6±1.2[a]	0.1±0.2	0.67
	36-weeks	0.4±1.3[a]	0.2±1.1	0.1±0.2	0.70

* p<0.05 obtained using paired samples *t*-test

[b]Obtained using a linear mixed model with multiple imputation for missing values

© 2022 Brach JS et al. *JAMA Network Open.*

eTable 4. Intervention effects on tertiary outcomes (Self-report activity and participation)

Participant Group	Outcome & Timeframe	Standard-Plus Mean±SD	Standard Mean±SD	Adjusted Difference±SE[b]	p-value[b]
**All**	LLFDI – Overall function change				
	12-weeks	0.2±5.9	0.3±4.9	-0.1±0.7	0.82
	24-weeks	-0.2±4.7	-0.7±4.5	0.5±0.7	0.46
	36-weeks	-0.4±5.6	-0.3±5.7	-0.1±0.7	0.83
	LLFDI – Upper extremity function change				
	12-weeks	-0.2±8.8	0.1±7.9	-0.3±1.1	0.81
	24-weeks	0.0±9.7	-1.2±9.0	1.1±1.2	0.33
	36-weeks	0.0±10.3	-0.9±10.5	1.0±1.2	0.38
	LLFDI – Basic lower extremity function change				
	12-weeks	-0.8±10.3	0.5±8.9	-0.9±1.1	0.44
	24-weeks	-0.3±10.1	-1.1±8.7	1.0±1.1	0.40
	36-weeks	-1.0±9.0	-1.0±9.0	0.2±1.1	0.87
	LLFDI – Advance lower extremity change				
	12-weeks	0.2±9.6	0.6±8.3	-0.3±1.1	0.75
	24-weeks	-0.4±7.5	-1.1±7.2	0.5±1.1	0.67
	36-weeks	-1.2±9.5	-0.4±8.5	-0.9±1.1	0.43
	LLFDI – Disability frequency change				
	12-weeks	-0.3±4.8	0.0±4.0	-0.2±0.6	0.70
	24-weeks	-0.2±4.4	-0.3±4.3	0.2±0.6	0.79
	36-weeks	-0.7±4.6	-0.6±4.2	-0.0±0.6	0.97
	LLFDI – Disability social role change				
	12-weeks	-0.3±6.4	-0.2±5.3	-0.0±0.8	0.98
	24-weeks	-0.8±6.7	-0.4±5.8	-0.5±0.8	0.50
	36-weeks	-1.2±6.1[a]	-1.0±6.0	-0.3±0.8	0.74
	LLFDI – Disability Personal role change				

© 2022 Brach JS et al. *JAMA Network Open.*

	12-weeks	-1.0±13.8	0.9±13.8	-1.2±1.7	0.49
	24-weeks	1.0±16.0	-0.9±14.5	2.7±1.7	0.13
	36-weeks	-0.3±16.7	0.3±12.4	0.9±1.8	0.63
	LLFDI-Disability Instrumental role change				
	12-weeks	-0.0±15.0	1.9±14.4	-1.6±1.8	0.38
	24-weeks	1.0±17.2	0.5±13.4	0.9±1.9	0.65
	36-weeks	0.5±15.9	-0.4±14.0	0.8±1.9	0.65
	LLFDI – Disability Management role change				
	12-weeks	-1.3±12.8	0.9±10.2	-1.3±1.3	0.32
	24-weeks	-1.0±12.3	0.5±11.0	-0.3±1.3	0.82
	36-weeks	-0.0±11.1	0.5±11.2	0.2±1.4	0.89
	LLFDI-Disability limitations change				
	12-weeks	-0.1±14.3	1.8±12.9	-1.6±1.7	0.34
	24-weeks	1.0±16.2	0.7±12.4	0.8±1.8	0.66
	36-weeks	0.9±15.1	0.2±13.1	0.7±1.8	0.68
**Slow walkers (<1.0 m/s)**	LLFDI – Overall function change				
	12-weeks	-0.8±6.2	1.1±4.4	-2.1±1.0	0.04
	24-weeks	0.1±4.3	-0.2±4.2	0.0±1.0	0.99
	36-weeks	-0.8±4.8	-0.6±5.8	-0.4±1.0	0.70
	LLFDI – Upper extremity function change				
	12-weeks	0.2±9.1	0.1±7.8	-0.7±1.7	0.68
	24-weeks	1.7±9.4	-2.0±9.4	2.5±1.7	0.15
	36-weeks	2.6±10.4	-2.6±11.6	4.1±1.7	0.02
	LLFDI – Basic lower extremity function change				
	12-weeks	-1.6±10.5	1.1±9.1	-2.9±1.7	0.09
	24-weeks	-0.4±9.6	0.0±9.3	-1.0±1.7	0.55
	36-weeks	-1.6±9.0	-1.1±9.6	-1.1±1.7	0.52

© 2022 Brach JS et al. *JAMA Network Open.*

	LLFDI – Advance lower extremity change				
	12-weeks	-0.7±9.4	1.9±7.6	-2.9±1.7	0.08
	24-weeks	-0.1±6.5	0.0±6.4	-0.3±1.7	0.84
	36-weeks	-2.6±9.2	-0.4±9.2	-2.2±1.7	0.20
	LLFDI – Disability frequency change				
	12-weeks	-1.2±4.9	-0.1±3.6	-0.9±0.9	0.29
	24-weeks	-0.1±3.5	-0.7±4.4	0.6±0.9	0.49
	36-weeks	-1.1±3.9[a]	-0.4±4.1	-0.4±0.9	0.66
	LLFDI – Disability social role change				
	12-weeks	-1.1±6.4	-0.1±5.0	-0.7±1.2	0.56
	24-weeks	-0.7±4.8	-0.7±6.1	0.05±1.2	0.97
	36-weeks	-1.8±5.0[a]	-0.4±6.1	-0.8±1.3	0.52
	LLFDI – Disability Personal role change				
	12-weeks	-3.3±13.8	0.1±12.1	-3.2±2.6	0.22
	24-weeks	0.8±13.7	-2.5±15.2	3.2±2.6	0.22
	36-weeks	-1.2±13.2	0.2±13.3	-0.8±2.7	0.76
	LLFDI- Disability Instrumental role change				
	12-weeks	1.7±15.6	2.5±13.7	-1.5±2.8	0.58
	24-weeks	1.5±13.1	0.8±11.0	0.1±2.9	0.98
	36-weeks	1.6±14.9	-0.4±13.1	0.3±3.2	0.92
	LLFDI – Disability Management role change				
	12-weeks	-0.9±14.7	-0.9±9.1	0.5±2.0	0.81
	24-weeks	-0.5±10.8	0.1±8.8	-0.3±2.0	0.89
	36-weeks	-1.0±10.0	0.0±10.2	-1.6±2.3	0.49
	LLFDI- Disability limitations change				

© 2022 Brach JS et al. *JAMA Network Open.*

	12-weeks	1.3±14.4	2.1±12.3	-1.2±2.6	0.63
	24-weeks	1.1±12.5	0.6±9.8	0.1±2.7	0.96
	36-weeks	1.4±13.7	0.1±12.1	-0.1±3.0	0.98
**Fast walkers (≥1.0 m/s)**	LLFDI – Overall function change				
	12-weeks	0.9±5.7	-0.2±5.1	1.3±0.9	0.14
	24-weeks	-0.4±5.1	-1.1±4.7	0.8±0.9	0.34
	36-weeks	-0.2±6.1	-0.1±5.7	0.1±0.9	0.95
	LLFDI – Upper extremity function change				
	12-weeks	-0.4±8.6	0.1±8.1	0.1±1.5	0.97
	24-weeks	-1.2±9.8	-0.6±8.8	0.2±1.5	0.91
	36-weeks	-1.8±9.9	0.3±9.6	-1.2±1.5	0.45
	LLFDI – Basic lower extremity function change				
	12-weeks	-0.1±10.1	0.0±8.8	0.7±1.5	0.65
	24-weeks	-0.2±10.5	-1.8±8.2	2.5±1.6	0.12
	36-weeks	-0.5±9.1	-0.9±8.5	1.2±1.5	0.43
	LLFDI – Advance lower extremity change				
	12-weeks	0.9±9.8	-0.4±8.7	1.5±1.4	0.28
	24-weeks	-0.7±8.3	-1.9±7.7[a]	1.1±1.4	0.46
	36-weeks	-0.2±9.7	-0.4±8.1	0.1±1.5	0.96
	LLFDI – Disability frequency change				
	12-weeks	0.5±4.6	0.1±4.4	0.3±0.7	0.67
	24-weeks	-0.2±5.0	-0.1±4.3	-0.2±0.7	0.83
	36-weeks	-0.4±5.1	-0.8±4.3	0.3±0.8	0.73
	LLFDI – Disability social role change				
	12-weeks	0.4±6.4	-0.3±5.6	0.5±1.0	0.64
	24-weeks	-0.9±7.8	-0.2±5.6	-0.9±1.0	0.35
	36-weeks	-0.8±6.8	-1.4±6.0	0.1±1.1	0.91
	LLFDI – Disability Personal role change				

© 2022 Brach JS et al. *JAMA Network Open.*

	12-weeks	0.8±13.7	1.5±15.0	0.4±2.2	0.87
	24-weeks	1.1±17.5	0.1±14.0	2.3±2.3	0.31
	36-weeks	0.4±18.9	0.4±11.9	2.1±2.3	0.35
	LLFDI-Disability Instrumental role change				
	12-weeks	-1.2±14.5	1.4±15.0	-1.6±2.3	0.51
	24-weeks	0.7±19.7	0.4±14.9	1.5±2.6	0.56
	36-weeks	-0.3±16.7	-0.4±14.6	1.2±2.5	0.62
	LLFDI – Disability Management role change				
	12-weeks	-1.6±11.3	2.2±10.8	-2.6±1.7	0.13
	24-weeks	-1.4±13.4	0.7±12.3	-0.3±1.8	0.87
	36-weeks	0.7±11.9	0.8±12.0	1.5±1.7	0.38
	LLFDI-Disability limitations change				
	12-weeks	-1.1±14.2	1.6±13.3	-1.8±2.2	0.41
	24-weeks	0.8±18.4	0.7±13.9	1.3±2.4	0.60
	36-weeks	0.5±16.2	0.2±13.9	1.3±2.3	0.57

LLFDI = Late Life Function and Disability Instrument

[a] $p<0.05$ obtained using paired samples *t*-test

[b] Obtained using a linear mixed model with multiple imputation for missing values

SD = Standard deviation

SE = Standard error

© 2022 Brach JS et al. *JAMA Network Open.*
