# Supplementary material for: Effect of Timing and Coordination Training on Mobility and Physical Activity Among Community-Dwelling Older Adults: A Randomized Clinical Trial
Source: JAMA Netw Open. 2022 May 23;5(5):e2212921. doi: 10.1001/jamanetworkopen.2022.12921 (PMC9127558; doi:10.1001/jamanetworkopen.2022.12921)
Supplement: Supplement 3. — Data Sharing Statement [file jamanetwopen-e2212921-s003.pdf]

Data Sharing Statement

Brach JS, Perera S, Shuman V, et al. Effect of Timing and Coordination Training on Mobility and Physical Activity Among Community-Dwelling Older Adults. *JAMA Netw Open*. 2022;5(5):e2212921. doi:10.1001/jamanetworkopen.2022.12921

Data
Data available: Yes
Data types: Deidentified participant data
How to access data: Data is available by emailing the PI at jbrach@pitt.edu
When available: With publication

Supporting Documents
Document types: None

Additional Information
Who can access the data: researchers who se proposed use of the data has been approved
Types of analyses: for any purpose
Mechanisms of data availability: with investigator support, after approval of a proposal, and with a signed data use agreement
